# Resolving intra-repeat variation in medically relevant VNTRs from short-read sequencing data using the cardiovascular risk gene *LPA* as a model

**DOI:** 10.1186/s13059-024-03316-5

**Published:** 2024-06-26

**Authors:** Silvia Di Maio, Peter Zöscher, Hansi Weissensteiner, Lukas Forer, Johanna F. Schachtl-Riess, Stephan Amstler, Gertraud Streiter, Cathrin Pfurtscheller, Bernhard Paulweber, Florian Kronenberg, Stefan Coassin, Sebastian Schönherr

**Affiliations:** 1 Institute of Genetic Epidemiology, Medical University of Innsbruck, Innsbruck, Austria; 2 Department of Internal Medicine I, Paracelsus Medical University/Salzburger Landeskliniken, Salzburg, Austria

**Keywords:** Variable number tandem repeat, VNTR, Lp(a), Dark genome region, Medically relevant gene, Whole-exome sequencing, UK Biobank, Nextflow

## Abstract

**Background:**

Variable number tandem repeats (VNTRs) are highly polymorphic DNA regions harboring many potentially disease-causing variants. However, VNTRs often appear unresolved (“dark”) in variation databases due to their repetitive nature. One particularly complex and medically relevant VNTR is the KIV-2 VNTR located in the cardiovascular disease gene *LPA* which encompasses up to 70% of the coding sequence.

**Results:**

Using the highly complex *LPA *gene as a model, we develop a computational approach to resolve intra-repeat variation in VNTRs from largely available short-read sequencing data. We apply the approach to six protein-coding VNTRs in 2504 samples from the 1000 Genomes Project and developed an optimized method for the *LPA* KIV-2 VNTR that discriminates the confounding KIV-2 subtypes upfront. This results in an F1-score improvement of up to 2.1-fold compared to previously published strategies. Finally, we analyze the *LPA *VNTR in > 199,000 UK Biobank samples, detecting > 700 KIV-2 mutations. This approach successfully reveals new strong Lp(a)-lowering effects for KIV-2 variants, with protective effect against coronary artery disease, and also validated previous findings based on tagging SNPs.

**Conclusions:**

Our approach paves the way for reliable variant detection in VNTRs at scale and we show that it is transferable to other dark regions, which will help unlock medical information hidden in VNTRs.

**Supplementary Information:**

The online version contains supplementary material available at 10.1186/s13059-024-03316-5.

## Background

Variable number tandem repeats (VNTRs) are frequent and highly polymorphic structural variants in the human genome consisting of repeated DNA sequences (≥ 7 bp) with a variable number of units among individuals [1]. VNTRs show an increased mutation rate compared to unique sequences in the genome and can be located in disease-causing genes [1, 2]. Since the complex and repetitive structure of VNTRs prevents unambiguous alignment of short reads and thus variant detection [3], VNTRs hide a considerable amount of medically relevant genetic variation from conventional sequencing approaches [4–6]. Elaborated mapping and variant calling approaches have allowed remarkable progress in resolving variation in these challenging gene regions, but some genes still cannot be resolved using these generalized approaches [4–7]. Previous work has identified dark or “camouflaged” regions which have been overlooked by standard approaches and outlined algorithms on how to resolve them [3, 8].

**Fig. 1:**
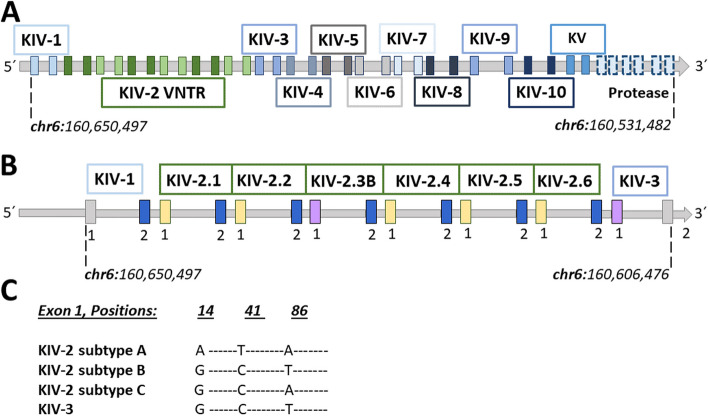
**A** *LPA* gene structure: each kringle domain (KIV-1 to KIV-10 and KV) consists of two exons and the protease domain of 6 exons. In both reference genome assemblies hg19 and hg38, the KIV-2 VNTR (green) is represented with 6 KIV-2 repeats, one being a KIV-2B. In 10,927 Central-European individuals from [13], the average, minimum, and maximum number of KIV-2 units measured at the protein level in plasma using Western Blot were 20, 2, and 36 KIV-2, respectively. **B** *LPA* KIV-1 to KIV-3: the numbers 1 and 2 in each KIV domain stand for exon 1 and exon 2, respectively. KIV-2 exons 1 and 2 are 160 bp and 182 bp long, respectively. The reference genome sequence shows 100% identity between the exon 2 sequence of KIV-1 and KIV-2 domains (blue) and between exon 1 sequence of KIV-2B and KIV-3 (purple) [2] (see the Introduction for more explanation of these homologies). **C** Alleles of the so-called KIV-2B canonical positions in different KIV domains: in the reference genome, KIV-2B and KIV-3 differ from the more frequent KIV-2A by the three PSVs in exon 1 at positions 14, 41, and 86 and about 100 intronic positions. In KIV-2C, positions 14 and 41 are identical to KIV-2B and position 86 is identical to KIV-2A

One important but still unresolved medically relevant VNTR [8] is located within the *LPA* gene. *LPA* controls > 90% of the plasma concentrations of lipoprotein(a) [Lp(a)] [9], which is likely the most important genetic risk factor for cardiovascular disease (CVD) in the general population, increasing CVD risk up to threefold [10]. *LPA* contains 10 highly homologous kringle IV (KIV) domains (Fig. 1A), whereas the KIV-2 domain represents a VNTR (Fig. 1B) with up to 40 repeats per allele [11]. It can encompass up to 70% of the *LPA* coding sequence [12]. Each KIV-2 unit is ≈5.6 kb large, creating an ≈200 kb large VNTR array, and a variant present in only one repeat may show as little as 1/80 = 1.25% fractional representation. Each KIV domain is encoded by two short exons (mostly 160 bp and 182 bp), spaced by a mostly ≈ 4 kb intron. A ≈1.2 kb intron links the single KIV domains. The exons of the KIV-2 present up to 98% base identity to the respective exons of the flanking KIV units, especially KIV-1 and KIV-3 [2]. The second exon of KIV-1 is identical to the second KIV-2 exon and ≈70% homology is observed even within the first 200 intronic bases flanking the exons. This complicates the definition of the VNTR boundaries, read alignment, and mutation detection. Moreover, the KIV-2 domain exists in at least three nearly identical subtypes (denoted by KIV-2A, B, and C), which differ in the haplotype of three synonymous variants, as well as of > 100 intronic variants [11] (Fig. 1C). The first exon of KIV-2B is identical to the first exon of KIV-3, while the respective second exons show 96% base identity. Misaligned reads belonging to other KIV-2 subtypes or to other highly homologous KIV units (like KIV-1, KIV-3, KIV-4) [2] can create spurious variant calls originating from sequence differences between the domains (paralogous sequence variants, PSVs [8]).

**Fig. 2:**
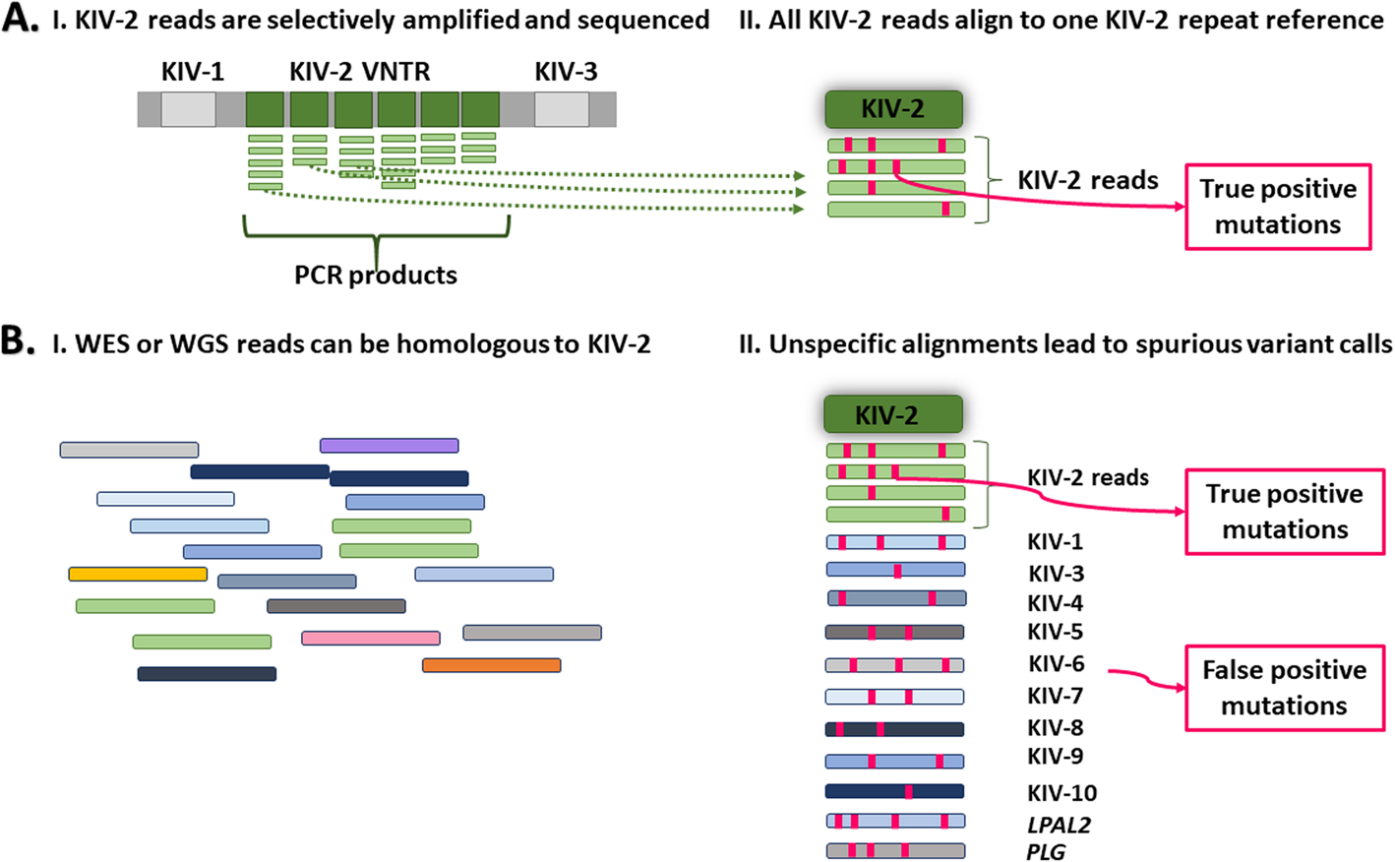
Variant calling in the KIV-2 VNTR of the *LPA* gene. **A** Targeted KIV-2 sequencing approaches selectively amplify all KIV-2 repeats by leveraging the high inter-repeats homology and exclude other kringles (I). All KIV-2 reads are aligned to a single KIV-2 reference repeat and KIV-2 mutations are called as low-level mutations (II). **B** In WES or WGS data, KIV-2 reads are mixed with reads originating from other kringles and homologous regions (I). When aligning the reads to a single KIV-2 reference repeat for variant calling as in previous approaches [2], reads that are nearly identical to KIV-2 can align and cause spurious variant calls (II)

These multiple complexities impair mutation screening in *LPA,* which thus appears unresolved (“dark”) in available variation databases [2, 3], and even led to its exclusion from the latest benchmark datasets on variation in complex genome regions [4–7]. To circumvent these difficulties, we have previously sequenced the KIV-2 repeats using targeted KIV-2 amplicons that amplify only the KIV-2 units in a highly specific manner [2] (Fig. 2A). After deep sequencing, all reads from these amplicons have been aligned to one KIV-2 repeat and KIV-2 mutations called as low-level mutations, excluding confounding reads from homologous regions. This showed that the KIV-2 region is highly variable [2, 12, 14, 15] and that even highly frequent functional variants had been long missed due to their location in the KIV-2 VNTR (4925G > A [12], carrier frequency ≈22%, and 4733G > A, carrier frequency ≈38% [15]).

In this paper, we combine previously described approaches [3, 8] and provide a computational approach to call variants in VNTRs from short-read sequencing data at scale using the highly complex KIV-2 VNTR in *LPA* as an ideal candidate for set-up and benchmarking. We then successfully applied this approach to the 1000 Genome WGS data of five other medically relevant VNTRs (*NEB*, *DMBT1*, *FLG*, *SPDYE3*, and *UBC*) [8, 16]. The *NEB* gene has been associated with different kinds of myopathies [17] and contains one of the longest VNTR repeat units of the human genome (> 10 kb) [8]. *DMBT1*, *FLG*, *SPDYE3*, and *UBC* encompass protein-coding VNTRs and have been associated with various traits including familial glioma [18], atopic dermatitis [19], refractive errors [20], and HDL cholesterol [21], respectively.

The extensive homologies across the *LPA* gene and between the KIV-2 subtypes pose additional complexities to variant calling in the KIV-2 VNTR from whole-genome sequencing (WGS) or whole-exome sequencing (WES) data, since they contain reads from all KIV units. They are thus particularly prone to spurious variant calls caused by incorrectly aligned reads originating from homologous KIV units and/or from different KIV-2 subtypes (Fig. 2B). We used our highly specific amplicon-based dataset [2] as a gold-standard to evaluate and improve variant calling in the *LPA* KIV-2 VNTR from WGS or WES data. To this end, we devised a strategy to improve the accuracy of *LPA* KIV-2 variant calling by leveraging specific PSVs to inform the optimal variant calling strategy in a dynamic sample-specific manner. Finally, we applied this strategy to > 199,000 WES samples from UK Biobank providing the so far largest analysis of the *LPA* KIV-2 VNTR.

## Results

We present a computational approach to resolve intra-repeat variation in VNTRs from short-read sequencing data, and, in particular, in the *LPA* KIV-2 VNTR, which is a particularly difficult target due to the presence of highly homologous domains flanking the VNTR region. We leveraged our previous *LPA* work [2, 12, 14, 15, 22, 23] to provide a general, easy-to-use computational workflow to call base variation in VNTR repeats and to specifically resolve variation within the complex *LPA* KIV-2 VNTR. Our workflow isolates the reads of the targeted VNTR and we applied it without adaptations to 5 other protein-coding VNTRs (*NEB*, *DMBT1*, *FLG*, *SPDYE3*, and *UBC*) in 2504 samples from the 1000 Genome Project (1kGP). Our experiments in the *LPA* gene revealed that differences in the read extraction strategies can strongly affect the variant calling performance, with more stringent coordinates leading to the most accurate results. Moreover, we exemplify the use of SNPs in the flanking non-repetitive region to identify KIV-2 VNTR subtypes on an individual basis and dynamically adapt the VNTR reads identification.

### Computational workflow for variant calling in VNTRs

**Fig. 3:**
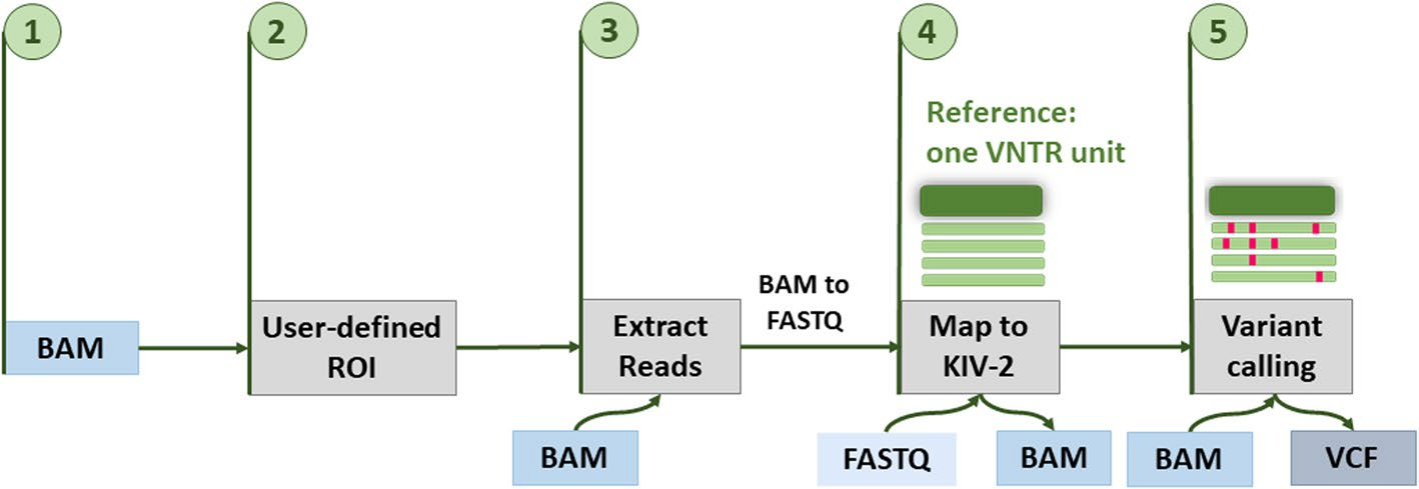
Workflow of the variant calling approach in VNTR regions from short-read sequencing data: the input file is provided in BAM format, with reads aligned to the reference genome (step 1); the region of interest (ROI) defined by the user (provided as, e.g., BED file) is selected (step 2); the VNTR reads are extracted from the provided ROI (step 3); the extracted reads are realigned to one reference repeat (step 4) for variant calling with mutserve (step 5), resulting in a VCF output file that can subsequently be annotated (see GitHub repository in the Availability of data and materials section)

The input of our workflow (Fig. 3) are files where all WES/WGS reads are pre-aligned to the human reference genome in BAM format. All reads mapped to a user-definable VNTR region-of-interest (VNTR ROI) are extracted and remapped to a reference sequence consisting of one single repeat unit. Variants in single VNTR units are then present only in a subset of reads, resembling somatic mutations, and are thus called using our mutserve variant caller [2, 24] with optimized settings for low-level variant detection (Methods). For a simplified and scalable application to user-defined VNTRs, the pipeline has been implemented using the Nextflow workflow manager [25].

### Construction and benchmarking of the variant calling workflow using the *LPA* KIV-2 VNTR

**Fig. 4:**
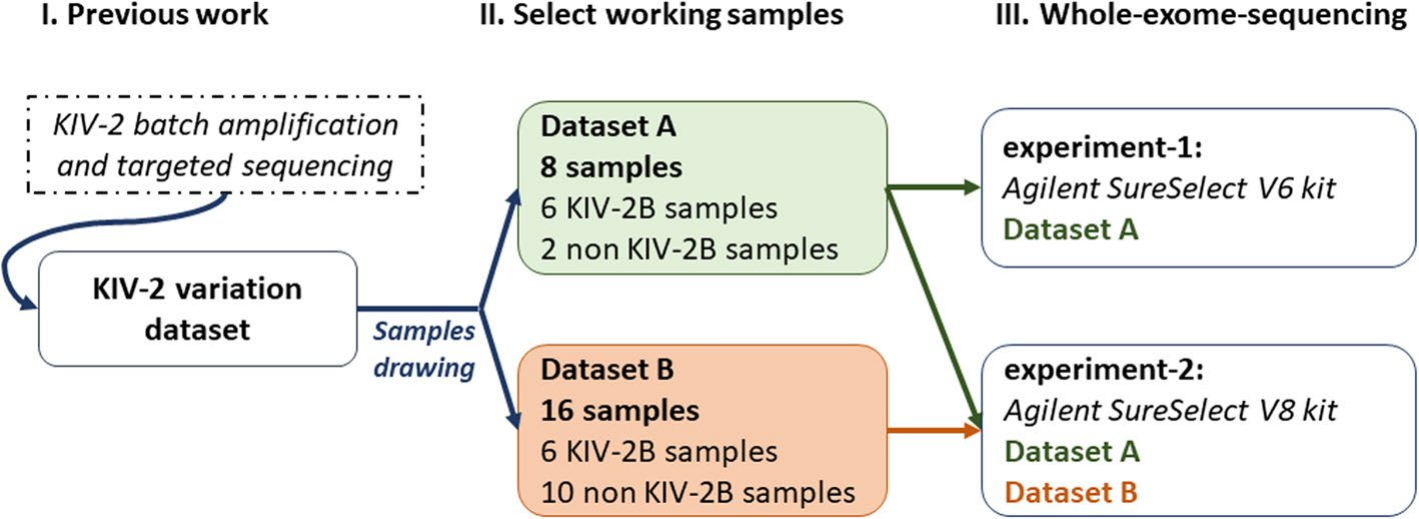
From the previously generated KIV-2 variation dataset with known KIV-2 variants [2] (I), we selected 8 samples (dataset A) for the setup of our KIV-2 variant calling pipeline in exome data and 16 samples (dataset B) for benchmarking (II). Subsequently, we generated WES data (III) in two sequencing experiments (experiment-1 and experiment-2), using two different exon enrichment kits, namely Agilent SureSelect v6 kit (experiment-1) and v8 kit (experiment-2). Dataset A was sequenced in both experiments, and the obtained WES data are called dataset A experiment-1 and dataset A experiment-2. Samples from dataset B were processed only in experiment-2 (v8 kit)

We generated WES data for two datasets (dataset A, dataset B), with known KIV-2 variant patterns from our previously established KIV-2 variation dataset [2] (referred to as gold-standard. See Methods for a brief description of the gold-standard generation). We performed two independent sequencing experiments (experiment-1 and experiment-2) using different exon enrichment chemistries (AgilentSure Select v6 kit and v8 kit). Dataset A (*n* = 8) was sequenced in both experiments and was used for the pipeline setup. Dataset B (*n* = 16) was sequenced only in experiment-2 and was used for benchmarking purposes (Fig. 4). The KIV-2 target region in our experiments spans 100 bp upstream and downstream of each of the two KIV-2 exons. In our gold-standard dataset, the number of coding KIV-2 variants per sample ranged from 4 to 24 in dataset A and from 2 to 17 in dataset B (Additional file 1: Table S1). About 80% of Europeans contain KIV-2B units, which can be recognized based on the distinctive haplotype at the canonical positions 14, 41, and 86 of KIV-2 exon 1 (corresponding to positions 594, 621, and 666 in our reference sequence). Two samples of dataset A and ten samples of dataset B were classified as non-KIV-2B samples. Datasets A and B served as data for setup and benchmarking of our workflow. In particular, the enrichment for non-KIV-2B samples in dataset B allowed the investigation of strategies to account for the presence/absence of KIV-2B subtypes in the variant calling workflow for the KIV-2 VNTR.

**Fig. 5:**
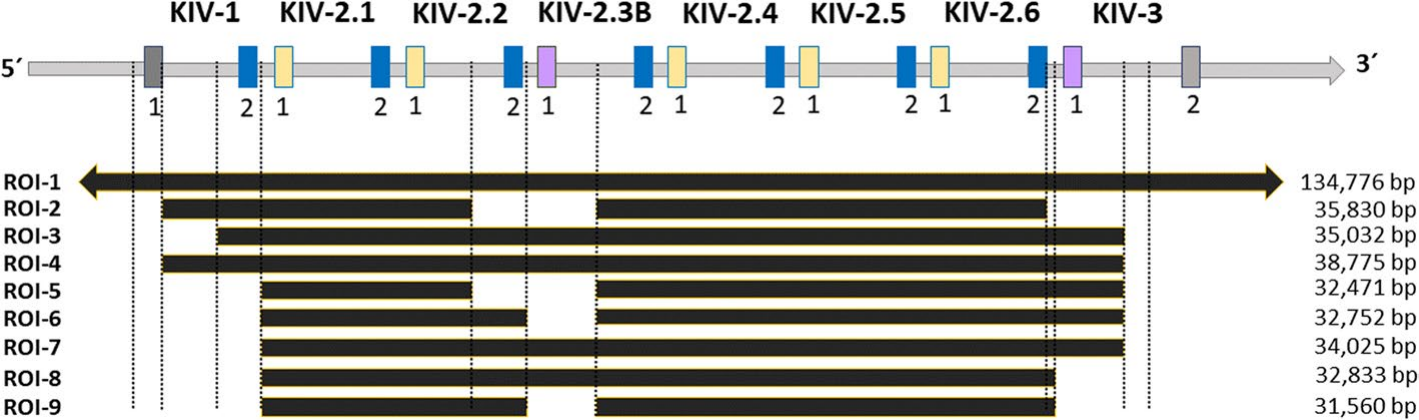
Region of Interest (ROI) for KIV-2 read extraction from aligned WES data. The *LPA* gene is represented from KIV-1 to KIV-3 in transcription direction and not at scale for clarity. The KIV-2 VNTR is represented with 6 repeats as in the reference genomes hg19 and hg38, with the third repeat in the transcription direction being a KIV-2B unit. The other KIV-2 units are KIV-2A (in each unit, the first exon is represented in yellow, the second exon in blue). The KIV-2B unit in the VNTR is highly homologous to the KIV-3, which flanks the KIV-2 VNTR, showing the same first exon (purple) and 96% homology in the second exon. Additionally, all second KIV-2 exons are identical to the second KIV-1 exon (blue, see also the supplementary Fig. 12 of ref [2] for a detailed per-base identity matrix). In the first 200 intronic bp flanking each exon, 70–100% per-base identity is observed between KIV-1, KIV-2, KIV-2B, and KIV-3. Thus, minor differences in the ROI used for read extraction can extract highly homologous reads that create spurious variants if aligned to only one repeat as done in this variant calling pipeline. ROI-1 extracts reads from the complete *LPA* region (from KIV-1 to the protease domain, represented by an arrow). ROI-2 to ROI-9 progressively restricts the extraction region to the KIV-2 VNTR only. Since the KIV-2B appears fixed in the human genome reference, ROI-2 (previously published in [3]) and ROI-5 do not extract reads mapped to the KIV-2.2 exon 2 and KIV-2.3 subtype B, while ROI-6 and ROI-9 do not extract reads mapped to the KIV-2.3 subtype B. In combination with the presence or absence of KIV-2B units in the sequenced sample, this creates different spurious variant calls in a sample-specific manner. The size of the regions enclosed by each ROI is provided as bp between the start and end coordinates on hg38. Precise coordinates are provided in Additional file 1: Table S3

First, we tested a naive KIV-2 variant calling approach by aligning all available WES reads to a single KIV-2 repeat. However, due to the extensive homologies of the KIV-2, also many other reads from other kringles and other homologous regions (e.g., *PLG*, *LPAL2*) aligned unspecifically and generated spurious variant calls originating from PSVs [8]. In experiment-1 of dataset A, over 200 false positive variants per sample were called (Additional file 1: Table S2). This suggested that it would be necessary to restrict read extraction to the KIV-2 VNTR. However, the KIV-2 VNTR resembles a degenerated VNTR array, with a rather conserved core VNTR region (the KIV-2 domains), flanked by highly similar domains (KIV-1 and KIV-3). Additional complexity is added by the facts, that the exons of the KIV-2B units (which are located within the KIV-2 VNTR array) are identical to the KIV-3 (which flanks the VNTR array) and that KIV-2B units are not present in all human individuals. This has led to varying definitions of the VNTR region across literature [2, 3, 8, 26]. We therefore systematically assessed the KIV-2 variant calling performance of 9 different regions-of-interest to extract KIV-2 reads (ROI-1 to ROI-9) using both experiments of dataset A (Fig. 5). The ROIs where selected either from our own previous research [2], from literature [3, 8], or represented various curated combinations thereof (Additional file 1: Table S3). The combinations where designed manually taking into consideration the various homologies described in the introduction and resulting in possible read mismappings (see below).

Variations in the extraction region impacted the F1-score (F1), which describes the harmonic mean of precision (true positives/(true positives + false positives)) and sensitivity (true positives/(true positives + false negatives)) [27]. Thus, F1 reflects both false negative and the false positive calls and performs well in unbalanced datasets. A higher F1 score indicates better performance. Overall, the F1 ranged from 5 to 100% for experiment-1 and from 4.5 to 100% for experiment-2 (Additional file 1: Table S4). Strategy ROI-1, where reads were naively extracted from the complete *LPA* region, led to a very low mean F1 of ≈15% in both sequencing experiments, mainly driven by the high rate of false positive variants. The KIV-1 and KIV-3 domains share the highest homology with KIV-2 [2] and are therefore more likely to sequester KIV-2 reads in the initial alignment step. Thus, some of our tested strategies (ROI-2 to ROI-7) include various combinations of KIV-1 and KIV-3 regions. The strategies ROI-2 and ROI-3 were previously used by Ebbert et al. [3] and by Mukamel et al. [8]. Compared to ROI-1, using ROI-2 to ROI-7 already considerably improved the variant calling by excluding all other kringles and the mean F1 in dataset A ranged from 31.0 to 70.1% (Table 1 and Additional file 1: Table S4). When extracting only reads strictly mapped to the KIV-2 VNTR (ROI-8), without flanking regions, the mean F1 further improved to 81.9% for experiment-1 and to 84.4% for experiment-2. This suggested that the region of extraction has a major impact on variant calling and that more stringent coordinates, limited to the VNTRs start and end coordinates, reduce the false positive calls and improve the variant calling performance. Nevertheless, the results of ROI-8 still showed a large variance at individual level in both experiments, with standard deviation (SD) of 21.5–24%. Therefore, we further investigated potential stratifications within our datasets.

**Table 1:** F1-score (%) in dataset A experiment-1 and experiment-2 for each tested ROI strategy expressed as mean ± standard deviation (SD) and as median (minimum–maximum). ROI-8 shows the highest F1 mean in both experiments. The high standard deviation is explained by the high rate of false positive variants in the non-KIV-2B samples, which range from mean = 0 with ROI-9 (both experiments 1 and 2) to mean = 152 and mean = 164 with ROI-1 (experiments 1 and 2, respectively). See Additional file 1: Table S4 for performance metrics at the individual level

	**Dataset A experiment 1 (in %)**		**Dataset A experiment-2 (in %)**	
	**Mean ± SD**	**Median (min–max)**	**Mean ± SD**	**Median (min–max)**
**ROI-1**	15.7 ± 6.3	16.8 (5.0–25.7)	14.9 ± 6.3	16.2 (4.5–24.6)
**ROI-2**	42.7 ± 20.7	43.2 (15.4–85.7)	30.9 ± 15.7	30.3 (11.8–62.5)
**ROI-3**	68.3 ± 21.7	74.7 (28.6–98.0)	69.6 ± 23.6	75.7 (29.6–98.0)
**ROI-4**	62.1 ± 21.9	68.4 (21.1–80.0)	61.6 ± 22.9	68.0 (21.6–91.4)
**ROI-5**	68.3 ± 21.7	74.7 (28.6–98.0)	68.0 ± 24.2	74.6 (24.0–98.0)
**ROI-6**	68.3 ± 21.7	74.7 (28.6–98.0)	70.1 ± 23.2	76.7 (30.8–98.0)
**ROI-7**	68.3 ± 21.7	74.7 (28.6–98.0)	69.9 ± 23.7	76.7 (29.6–98.0)
**ROI-8**	81.9 ± 22.4	95.0 (40.0–100.0)	84.4 ± 20.1	96.5 (42.1–100.0)
**ROI-9**	59.3 ± 28.3	52.6 (25.0–100.0)	53.0 ± 27.9	43.5 (25.0–100.0)

### Accounting for repeat subtypes improved variant calling in the *LPA* KIV-2 VNTR

In the reference assemblies hg19 and hg38, the KIV-2 VNTR region is represented with 6 KIV-2 repeats, which are 5 KIV-2A units but only one KIV-2B. Thus, the KIV-2B repeat subtype erroneously appears fixed and in a single copy, while at the population level the intra-individual frequency of KIV-2B units changes widely, and even many “non-KIV-2B individuals” exist, which carry zero KIV-2B repeat units [2, 9, 26]. We noticed that ROI-8 performed best only in KIV-2B individuals with a F1 between 94.2 and 95.0% for both experiments. In non-KIV-2B individuals, the mean F1 dropped to 45.0% for experiment-1 and to 52.3% for experiment-2 and resulted in a high number of false positive variants (Table 2). Compared to ROI-8, ROI-9 does not extract reads mapped to the first exon of the KIV-2B repeat. Since no true KIV-2B reads exist in non-KIV-2B individuals, the reads that are aligned in these individuals to the KIV-2B repeat of the reference genome must originate from highly homologous domains, most likely from KIV-3, which is nearly identical to the KIV-2B. Thus, in non-KIV-2B individuals, ROI-9 skips the KIV-2B domain in the reference during read extraction and so reduces false positive calls. ROI-9 improved the F1 in non-KIV-2B individuals to 95.5% and to 100% in the two sequencing experiments in dataset A (Discussion). We thus investigated strategies to minimize the false positive rate by accounting for the presence/absence of KIV-2B subtypes in a dynamic way within the Nextflow analysis pipeline.

**Table 2:** Comparison between the F1-score in each sample of dataset A in experiment-1 and experiment-2 using ROI-8 and ROI-9. ROI-8 performs best in KIV-2B samples and ROI-9 performs best in non-KIV-2B samples. The sign “- “ is used for “no calculation possible”

	**Dataset A experiment 1 (in %)**		**Dataset A experiment-2 (in %)**	
	**ROI-8**	**ROI-9**	**ROI-8**	**ROI-9**
**Non-KIV-2B**	40.0	100.0	42.1	100.0
**Non-KIV-2B**	50.0	100.0	62.5	90.9
**KIV-2B**	93.3	52.6	96.6	35.3
**KIV-2B**	100.0	47.6	100.0	47.6
**KIV-2B**	96.6	25.0	96.6	25.0
**KIV-2B**	78.0	28.6	80.0	28.6
**KIV-2B**	97.3	61.5	97.1	43.5
**KIV-2B**	100.0	-	100	-

**Fig. 6:**
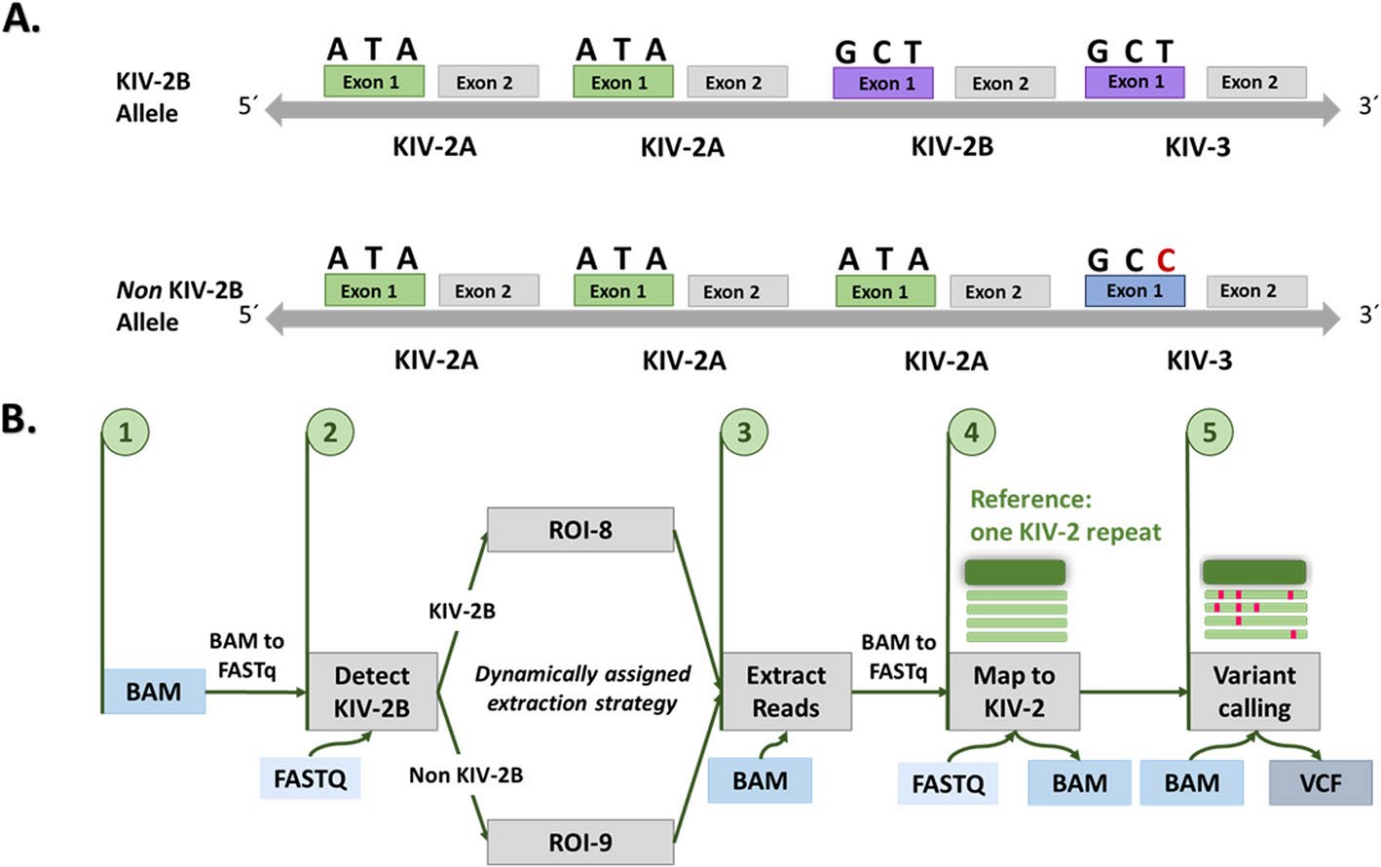
**A** Linkage disequilibrium between the KIV-2B haplotype and the genotype at position 86 of KIV-3 exon 1. For each KIV domain represented, exon 1 and exon 2 are shown. The bases shown above each exon 1 corresponds to positions 14, 41, and 86 (KIV-2B canonical positions). We identified a C at position 86 in KIV-3 exon 1 (CC genotype) in non-KIV-2B individuals. KIV-2B alleles present a T at position 86 in KIV-3 (corresponds to the reference sequence) while non-KIV-2B alleles would carry the C, resulting in a CC genotype in 70% of the non-KIV-2B individuals. **B** Workflow of the signature-based approach for KIV-2 variant calling in WES data: starting from the input aligned BAM file (step 1), the entire *LPA* region is screened in FASTQ format for the KIV-2B signature sequence and (step 2) each sample is redirected to the optimal ROI strategy for (step 3) KIV-2 read extraction. (step 4) The extracted reads are realigned to one reference KIV-2 repeat for (step 5) variant calling with mutserve, resulting in a VCF output file, which can subsequently be annotated (see Github repository in the Availability of data and materials section)

Our experiments showed that ROI-8 and ROI-9 are the optimal strategies for variant calling of KIV-2B and non-KIV-2B samples, respectively. Therefore, we searched for a unique sequence feature to discriminate KIV-2B and non-KIV-2B individuals agnostically before read extraction and assign the best ROI dynamically. Although the first exon of KIV-3 is reported in the literature as identical to the first exon of KIV-2B, we identified a SNP at position 86 in KIV-3 exon 1 (referred to as “signature position”) that separates KIV-2B from non-KIV-2B individuals (Additional file 2). To this end, we sequenced the KIV-3 region in 66 samples with known KIV-2 variant patterns [2] using a Sanger sequencing protocol designed to differentiate KIV-3 from KIV-2 and KIV-2B, as well as other KIV domains (50 KIV-2B samples and 16 non-KIV-2B) (Additional file 2; for considerations regarding the specificity of the KIV-3 PCR and Sanger sequencing Additional file 3). All KIV-2B samples carried at least one T-allele (which is also the reference base in the genome) at the signature position, while almost 70% of the non-KIV-2B individuals (11 of 16 samples) carried a C/C homozygous genotype at this position (Additional file 1: Table S5). This indicates a correlation between the presence of KIV-2B repeat units and a T-allele at position 86 of KIV-3 exon 1 (T-signature), respectively the absence of the KIV-2B units and a C-allele at this position (Fig. 6A). Accordingly, we observed a strong LD between the bases at KIV-2 position 594 (used as proxy for KIV-2B absence/presence) and position 86 in KIV-3 exon 1 (Lewontin’s D′ = 0.9995; indicating absence of recombination between the loci). A detailed explanation about LD investigation for KIV-2 SNPs is provided as Supplementary Note in Additional file 4.

We therefore defined a KIV-2B signature sequence (CCACTGTCAC**T**GGAA) that encompassed the T-allele at position 86 of KIV-3 exon 1 (Additional files 2–4 and sequence alignments in Additional file 5). We hypothesized that the presence or absence of this signature sequence in the sequencing reads allows discriminating between sample subtypes (KIV-2B and non-KIV-2B) and assigning each sample to the best ROI for read extraction. We used the available WES data from the non-KIV-2B samples (*n* = 12) to calculate an empirical threshold of signature-sequence occurrences to distinguish between the two sample subtypes. First, we counted the number of signature sequence occurrences within each FASTQ input file. Subsequently, the mean value plus 2 standard deviations of all occurrences across all non-KIV-2B samples was calculated and used as a threshold (Additional file 1: Table S6) to dynamically assign each sample to either ROI-8 (signature coverage above the threshold) and ROI-9 (signature coverage below the threshold).

We integrated our findings in an *LPA-*specific variant calling pipeline (here referred to as signature-based approach) that improves variant calling in the KIV-2 VNTR at scale by integrating a screening step for the KIV-2B signature sequence (Fig. 6B). The dynamic ROI assignment minimized the number of erroneously called mutations especially in non-KIV-2B samples, where a string of KIV-2B specific positions originating from KIV-3 would otherwise be detected as false positive variants (Additional file 1: Table S7). The mean F1 of the dynamic ROI assignment was ≈95% in both experiments. Dataset A includes two non-KIV-2B samples and the mean F1 obtained with the dynamic ROI assignment increased by 16.8% for experiment-1 (from 81.9 to 95.65%) and by 12.7% for experiment-2 (from 84.4 to 95.14%), also showing less variability among samples (Additional file 1: Table S8). Importantly, the signature sequence-based approach did not call false positive variants in the non-KIV-2B samples, while up to 12 false positive variants were called with the static ROI-8 approach.

For benchmarking the signature-based approach, we applied it to a second and independent dataset B (*n* = 16), including 10 additional non-KIV-2B samples and assessed its performance. The signature-based approach correctly classified all 6 KIV-2B samples and 9 of 10 non-KIV-2B samples. The single misclassification was one non-KIV-2B individual that presented a T/T genotype at the signature position. This was the only sample in our Sanger-sequenced dataset (*n* = 66) that presented a T/T genotype at KIV-3 exon 1 position 86 despite the absence of KIV-2B repeats. As a result, this sample was processed with ROI-8 despite being a non-KIV-2B sample. For all other samples, the signature-based approach did not detect any false positive variants in the samples.

**Fig. 7:**
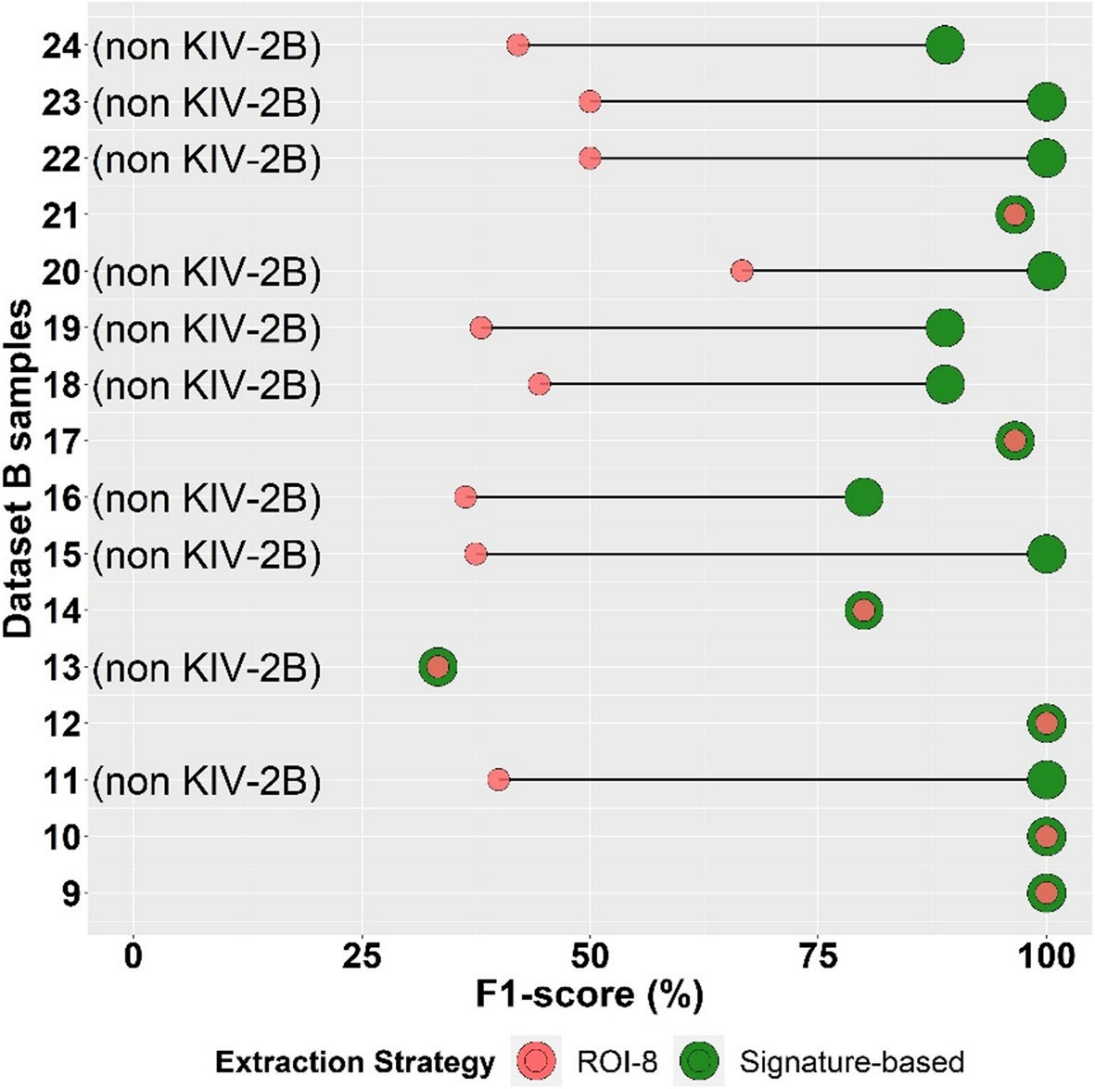
Benchmarking the signature-based approach in dataset B. The signature-based approach improved the F1-score in 9 out of 10 non-KIV-2B samples and showed the same accuracy for sample number 13. All KIV-2B samples (samples 9, 10, 12, 14, 17, and 21) resulted in the same F1-score using the signature-based approach

For dataset B, when using the dynamic signature-based approach the F1 increased from 63.2 to 90.8% (+ 43.6%; compared to ROI-8 only) and showed less variability among samples (Fig. 7). After excluding the misclassified sample, the F1 reached 94.7% (Additional file 1: Table S9).

### Variant calling in VNTRs in the 1000 Genomes Project

The ROIs experiments for *LPA* KIV-2 VNTR showed that, when the information about the repeats subtypes is not considered, not available or not relevant, bona fide VNTR start and end coordinates (ROI-8) led to the highest mean F1 score. In 2021, Mukamel et al. [8] identified 118 protein-coding VNTRs in medically relevant genes of the human genome and provided bona fide VNTR start and end coordinates identified with various VNTR finder tools [8]. Thus, these coordinates can be used as ROI for read extraction to call variants in other VNTRs. Therefore, we used our pipeline (Fig. 3, i.e., without the signature-based step) to analyze and annotate the protein-coding VNTRs in five other medically relevant genes (*NEB*,* SPDYE3*,* FLG*,* UBC*,* DMBT1*) in 2504 unrelated samples of the 1kGP [16]. A single VNTR repeat unit as defined in [8] was used as a reference. Since the *LPA* gene was also included in the list from Mukamel and colleagues, we first analyzed the *LPA* KIV-2 VNTR in 1000G data using as ROI the start and end coordinates generated by Mukamel et al. [8].

The final call set for each analyzed VNTR using the 1kG samples is provided at the pipeline repository (https://github.com/genepi/vntr-calling-nf) and represents the fully resolved call set of variants within the six analyzed VNTRs. In all analyzed VNTRs, we identified and annotated potentially functional variants, which can serve as a starting point for gene-specific validations (Table 3). Potential missense mutations have been identified in all genes (ranging from 7 to 321), nonsense mutations in 5 genes (*LPA*,* DMBT1*,* NEB*,* FLG*,* SPDYE3*,* UBC*), splice-site mutations and slicing region mutations in 3 (*LPA*,* DMBT1*,* NEB*).

**Table 3:** Analysis of the VNTRs in 6 genes in 2504 WGS samples from 1kGP. The regions of interest (ROI) for VNTR read extraction were defined as in [8]. VNTR variants detected at least once in the 1kGP samples are divided by mutation type. The percentage of paralogous sequence variants (PSVs), i.e., sequence differences among the repeats, was calculated as described in the Methods section based on the reference sequence of the human genome and excluding degenerated flanking repeats for *LPA*, *DMBT1*, and *UBC*. For each mutation category, we report the number of unique identified variants, excluding PSV positions. As the VNTRs in *FLG*, *SPDYE3*, and *UBC* are intra-exonic, no splice-site mutations nor splicing region mutations were involved

	***LPA***	***DMBT1***	***NEB***	***FLG***	***SPDYE3***	***UBC***
**VNTR ROI, GRCh38 coordinates**	160605062–160647661	122599445–122617454	151577914–151609576	152303180–152314028	100308203–100315286	124911751–124913720
**Estimated Repeat Length**	5104	4059	10,553	*972*	1390	228
**Percentage of PSVs between VNTR repeats**	2.15%	0.02%	3.74%	26.69%	4.84%	11.40%
**Missense mutations**	128	21	89	321	28	7
**Nonsense mutations**	9	1	1	27^a^	1	0
**Splicing region mutations**	46	13	0	NA	NA	NA
**Splice-site mutations**	3	1	41	NA	NA	NA

^a^Might be caused by homologous regions elsewhere in the genome and might require individual validation. Region coordinates in hg the human genome reference sequences hg38 and hg19 are provided in Additional file 1: Table S17

From our observations based on the presence or absence of the KIV-2B subtype in the *LPA* KIV-2 VNTR, we assumed that the position of the ROI and the existence of different repeat subtypes within VNTRs may generally increase the risk of false positive calls caused by PSVs. To exclude them from downstream analyses, we thus compiled a list of existing PSVs between the repeats for each investigated VNTR (https://github.com/genepi/vntr-calling-nf) and estimated the expected conserved bases for each VNTR (Table 3). In genes like *LPA*,* DMBT1*, and *UBC*, the high homology between the VNTR and the flanking regions (“degenerated repeats”) complicates the identification of the bona fide VNTRs start and end coordinates and considerably increases the percentage of PSVs detected. In these genes, the exclusion of such degenerated repeats minimized the PSVs expected as false positive (Table 3). Overall, the repeat length ranged from 228 bp to 10,553 bp with a PSV count of 0.02% (*DMBT1*) to 26.69% (*FLG*).

Eight known KIV-2 variants were used to benchmark our results in *LPA* (Additional file 1: Table S10). Using the ROI defined in [8] as a single region of extraction, independent of the KIV-2B presence or absence, we successfully detected biologically functional variants like the Lp(a)-lowering splice-site mutation 4925G > A [12] and 4733G > A [15] (carrier frequency of 9.7% and 12%, respectively, consistently with previous findings [2]). We also successfully called three other functional variants recently identified [8] and the three synonymous variants at positions 14, 41, and 86 in KIV-2 exon 1 which define the KIV-2 subtypes (named KIV-2A, KIV-2B, and KIV-2C [11]). However, the three KIV-2B canonical variants showed a much higher carrier frequency (~ 100%, ~ 100%, and 77.6%, respectively) than reported [2], suggesting an increased rate of false positive calls at these positions. In line with this assumption, the signature-based approach with dynamic assignment of the experimentally defined ROI-8 and ROI-9 lowered the KIV-2B canonical variants carried frequencies considerably to ≈54% (Additional file 1: Table S10) and reduced the noise (Additional file 6: Fig. S1). These frequencies are much more consistent with findings in the literature [2], further supporting an improved discrimination of false positive calls at these positions.

As a technical note, we were able to run the pipeline on the 1000 Genomes Project data in 33 min (total CPU hours: 7.9) assigning a memory requirement of 1 GB to each process (Additional file 7).

### *LPA* KIV-2 variant detection in UK Biobank

We analyzed the KIV-2 VNTR in 199,119 samples from the UK Biobank whole-exome sequencing (UKB 200K WES release) by applying our defined signature-based workflow (Additional file 1: Table S11). The KIV-2B signature sequence was detected in 75% of the samples (*n* = 149,184), with individuals of Black ancestry showing the lowest proportion of KIV-2B individuals, which is consistent with previous findings [2, 26, 28] (Additional file 1: Table S12).

When excluding the PSVs [8] that defines the KIV-2B subtype in exonic and intronic sequences [2], our approach detected a total number of 707 unique mutations across all samples, including 256 missense mutations, 37 nonsense, and 8 splice-site mutations. Ninety-five intronic mutations were located within 25 bp around the exons (referred to as “splicing region”) and could affect intronic core splicing elements [15] (Table 4 and Additional file 6: Fig. S2).

**Table 4:** Unique *LPA* KIV-2 mutations within the target regions (KIV-2 exons 1 and 2 ± 100 bp) in each population. KIV-2 mutations are divided by mutation type for UK Biobank individuals (*n* = 199,119). Since the signature-based approach was developed and benchmarked in individuals of European ancestry, summary data are here filtered for White (*n* = 186,607), South Asian (*n* = 3465), and Black (*n* = 3200) ancestry. The PSVs defining the KIV-2B were not considered as mutations. The location “splicing region” was defined as within the 25 bp upstream and downstream KIV-2 exons

	**All ancestries (*n* = 199,119)**	**White (*n* = 186,607)**	**South Asian (*n* = 3465)**	**Black (*n* = 3200)**
**KIV-2 mutations in target regions**	707	692	283	294
**Missense mutations**	256	248	103	123
**Nonsense mutations**	37	35	12	10
**Splicing region mutations**	95	93	36	26
**Splice-site mutations**	8	8	3	1

Our signature-based approach reported similar variant levels for all three KIV-2B canonical variants in the UKB cohort (Additional file 1: Table S13 and Additional file 6: Fig. S3), suggesting that the KIV-2B haplotype was correctly identified and that the results should be consistent with previous findings on KIV-2 variation [2].

**Fig. 8:**
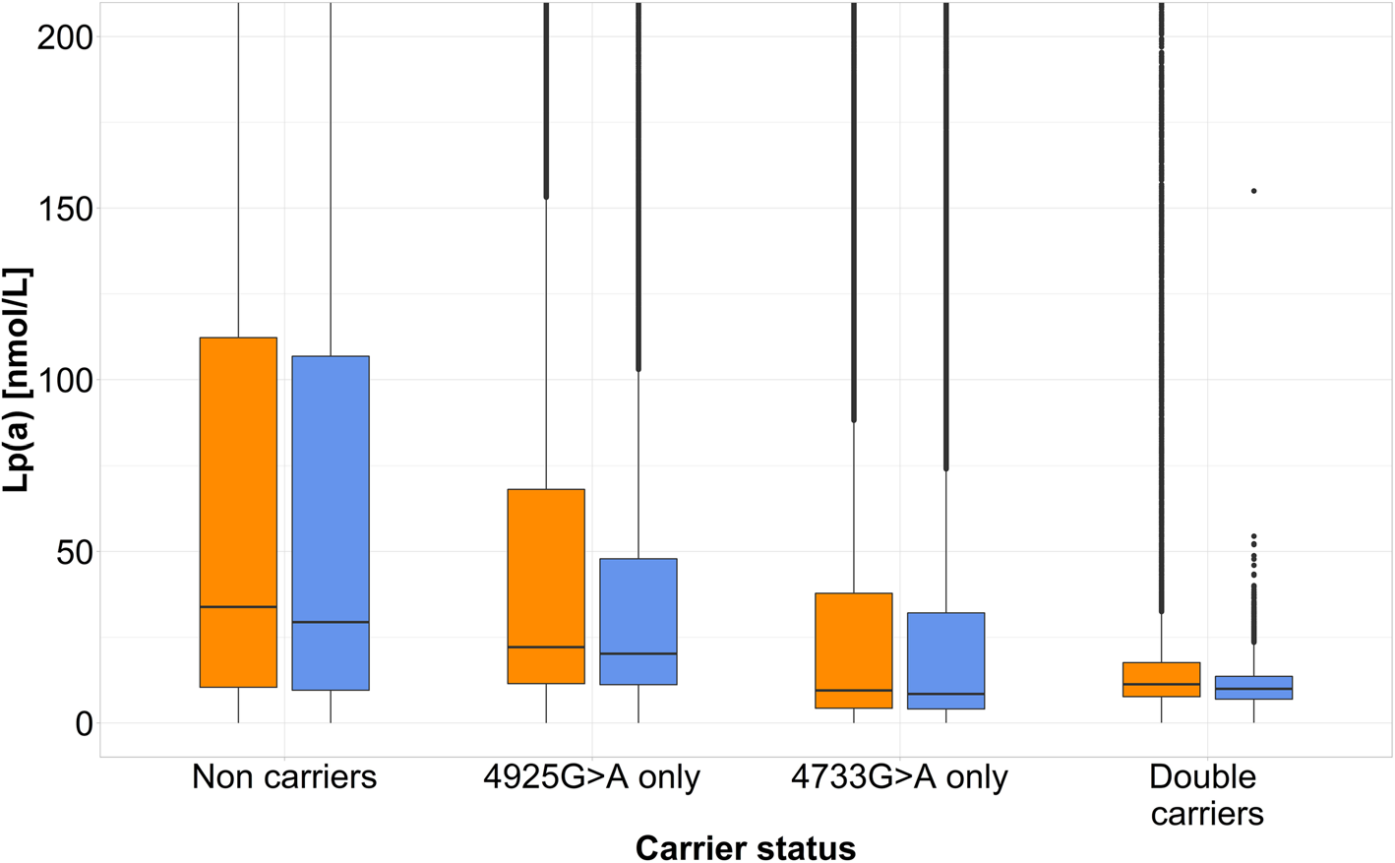
Distribution of the Lp(a) concentration (median [interquartile range]) in *n* = 166,982 UKB individuals of White ancestry with available Lp(a) concentrations and carrier status for the genotyped SNPs rs75692336 and rs6938647 and the WES called SNPs 4925G > A and 4733G > A. UKB individuals were classified into 4 groups based on the carrier status for **A** the rs75692336 and rs6938647 SNPs (tagging SNPs for the KIV-2 SNPs 4925G > A and 4733G > A, represented in orange) and **B** KIV-2 SNPs 4925G > A and 4733G > A called directly from WES data (blue). The *y*-axis is limited to Lp(a) concentration up to 200 nmol/L

The carrier frequency in UKB for the two currently strongest Lp(a)-lowering KIV-2 SNPs (KIV-2 4733G > A [15] and 4925G > A [12]) was 27.2% for 4733G > A [8, 15] and 17.0% for 4925G > A [8, 12]. Both frequencies were higher in White individuals [12, 15] (4733G > A: 28.4%, 4925G > A: 17.5%) and lower in UKB participants with black ancestry (4733 G > A: 2.7%, 4925G > A: 2.2%; Additional file 1: Table S14), as reported previously [12, 15]. In earlier work, we had identified tagging SNPs for KIV-2 4733G > A [15] and 4925G > A [12] and used them to investigate the effect of 4733 G > A and 4925G > A in the UKB cohort. We now stratified the UKB cohort into the same 4 groups as done with the proxy SNPs but determining 4733 G > A and 4925G > A directly (Fig. 8). The median group Lp(a) concentration was similar but by using the directly called SNPs the interquartile range was considerably narrower. This indicates a higher specificity of the direct variant calls compared to the use of the imperfect proxy SNPs. This shows that direct access to the KIV-2 variability can further improve and refine epidemiological studies on Lp(a).

Additionally, we successfully detected also three other Lp(a)-lowering KIV-2 mutations (KIV-2.1 + 1G > A, KIV-2.1 Y51D and KIV-2.1 + 0C > T) that had been identified recently in WES data of ≈50,000 UK Biobank participants (UKB 50 K WES release) using a different strategy to isolate KIV-2 reads [8]. Our signature-based approach successfully detected these three mutations in 199,119 individuals with carrier frequency of ≈1%, ≈0.6%, and 0.1%. Each SNP was significantly associated with reduced Lp(a) concentrations (*β* =  − 12.7 nmol/L, − 6.8 nmol/L, and − 12.0 nmol/L, respectively; all *p* < 2e − 16, Additional file 1: Table S15). This is in accordance with Mukamel et al. [8].

**Fig. 9:**
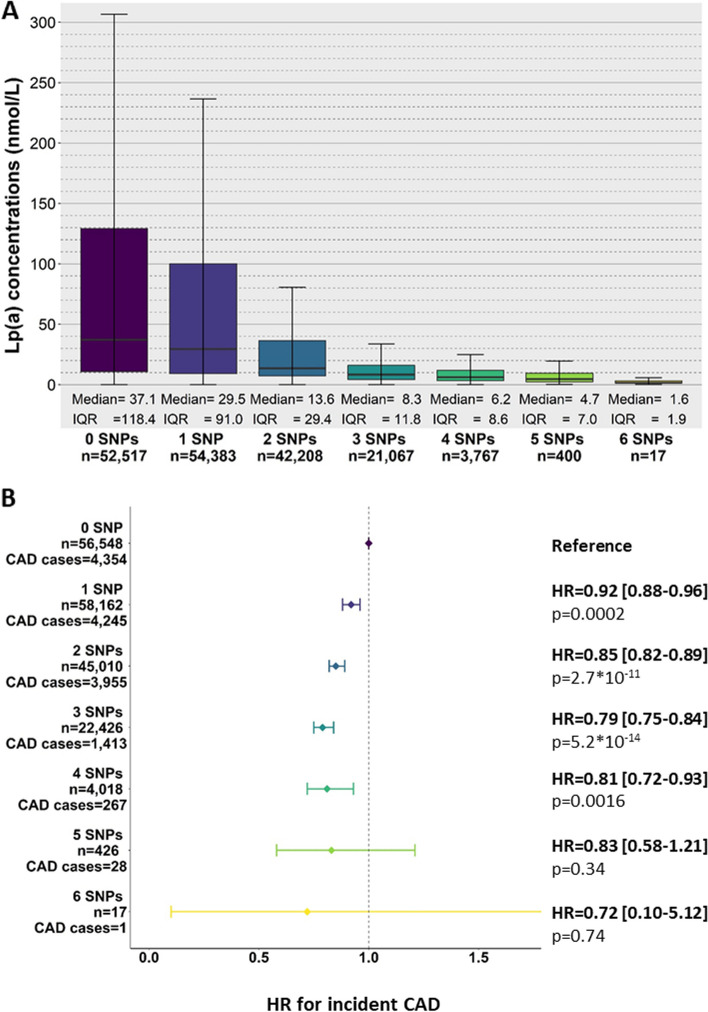
Impact of 9 Lp(a)-lowering SNPs identified within the KIV-2 VNTR on Lp(a) concentrations and CAD risk in 186,607 UK Biobank participants of White ancestry with available WES data. **A** Distribution of the Lp(a) concentration in individuals with 0 to 6 of the Lp(a)-lowering SNPs. The Lp(a) concentration was available for 174,359 individuals. **B** Hazard ratio (HR) for the risk for coronary artery disease (CAD) associated with individuals carrying 0 to 6 of the Lp(a)-lowering SNPs. Total CAD cases = 13,363

We identified 9 SNPs significantly associated with decreased Lp(a) concentration (*p*-value < 5 × 10^−8^) in UKB participants of White ancestry with carrier frequency > 0.015. These SNPs included the previously identified R21X [14] (position 640), 4733G > A [15] and 4925G > A [12], and 6 further SNPs across the KIV-2 VNTR (positions 515A > G, 516A > G, 584C > T, 4672A > G, 4698C > A, 5013G > T in this reference sequence [2, 26]). Median Lp(a) concentrations decreased with increasing number of effect alleles from 37.1 nmol/L (IQR = 118.4 nmol/L; *n* = 52,517) in the reference group (only reference alleles) to 4.7 nmol/L (8.6 nmol/L) in carriers of 5 Lp(a)-lowering alleles (*n* = 400) and even 1.6 nmol/L (IQR = 1.9 nmol/L) in 17 carriers of 6 Lp(a)-lowering alleles (Fig. 9A). These SNPs also showed a rather additive protective effect against CAD. Compared to non-carriers (*n* = 56,548, CAD cases = 4354), the hazard ratio (HR) for incident coronary artery disease (CAD) progressively decreases with increasing number of Lp(a)-lowering alleles. Four Lp(a)-lowering SNPs (*n* = 3767, CAD cases = 1413), decreased the CAD risk by ≈19% compared to non-carriers (HR = 0.81, 95% confidence interval (CI) = 0.72–0.93], *p*-value = 0.0016) (Fig. 9B).

## Discussion

The development of NGS technologies has provided a huge leap forward to genetic research and, despite the advent of long-read third-generation sequencing technologies, NGS is still the workhorse for most population-scale sequencing projects. However, ≈20% of all genes (*n* ≈3800) contain segments that are not resolvable by conventional NGS data analysis approaches [3], with some genes being even completely inaccessible [3, 5–7, 29]. In this paper, we focused on NGS downstream analysis strategies to rescue information from short-read sequencing data for VNTRs, which are dark-by-mapping in public databases [3]. Especially protein-coding VNTRs located in genes implicated in diseases still hide medically important information. We developed an approach to detect variants in VNTRs from short-read sequencing data and provide a scalable and parallelized Nextflow pipeline to the community. To develop our variant calling approach, we used the large KIV-2 VNTR in the *LPA* gene, as the extensive homologies with neighboring kringles as well as other homologous genes [2] makes it particularly challenging and therefore an ideal candidate for set up and benchmarking. Indeed, due to its peculiar structure, *LPA* was even excluded from the latest benchmarking datasets for variant calling in difficult regions [4, 7], also precluding the use of the vast amounts of publicly available WGS and WES data with rich phenotype data for Lp(a) research [30, 31].

We developed a computational pipeline for targeted VNTR variant calling, optimizing and evaluating it using the particularly complex KIV-2 VNTR in the *LPA* gene and applied it to five further medically relevant protein-coding VNTRs located in the genes *FLG*,* NEB*,* UBC*,* DMBT1*, and *SPDYE3*, supporting portability to other VNTRs with unit larger than the read length. Among the variants detected in the KIV-2 WGS data from 1kGP, we recognized biologically functional variants like the Lp(a)-lowering splice-site mutation 4925G > A [12] and the 4733G > A, located in the splicing region of the *LPA* gene [15], as well as three other recently identified KIV-2 variants [8]. In another recent study, we compared the *LPA* KIV-2 variant levels quantified with our pipeline to highly precise Nanopore Sequencing data with error-correction by unique molecular identifiers (UMI-ONT-Seq), finding a very high correlation between the variant levels (*R*^2^ = 0.983) [32]. Similarly, in all analyzed VNTRs, we identified and annotated potentially functional variants like nonsense and splice-site mutations (Table 3).

Our approach extracts the reads of the target VNTR from WGS/WES data already aligned to the human genome reference sequence using coordinates specified within the workflow and can be therefore applied to different genes. We show that identifying paralogous sequence variants (PSVs) allows pinpointing the positions where reference-based artifacts are likely to result in false positive calls (Methods). In some genes (e.g., *LPA*, *DMBT1*, and *UBC*) the VNTR flanking regions resemble degenerated repeats, which are rich in PSVs. This suggests that using stringent coordinates for read extraction, limited to the actual VNTR start and end positions, is needed to improve variant calling accuracy, which puts additional challenges to VNTR-finding algorithms.

We tested variant calling accuracy using alternative ROIs for KIV-2 VNTR reads in 8 gold-standard samples with known KIV-2 variant patterns [2]. As shown in our experiments, approaches that align all exome reads or all *LPA* reads directly to the KIV-2 are not accurate, since also reads from other kringles align to the repeat and produce many spurious variants. Thus, it is necessary to first align all reads to the genome to allow alignment of the confounding kringles and then extract only the KIV-2 reads from the BAM file. Here, we tested three published regions that have been used to isolate KIV-2 reads from WES data already aligned to the reference genome (ROI-2 [3], ROI-3 [8], and ROI-8 [2, 12]). ROI-8 extracted reads from the bona fide start and end VNTR coordinates and was the best performing strategy overall, with sensitivity and specificity comparable to the previously published ROI-3 (Additional file 1: Table S4) but with less false positive calls (ROI-3: from 8 to 20 in experiment 1 and 2; ROI-8 from 0 to 12 in experiment 1 and from 0 to 11 in experiment 2; Additional file 1: Table S4) and therefore higher mean F1-score (dataset A experiment-1 and experiment-2: 81.9%, and 84.6%). This supported the superiority of stringent KIV-2 VNTR coordinates for read extraction.

Another aspect was important in the *LPA* KIV-2 VNTR. Although ~ 20% of Europeans do not carry KIV-2B subtypes, the human genome reference sequences hg19 and hg38 contain only one KIV-2B unit, which thus appears as fixed and single-copy to previously used VNTR-finding algorithms [3, 8, 33]. We observed that a ROI with or without the single reference KIV-2B unit performed best (ROI-8 and ROI-9), depending on the presence of KIV-2B units or not in the analyzed sample. We assume this is because of the following alignment artifact: if an individual does not present KIV-2B units in its genome but its data is aligned to the human reference sequence, which does contain a KIV-2B unit, reads from the nearly identical KIV-3 unit are pulled to the KIV-2B sequence. Subsequently, these reads are extracted together with the bona fide KIV-2 reads, if a ROI also spanning the KIV-2B unit is used. Vice versa, if an individual does present KIV-2B units, these reads are missed when using a ROI without the KIV-2B element for read extraction.

PSVs have been recently used for separation of duplicated sequences before assembling [34]. Similarly, we identified a variant in KIV-3 (reported as rs113727842) that predicts the presence/absence of KIV-2 subtypes and used it to integrate a dynamic ROI assignment in the signature-based approach developed for *LPA*. This minimizes the rate of false positive in non-KIV-2B samples and further increased the performance to mean F1 > 90% in all WES datasets tested (dataset A experiment-1 and experiment-2 and dataset B: 95.7%, 95.1%, and 90.8%), paving the way to reliable KIV-2 variant annotation at scale. We tested this in UKB and found a set of nearly 300 high-confidence variants affecting protein sequence (including 37 nonsense and 8 splice site mutations), including multiple previous findings [8], which further supports to the validity of our variant calls. This also revealed new associations between SNPs hidden in the KIV-2 and reduced Lp(a) concentrations. We provide the so far largest dataset on KIV-2 variants set as a return result to UKB.

Several methods exist to estimate the repeat length as well as mutations within VNTRs from short-read sequencing data. The computational approach presented in [35] is based on hidden Markov models and is specifically focused on repeat unit count estimation. This approach has been further extended in [36] to identify insertions and deletions as well as small frameshifts within VNTRs. Work presented in [37] utilized a repeat-pangenome graph (RPGG) based on haplotype-resolved assemblies, which allowed the detection of variation in both VNTR length and repeat composition. The work in [38] presents a method to discover genetic variations in duplicated genome regions by applying a masking approach to the reference genome. Unlike these approaches, our approach allows the direct detection of intra-repeat mutations, also known as batch sequencing in the field of *LPA* genetics [39]. This strategy has been originally utilized to amplify and sequence repeats as amplicon mixtures and detect variants as somatic mutations by aligning all data to one single repeat. Ebbert et al. conceived independently a similar approach and described for the first time how such an approach can recover variants within unmappable regions (denoted as camouflaged regions) in a genome-wide manner using WGS and WES data [3]. Additionally, an algorithm has been outlined to rescue a rare ten-nucleotide frameshift deletion in the CR1 gene [3]. Building on this work and VNTR coordinates determined in [8], we are able to present a computational pipeline targeting the recovery of SNVs from coding VNTRs. We especially focused on the complex *LPA* KIV-2 region. Our approach implements a dynamic adaptation of the read extraction steps leveraging a SNP in the flanking region of the VNTR, which predicts the presence of KIV-2B units. We show that this significantly improves variant calling even more, unlocking access to this medically relevant region and showing the potential for other VNTRs. Representing VNTRs in the widely used variant calling format (VCF) poses an ongoing challenge. Future work requires either the development of a new file format or the enhancement of the VCF file format capable of accurately representing VNTRs, in addition to the creation of novel benchmarking tools for VNTRs.

In summary, we provide our computational workflow as a Nextflow pipeline that can be used for mutation screening in dark VNTR regions. It provides a framework to detect, evaluate, or even benchmark different read extraction strategies on thousands of samples with high specificity and sensitivity. We applied it without any changes to call and annotate variants in the WGS data of 6 VNTRs. Additionally, the signature-based approach specifically developed for *LPA* represents a blueprint for researchers to incorporate VNTR-specific information (e.g., repeats subtypes) and further minimize the false positive calls originating from reference-based artifacts. Nevertheless, also our generic computational workflow (i.e., without the signature-based approach) already detected KIV-2 variants that have been investigated in previous work, with some of the largest effects on Lp(a) concentrations (4925G > A and 4733G > A). As our results show, promising variants with a possible biological function have been discovered for the other analyzed VNTRs.

### Limitations

Our approach also has limitations. First, while our approach works for coding VNTR that are longer than the read length, our pipeline only reports the relative position of mutations within the repeat. Second, the current version of the pipeline is currently limited to SNV detection. Nevertheless, the underlying architecture allows us to expand the approach also to the detection of insertions and deletions in the next pipeline versions. Given the modularity of the Nextflow pipeline, this can be implemented also by any other user with suitable variant callers at hand. Third, the *LPA* KIV-2 sample set used for setting up the analysis concept consisted of individuals of European ancestry. This was dictated by sample availability at our Institute and by the wish to use samples with known KIV-2 numbers (determined with Western blot as described elsewhere [40]). Importantly, this limitation applies only to the use of the KIV-2B signature sequence, which could be designed only for individuals of European ancestry. The other parts of the optimal ROI selection, which already considerably boosted calling accuracy, are ancestry independent and allowed analyzing five other medically relevant VNTRs, currently limited to a similar repeat structure as *LPA* (i.e., VNTR unit larger than the read length). We acknowledge that mutations present only in one or a few individuals in the population data might also be technical errors. This may most likely apply to *LPA*, which has the highest number of repeats and thus requires calling variants down to 1.25% fractional representations.

## Conclusion

We provide a scalable and highly parallelized Nextflow pipeline to detect variants in VNTRs from short-read sequencing data. Leveraging our work on *LPA*, we evaluated and benchmarked VNTR variant detection in detail on this gene and developed a novel approach to reliably extract variants from short-read data. We also analyzed five other medically relevant VNTRs. Similar to *LPA*, the tested VNTRs all include a repeat unit length larger than the read length. Our workflow can be applied to any NGS data of other VNTRs, thereby unlocking variant calling at scale. For *LPA*, we further optimized the workflow and developed a signature-based approach to account for sequences originating from highly homologous neighboring exons. We show that even in special cases like the *LPA* gene with additional intragenic homologies to the VNTR, a detailed investigation can further improve the result and PSVs can be leveraged to discriminate the origin of sequencing reads, minimizing possible false positives. While the signature-based approach was developed in this paper only for the *LPA* gene, we show that our general approach already detected important variants in *LPA* located in positions not affected by PSVs. Therefore, our framework will unlock the study of VNTRs base variation at scale in a reproducible and scalable manner.

## Methods

### Genomic samples and datasets

All experiments for setup and benchmarking of our VNTR variant calling pipeline were performed on 24 samples with known KIV-2 variant patterns drawn from Central-European individuals previously screened for mutations in the KIV-2 region by ultra-deep targeted sequencing [2]. In brief, the experiment consisted of 3 main steps and was conducted on 123 Central-European individuals. First, a single primer pair was used to selectively amplify all KIV-2 units as a mixture, leveraging their high sequence homology (“batch amplification approach”) [26]. Second, the resulting amplicon mixture subjected to NGS and the KIV-2 reads were mapped to one KIV-2 repeat as a reference. Since KIV-2 variants can be present in one or few repeats, only a subset of reads will present the respective mutation. Thus, in the third step, a variant calling tool suitable for low-level mutations was used to detect mutations down to 1% mutation level [2]. Among the samples in dataset A and B, 16 samples did not present KIV-2B units (non-KIV-2B samples).

For the KIV-2B signature sequence experiments (see below), the sample set above has been complemented with a further 42 samples from the Austrian healthy worker study [41] SAPHIR that had undergone amplicon-based targeted KIV-2 deep sequencing during the same previous project [2, 12]. Details about the SAPHIR study are given in Additional file 8. Measurements of the Lp(a) concentration and KIV domains number for all 66 samples were performed at the Institute of Genetic Epidemiology, Medical University of Innsbruck, Austria, following the ELISA and Western Blot methods detailed elsewhere [40].

### DNA sequencing

We generated WES data for samples with known KIV-2 variants divided into two independent working datasets (dataset A: 8 samples, dataset B: 16 samples, Fig. 4). In dataset A, we performed two independent sequencing experiments (experiment-1 and experiment-2) to generate WES data using two different exon enrichment chemistries (Agilent SureSelect Human Al Exon v6 kit and Agilent SureSelect Human Al Exon v8 kit). Dataset A experiment-1 was performed at BGI (Hong Kong, China) on an Illumina HiSeq system X Ten using a 2 × 150-bp paired-end read chemistry. Dataset A experiment-2 was performed at Eurofins Genomics (Konstanz, Germany) using a 2 × 150-bp sequencing mode on an Illumina NovaSeq 6000 system. In experiment-2, also the 16 samples from dataset B were sequenced.

WES data provided by BGI (dataset A experiment-1) were mapped to the hg19 reference genome using the Burrows-Wheeler Aligner (BWA) software, while Eurofins used the commercial Sentieon pipeline to map the data to the reference genome. We remapped all WES data provided by Eurofins with the same command as used by BGI (Results and Additional file 2).

To identify the KIV-2B signature sequence, we selected 66 samples from the KIV-2 variability dataset [2], including the WES-sequenced samples from datasets A and B. The KIV-3 repeat was Sanger-sequenced to identify sequence differences between the first exon of KIV-3 and KIV-2B (Additional file 1: Table S16).

The KIV-2 positions are numbered as in [2]. The KIV-2B canonical variants at positions 14, 41, and 86 in exon 1 correspond to the positions 521,594 and 666 in this reference sequence [2, 26].

### Public datasets and statistical analyses

We retrieved high-coverage WGS of the original 2504 unrelated samples of the 1kGP from 26 populations [16]. In the last release, samples were sequenced to a targeted depth of 30X genome coverage with Illumina NovaSeq 6000 and aligned to GRCh38 [16]. We investigated 6 protein-coding VNTRs based on start and end coordinates estimated by Mukamel and colleagues [8] (Additional file 1: Table S17). For *LPA* KIV-2 VNTR, we aligned the reads to the reference sequence from [2].

The UK Biobank database includes health-related information and in-depth genetic data for more than 500,000 individuals recruited between 2006 and 2010 (Additional file 1: Table S11). We defined 3 ancestry subgroups based on entries in data field 21,000 (White ancestry: “British”, “Irish” and “Any other White Background”; South Asians: “Indian”, Pakistani” and “Bangladeshi”; Black: “Caribbean”, “African” and “Any other Black background”). Serum Lp(a) concentrations were measured at baseline using an immunoturbidometric assay employing the Denka Seiken method (Additional file 2). In this work, we accessed WES data for 200,000 UKB participants released by October 2020. As described in [42], exome enrichment was performed with the IDT xGen Exome Research Panel v1.0 and a dual-indexed 75 × 75 bp paired-end reads chemistry was used for sequencing on an Illumina NovaSeq 6000 platform. We investigated the effect of the KIV-2 variants at position 730 (KIV-2.1 Y51D), 740 (KIV-2.1 + 0C > T and 741 (KIV-2.1 + 1G > A) using quantile regression models adjusted for age and sex. For all detected KIV-2 SNPs, we tested the impact on Lp(a) concentration with linear regression in UKB individuals of White ancestry with available Lp(a) concentrations. We used Cox regression analysis (R package survival) with age as time scale and adjusted for sex to investigate the risk for incident CAD associated with the increasing number of Lp(a)-lowering SNPs (carrier frequency > 0.015) per individual. CAD events were defined using ICD10-codes I21–I25, as previously done [15, 43]. In the study at hand, all UKB analyses were performed with R (version 3.6.3).

### Nextflow pipeline for variant calling in VNTRs

We implemented our variant calling approach for short-read VNTRs data in a parallelized workflow (Fig. 3) built in Nextflow using DSL2. The pipeline is publicly available at https://github.com/genepi/vntr-calling-nf. Nextflow is a prominent workflow manager that allows writing scalable and reproducible pipelines using software containers. Our pipeline requires as an input-aligned files in BAM format. The user specifies (a) the ROI for read extraction providing coordinates (e.g., as BED file) and (b) the sequence of the single repeat unit to be used as reference sequence. The workflow then extracts the reads from the input file using the ROI, converts them into FASTQ files, and remaps the extracted reads to the single repeat sequence. Until this step, all samples are analyzed in parallel. We then call variants (Fig. 3 step 5) using the mutserve variant-caller for all samples at once, which discriminates low-level mutations from sequencing errors using a maximum likelihood mode to take sequencing errors per base into account [24]. We optimized settings for low-level variant detection (Additional file 1: Table S18). The final output is a customizable annotated variant file in tab-delimited text format [2] and is also available in VCF format.

We applied the pipeline to 2504 samples from 1kGP for 5 other protein-coding VNTRs with VNTR units that are longer than the read length and include less than 50% PSV positions. The VNTRs were selected from the list provided in [8], using the VNTR repeat unit consensus sequence defined in [8] as a reference sequence for variant calling.

### Paralogous sequence variant identification

Locations showing PSVs caused by inter-repeat unit differences were identified by extracting for each VNTR region (as defined by the VNTR coordinates provided in [8]) all repeat units and generating a multiple sequence alignment using Clustal Omega. VNTR unit boundaries were identified by aligning the repeat unit consensus sequence provided in [8] to hg38 using BLAT and further curating the results manually. All positions showing inter-repeat unit differences were then determined by position-like comparisons of all available repeat units (Additional file 1: Table S19).

### Improved pipeline for the *LPA* KIV-2 VNTR

For the *LPA* KIV-2 VNTR, we developed a signature-based KIV-2 variant calling approach and integrated it as step 1 into a Nextflow pipeline specific for this region (Fig. 6B). In a first step, we extract the complete *LPA* region from the BAM file and apply our identified signature sequence to discriminate between KIV-2B and non-KIV-2B samples (step 1). Depending on the type, we then extract reads using the corresponding optimal coordinates (ROI) (step 2) and convert the aligned reads to unaligned reads in FASTQ format (step 3). The reads are remapped to the sixth KIV-2 reference repeat [2] (step 4) for variant calling (step 5). Until step 4, all samples are analyzed in parallel.

We applied the pipeline to 199,119 samples from UK Biobank on our in-house HPC. Due to its flexibility, the pipeline can also be executed directly at the Research Analytics Platform (RAP) from UK Biobank or any other cloud provider. The pipeline and documentation are available at https://github.com/genepi/vntr-calling-nf.

### Variant calling evaluation

For evaluation purposes, we compared our variant calling method mutserve [2, 24] with Mutect2 [44] on dataset A (Additional file 2). Both resulted in accurate and comparable results, showing a high F1 for both experiments. Mutserve produced a slightly higher F1 (95.2–95.7%) compared to Mutect2 (82.3–84.9%) and we therefore integrated mutserve as the default variant caller in our workflow (Additional file 1: Table S20).

### Initial reads alignment to the genome reference sequence affects variant detection

For dataset A, the underlying computational pipeline for initial read alignment (executed directly by the sequencing provider) differed between experiment-1 and experiment-2 (Methods). For experiment-2, we noticed a considerably lower F1 value than for experiment-1 when applying our approach (71.6% vs 95.7%, Additional file 1: Table S21 and S8). Thus, we realigned the WES data from experiment-2 using the same alignment command used for experiment-1. When using the remapped BAM files as an input for our signature-based KIV-2 approach, the F1 in experiment-2 improved from 71.6 to 95.1%. Accordingly, we also remapped the WES data of dataset B, which had been generated and aligned using the same alignment strategy as dataset A experiment 2.

### Supplementary Information

Additional file 1. Supplementary tables.

Additional file 2. Supplementary methods.

Additional file 3. Supplementary Note for Sanger sequencing.

Additional file 4. Supplementary note for LD with KIV-2 SNPs.

Additional file 5. Sequences alignments.

Additional file 6. Supplementary figures.

Additional file 7. Nextflow workflow report.

Additional file 8. Supplementary note for Sanger Sequencing population.

Additional file 9. Peer review history.

## Data Availability

Our computational workflow is published under the MIT license and is freely available at https://github.com/genepi/vntr-calling-nf and Zenodo [45]. The 1000 Genomes Project datasets (*n* = 2504) from the *LPA* region (Exome and WGS, BAM format) are also available at Zenodo [46]. The UK Biobank analysis has been conducted under application 62,905, BAM sequence files are only available to approved researchers directly from the UK Biobank. The SAPHIR dataset (*n* = 24) for the initial pipeline setup is not publicly available due to restrictions in the informed consent. However, they are available from the corresponding author on reasonable request.
