# Supplementary material for: Resolving intra-repeat variation in medically relevant VNTRs from short-read sequencing data using the cardiovascular risk gene LPA as a model
Source: Genome Biol. 2024 Jun 26;25:167. doi: 10.1186/s13059-024-03316-5 (PMC11201333; doi:10.1186/s13059-024-03316-5)
Supplement: Supplementary file 2 — Additional file 2. Supplementary methods. [file 13059_2024_3316_MOESM2_ESM.pdf]

Additional File 2

Supplementary Methods

Lp(a) measurements in UK Biobank

In UK Biobank, serum Lp(a) concentrations were measured at recruitment time with an immunoturbidometric assay (Randox Laboratories; Crumlin, County Antrim, United Kingdom using a Beckman Coulter AU5800 Platform). Among the 199,119 individuals with available WES data, 185,988 had available Lp(a) measurements and those below and above the assay´s reportable range (3.8-189 nmol/L) were 18,857 (10%) and 13,131 (7%) individuals, respectively. These values were requested separately. Sample with concentrations above the analytical range were reanalyzed after serial dilutions as described in https://biobank.ndph.ox.ac.uk/showcase/showcase/docs/serum_biochemistry.pdf.

Identification of the KIV-2B signature sequence

We used Sanger Sequencing to analyze the KIV-3 repeat of 66 samples with known KIV-2B carrier status (Coassin, Schönherr et al, J Lipid Res, 2019). Thereof, 50 were KIV-2B individuals (74.2%) and all of them carried at least one T-signature at position 86 in KIV-3 exon 1 (see Figure below and Additional File 1- Supp. Table 5). Also 11 out of 16 individuals without KIV-2B units were correctly identified by the absence of this T (CC genotype) (see Additional File 1- Supp. Table 5). These data brought focus to a C at position 86 in KIV-3 exon 1 and suggested a correlation between a T at KIV-3 exon 1 position 86 and the KIV-2B haplotype identified by the canonical variants at position 14, 41 and 86 (see Figure 1). Among the 5 non KIV-2B individuals with the T, only one was T-homozygous (see Additional File 1- Supp. Table 5).

We therefore defined a KIV-2B signature sequence (sequence CCACTGTCAC**T**GGAA; T allele at position 86 in bold) that encompasses the T at KIV-3 exon 1 position 86 and can be detected in the raw format of whole-exome sequencing (WES) data.

As expected, non KIV-2B individuals with CC genotype at the signature position (Additional File 1- Supp. Table 6) had almost no WES reads carrying the KIV-2B signature sequence in WES data of both dataset A and B (Additional File 1- Supp. Table 6). Similarly, KIV-2B samples that were T-homozygous at the signature position were prone to have a higher number of WES reads carrying the signature sequence

compared to KIV-2B samples that had the signature-T only on one allele (Additional File 1- Supp. Table 6).

The discriminative threshold was calculated as the mean read coverage of the signature sequence plus 2 standard deviations (Additional File 1- Supp. Table 6), using the available WES data from the non KIV-2B samples (n=12). It correctly classified all 12 KIV-2B individuals with available WES data and only one non KIV-2B individual across all samples sets was wrongly classified as KIV-2B (Additional File 1- Supp. Table 6).

Considering that non-KIV-2B carriers represent ~20% of Europeans individuals (Coassin, Schönherr et al, J Lipid Res, 2019), we estimate that about 6% of European individuals will carry a T at position 86 of exon 1 in KIV-3 despite being non KIV-2B individuals. In WES data, our signature sequence approach further reduced the misclassification to ~4%:

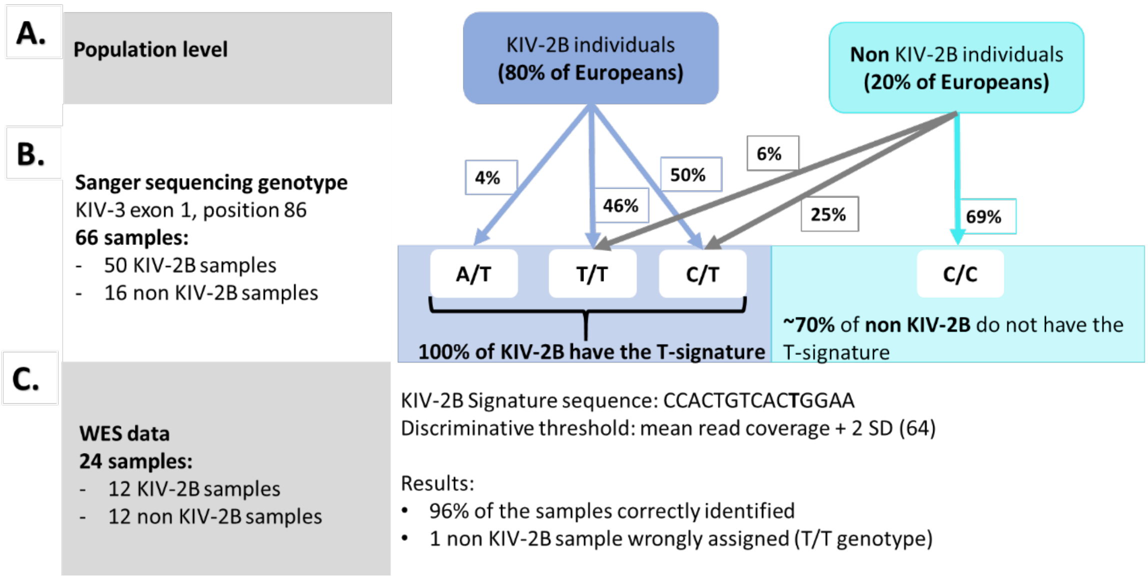

Workflow for the identification and application of the KIV-2B signature sequence. ***Panel A)*** *At population level, ~80% of the individuals are KIV-2B individuals (Coassin, Schönherr et al, J Lipid Res, 2019).* ***Panel B)*** *In 66 samples analyzed using Sanger Sequencing in KIV-3, all KIV-2B individuals carry the T-signature at position 86 in KIV-3 exon 1. Conversely, ~70% of the non KIV-2B individuals are homozygous for C at this position. This would translate to only ≈6% of the individuals in European population being misclassified by the T-signature approach.* ***Panel C)*** *In our whole-exome sequencing (WES) data (n=24, 12 KIV-2B samples and 12 non KIV-2B samples), only one non KIV-2B was wrongly classified as KIV-2B sample. SD=standard deviation.*

Mapping of the WES data to the reference genome

We generated WES data at both BGI (Hong Kong, China) and Eurofins Genomics (Konstanz, Germany) using kit v6 and kit v8, respectively, of the Agilent SureSelect Human Al Exon chemistry for exon enrichment.

At BGI provider, the GATK best practices guide has been used for alignment and variant calling. The alignment to the human reference genome was performed using Burrows-Wheeler Aligner (BWA v0.7.15). Our computational workflow uses the provided BAM files by BGI as an input (WES Report 2019/7/19).

At Eurofins , inhouse analysis workflow v1.1.0 has been used for alignment and variant calling. The alignment to the human reference genome was performed using Burrows-Wheeler Aligner (BWA v0.7.17) running through the Sentieon framework (https://www.sentieon.com/). When evaluating dataset A, a substantially decrease in F1-score has been noticed for this method. We therefore used the same BWA mapping command as for the BGI data. We remapped data provided by Eurofins using the following command:

```
bwa mem -M -R 'read_group_tag' hg19.fasta read1.fq.gz read2.fq.gz > aligned_reads. SAM
```

Mutect2

Mutect2 has been executed using GATK v4.2.6.1. The input samples to Mutect2 were realigned to KIV-2 using the best identified read extraction strategy (ROI-8 vs ROI-9). Therefore, the input was identical as for mutserve variant caller.

For each sample, the following two commands have been executed:

```
gatk-4.2.6.1/gatk Mutect2 -R kiv_2.fasta -L KIV2_6 --min-base-quality-score 30 -I <bam_file> -O <out.vcf>
```

```
gatk-4.2.6.1/gatk FilterMutectCalls -R kiv_2.fasta -V <out.vcf> -O <final_out.vcf>
```

MapLocus

We use customizable tab-separated files (internally named MapLocus file) to annotate genetic features within the VNTRs. The files use coordinates referring to the VNTR reference sequence used for variant calling to map freely definable annotations to the sequence. If a feature is protein-coding, also the translation frame can be defined, allowing automatic determination of amino acid changes with our annotation script (see https://github.com/genepi/vntr-calling-nf/tree/main/paper_analysis).

The annotations for all VNTRs analyzed in the present manuscript except LPA, were defined by mapping the VNTR repeat unit consensus sequences defined by Mukamel et al., Science, 2021 (Supplementary Table 17) to hg38 using BLAST. The annotation of the LPA KIV-2 was available from previous projects (Coassin, Schönherr et al, J Lipid Res, 2019 and Coassin et al., Eur Heart J, 2017) For single exon VNTRs, the genome coordinates of the best hit (respectively the first hit in translation direction, if multiple or

all repeats had the same BLAST quality values,like FLG), were looked up the UCSC Genome browser hg38 and the translation frame was determined visually (UCSC Gencode v43 data track).

For multi-exon repeats, the UCSC hit was used to determine, which exon numbers were encompassed by the selected VNTR unit and the translation phase of all exons in the VNTR unit was taken from the "Exons" overview in the respective Ensembl v109 transcript data sheet. This was visually confirmed in the UCSC Genome browser. If the human genome reference contained multiple VNTR repeats, we cross-checked that also the exons in the subsequent repeats would have the same translation phase. Additionally, the UCSC Genome browser annotation was used to also add intron regions to the maplocus file.

This manually curated procedure ensures a precise representation of the VNTR unit structure and can be applied in principle to any other VNTR as well, provided that the reference sequence and gene structure annotations are available. However, if the human genome reference sequence missed annotations of the genetic elements within the VNTR, this would affect only the annotation of mutation locations and amino acid effects, not variant calling itself.
