# Supplementary material for: Resolving intra-repeat variation in medically relevant VNTRs from short-read sequencing data using the cardiovascular risk gene LPA as a model
Source: Genome Biol. 2024 Jun 26;25:167. doi: 10.1186/s13059-024-03316-5 (PMC11201333; doi:10.1186/s13059-024-03316-5)
Supplement: Supplementary file 3 — Additional file 3. Supplementary Note for Sanger sequencing. [file 13059_2024_3316_MOESM3_ESM.pdf]

Additional File 3

Supplementary Note

Considerations regarding the specificity of the KIV-3 Sanger Sequencing protocol

Supplementary Note 1: Primer binding sites

The *LPA* KIV-3 and KIV-2 domains are highly similar. Sanger sequencing requires a specific PCR amplicon to ensure that the correct region is sequenced. Due to the high homologies across all KIV domains, some homologies in the binding sites are inevitable, but the primer binding site combination needs to be unique. In the present work, the primers of Noureen et al. PloS One 10, 2015 where used.

The specificity of the amplicon towards KIV-3, excluding KIV-2B units, is provided by the reverse primer, which has a perfect match only downstream of KIV-3 exon 1 (hg38), while the forward primer has a perfect match both upstream of KIV-3 and upstream of KIV-2B (i.e. the third KIV-2 repeat in transcription direction). Additionally, the locus reference sequence was screened for degenerated sequences using BLAT, BLASTn (word size 7) and, additionally, manually, in SnapGene v7 (GSL Biotech LLC, Boston, MA, USA) by searching for the primer sequences across the *LPA* genomic sequence, while allowing for gaps or mismatches at every 4 bases or more (maximum degeneration level allowed). All hits where subjected to a multiple alignment using ClustalO (see below). This confirmed that the only perfect primer combination is found in KIV-3. The reverse primer provides specificity against KIV-2B by three mismatches at its most 3' bases, which prevent extension by the used Qiagen HotStar polymerase, which lacks 3'-5' an exonuclease activities. The setup resembles thus an ARMS-PCR protocol.

Supplementary Note 2: Sanger sequencing traces

The specificity of the PCR amplicon can be inspected also directly in the Sanger traces, where PSVs originating from coamplified kringles would result in mixed Sanger sequencing signals at defined positions, where the kringles differ.

The Sanger traces cover ≥520 bases per sample sequenced in both directions and with high quality (average +/- SD of percentage bases >Q30 = 94.15%+/-0.03; >Q20 = 98.02%+/-0.01; quality values as reported by Sanger analysis software Sequencher 5.4, GeneCodes, Ann Arbor, MI, USA). They cover the complete first exon of the kringle, 191 bases upstream of the exon and 170 bases downstream of the exon (chr6:160611371-160611891 in hg38; all designations for strand orientation like upstream or downstream are given respective to the transcript orientation, which is on the minus strand). Besides the primer design that mechanistically prevents amplification of other kringles with the used polymerase, the specificity of the sequencing is supported also by the following observations:

1. The peaks for heterozygous SNPs in the electropherograms show roughly 50:50 peak heights, which would be unlikely, if we would amplify a mixture of multiple KIV units (especially multiple KIV-2 units, which are most similar to the KIV-3 based on the primer alignments). Exemplary screenshots are shown in Supplementary Note-Figure 1 here below.
2. BLAT (hg38) finds the consensus Sanger sequence only in KIV-3. The KIV-2B unit has 97.4% identity. All other KIV units (KIV-1, KIV-4 to KIV-10) had much lower base identity levels in BLAT. In a multiple sequence alignment of ≈520 bases encompassing the Sanger consensus sequence and all hits in KIV-2B and KIV-2, the Sanger-sequenced region would contain 40 PSVs between KIV-3 and KIV-2 (red bases in Alignment 3 below). 15 PSV are seen also between KIV-3 and KIV-2B (yellow highlights in Alignment 3 below), which is the most similar other KIV unit. Manual inspection of all our electropherograms did not find hints for signals originating from other KIV units than KIV-3. Seven KIV-3/2B differences are clustered in the intron immediately downstream of the exon (chr6:160,611,490-160,611,540 in hg38). These bases are underlined in Alignment 3 below and some exemplary Sanger traces are shown in Supplementary note-Figure1. No signal admixture is seen.
3. Addition of KIV-4 to this evaluation as the next most similar KIV domain (albeit the reverse primer would bind in KIV-4 only with the last 4 mismatched bases at the 3’ end and would thus actually not be extended; see the primer alignments discussed above) would result in about 120 PSVs, including several indels and would thus be readily detectable (Alignment 4 below). Such signals were not observed.

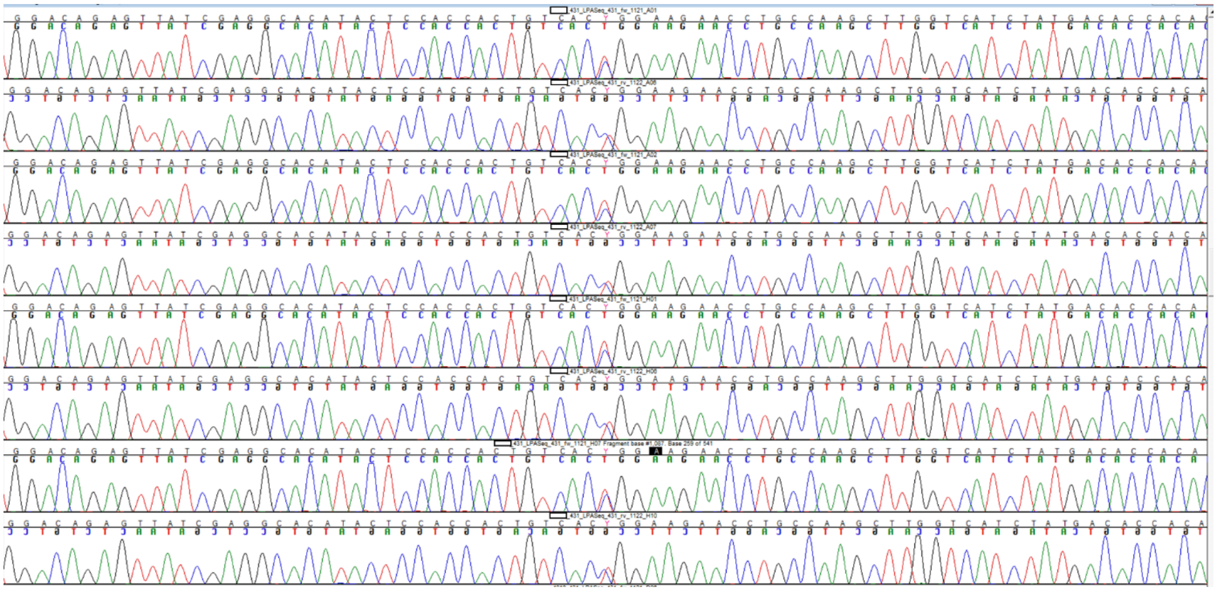

Supplementary Note - Figure 1: *Electropherograms (screenshot from analysis software: Sequencher 5.4, GeneCodes, Ann Arbor, MI, USA) of four representative samples (forward and reverse primer sequences alternating from top to bottom). The base in the C/T SNP om the center is the "signature SNP" positions. It clearly ≈50:50, germline SNP-like signal ratio for the two alleles (overlapping red/blue peaks), supporting that no KIV-2/KIV-2B units are coamplified, as these signals would dilute the signal because they occur multiple times. Signal height zoomed by approximately 50 % to detect also minor admixtures. Sample IDs covered due to data protection reasons.*

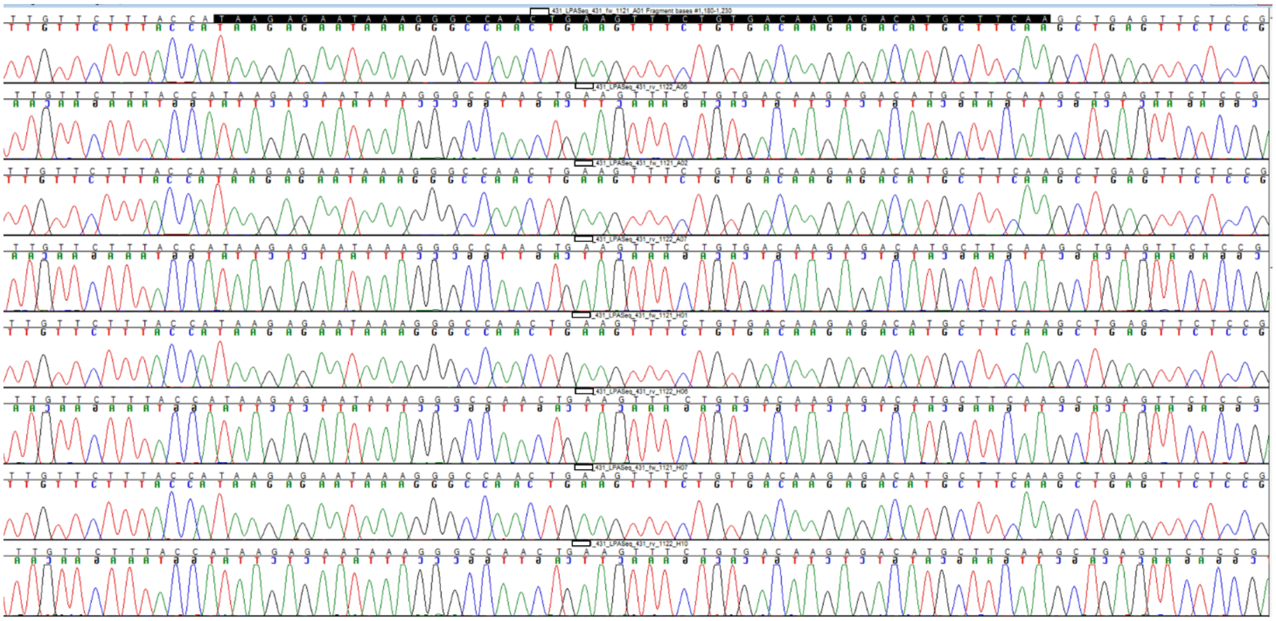

Supplementary Note - Figure 2: *Electropherograms (analysis software: Sequencher 5.4, GeneCodes, Ann Arbor, MI, USA) of four representative samples (forward and reverse primer sequences alternating from top to bottom). The region highlighted in black corresponds to the region underlined in Alignment 3, which contains multiple PSVs that would allow detecting admixture of KIV-2B signals (at levels relevant for Sanger sequencing detection). Signal height zoomed by approximately 50 % to detect also minor admixtures. No signal admixtures are seen. Sample IDs covered due to data protection reasons.*

Alignment 1: CLUSTAL O(1.2.4) multiple sequence alignments for the forward (fw) primer

Differences to the primer are shown in red

```
Primer_fw      GAGCGCACTTTGCAGTGAGAAG 22
KIV-3          gagcgcactttgcagtgagaag 22
KIV-2B-3       gagcgcactttgcagtgagaag 22
KIV-2-6        gagctcactttccaatgaggaa 22
KIV-2-5        gagctcactttccaatgaggaa 22
KIV-2-4        gagctcactttccaatgaggaa 22
KIV-2-2        gagctcactttccaatgaggaa 22
KIV-2-1        gagctcactttccaatgaggaa 22
KIV-1          ---gacactttgcagtgagg-- 17
KIV-4          gagcacagtttccattgagaag 22
Protease       ---gacactttgcagtgagg-- 17
                    **  ***  **  ****
```

An additional hit (aagcagcacttgcatagaag--) is found also in KIV-5, but in wrong orientation and at a different position within the domain than what would be required for PCR amplification (i.e. about 120 bp downstream (in transcription direction) of a degenerated potential reverse primer binding site instead of upstream of it)

Alignment 2: CLUSTAL O(1.2.4) multiple sequence alignments for the reverse (rv) primer

Differences to the primer are shown in red

```
Primer_rv      AAACTCCAATCCCTCTCCTCTGC 23
KIV-3          aaactccaatccctctcctctgc 23
KIV-2B-3       aaactccaatccctctcctccat 23
KIV-1          aaactccaatgtccctcctctgt 23
KIV-2-1        aaactccaatccctctcctccat 23
KIV-2-2        aaactccaatccctctcctccat 23
KIV-2-4        aaactccaatccctctcctccat 23
KIV-2-5        aaactccaatccctctcctccat 23
KIV-2-6        aaactccaatccctctcctccat 23
KIV-4          aaattccaatctctctcctgc-- 21
KIV-5          aaagtccaatcactctcctctgt 23
KIV-6          aaactccagtctctctcctcagt 23
KIV-7          agactccagtctctctcctcagt 23
KIV-8          aaactccagtctctctccccagt 23
KIV-9          --aatccaatccctccccctagc- 20
KIV-10         aaactccagtctctttcctcagt 23
```

*   **** *   *   **

Alignment 3: Homologous region to the Sanger-sequenced KIV-3 region in KIV-2 and KIV-2B in hg38

Red: Differences between KIV-3 and KIV-2, but not KIV-2B

Yellow highlights: Differences between KIV-3 and any KIV-2, including KIV-2B. These positions can be inspected to evaluate whether the KIV-3 amplicons would coamplify KIV-2B.

```
CLUSTAL O(1.2.4) multiple sequence alignment


KIV-3_chr6:160611371-160611891       AGGAAGGTGAGGCAGCTTAACATTTCTCTTCTCTCAGACCCTTAGCTCCAAGGAAATCAT  60
KIV-2B_chr6:160633562-160634082      AGGAAGGAGAGGGAGCTTAACATTTCCCTTCTCTCAGACCCTTAGCTCCAAGGCAATCAT  60
KIV-2_chr6:160644656-160645175       AGGAAGGAGAGGGAGCTTAACATTTCCCTTCTCTCAGACCCTTAGCTCCAAGGCAATCAT  60
KIV-2_chr6:160639109-160639628       AGGAAGGAGAGGGAGCTTAACATTTCCCTTCTCTCAGACCCTTAGCTCCAAGGCAATCAT  60
KIV-2_chr6:160628016-160628535       AGGAAGGAGAGGGAGCTTAACATTTCCCTTCTCTCAGACCCTTAGCTCCAAGGCAATCAT  60
KIV-2_chr6:160622472-160622991       AGGAAGGAGAGGGAGCTTAACATTTCCCTTCTCTCAGACCCTTAGCTCCAAGGCAATCAT  60
KIV-2_chr6:160616926-160617445       AGGAAGGAGAGGGAGCTTAACATTTCCCTTCTCTCAGACCCTTAGCTCCAAGGCAATCAT  60
                                     ******* **** ************* ************************** ******

KIV-3_chr6:160611371-160611891       CCTGAGACATTTTGCTACACCATCTGAATCTGACACAAGTTGAGTTCGGAGAACTCAGCT  120
KIV-2B_chr6:160633562-160634082      CCTGAGACATTTTGCTACGCCATCTGCATCTGTCACAAGTTGAGTTCGGAGAACTCAGCT  120
KIV-2_chr6:160644656-160645175       CCTGAGACATTTTGCTACGCCATCTGCATCTGTCACAAGTTGAGTTCGGAGAACTCAGCT  120
KIV-2_chr6:160639109-160639628       CCTGAGACATTTTGCTACGCCATCTGCATCTGTCACAAGTTGAGTTCGGAGAACTCAGCT  120
KIV-2_chr6:160628016-160628535       CCTGAGACATTTTGCTACGCCATCTGCATCTGTCACAAGTTGAGTTCGGAGAACTCAGCT  120
KIV-2_chr6:160622472-160622991       CCTGAGACATTTTGCTACGCCATCTGCATCTGTCACAAGTTGAGTTCGGAGAACTCAGCT  120
KIV-2_chr6:160616926-160617445       CCTGAGACATTTTGCTACGCCATCTGCATCTGTCACAAGTTGAGTTCGGAGAACTCAGCT  120
                                     ****************** ******* ***** ***************************

KIV-3_chr6:160611371-160611891       TGAAGCATGTCTCTTGTCACAGAAACTTCAGTTGGCCCTTTATTCTCTTATGGTAAAGAA  180
KIV-2B_chr6:160633562-160634082      TGAGACACATCTCTTGTAACAGAAACTTCACTTGGCCCTTTCTTCTCTTATGGTAAAGAA  180
KIV-2_chr6:160644656-160645175       TGAGACACATCTCTTGTAACAGAAACTTCACTTGGCCCTTTCTTCTCTTATGGTAAAGAA  180
KIV-2_chr6:160639109-160639628       TGAGACACATCTCTTGTAACAGAAACTTCACTTGGCCCTTTCTTCTCTTATGGTAAAGAA  180
KIV-2_chr6:160628016-160628535       TGAGACACATCTCTTGTAACAGAAACTTCACTTGGCCCTTTCTTCTCTTATGGTAAAGAA  180
KIV-2_chr6:160622472-160622991       TGAGACACATCTCTTGTAACAGAAACTTCACTTGGCCCTTTCTTCTCTTATGGTAAAGAA  180
KIV-2_chr6:160616926-160617445       TGAGACACATCTCTTGTAACAGAAACTTCACTTGGCCCTTTCTTCTCTTATGGTAAAGAA  180
                                     ***  **  ********* ************ ********** *****************
```

```
KIV-3_chr6:160611371-160611891      CAAAGACATACGCATTTGGGTAGTATTCTGGGGTCCGACTATGCGAGTGTGGTGTCATAG 240
KIV-2B_chr6:160633562-160634082     CAAAGACATACGCATTTGGGTAGTATTCTGGGGTCCGACTATGCGAGTGTGGTGTCATAG 240
KIV-2_chr6:160644656-160645175      CAAAGACATACGCATTTGGGTAGTATTCTGGGGTCCGACTATGCGAGTGTGGTGTCATAG 240
KIV-2_chr6:160639109-160639628      CAAAGACATACGCATTTGGGTAGTATTCTGGGGTCCGACTATGCGAGTGTGGTGTCATAG 240
KIV-2_chr6:160628016-160628535      CAAAGACATACGCATTTGGGTAGTATTCTGGGGTCCGACTATGCGAGTGTGGTGTCATAG 240
KIV-2_chr6:160622472-160622991      CAAAGACATACGCATTTGGGTAGTATTCTGGGGTCCGACTATGCGAGTGTGGTGTCATAG 240
KIV-2_chr6:160616926-160617445      CAAAGACATACGCATTTGGGTAGTATTCTGGGGTCCGACTATGCGAGTGTGGTGTCATAG 240
                                    ************************************************************

KIV-3_chr6:160611371-160611891      ATGACCAAGCTTGGCAGGTTCTTCCAGTGACAGTGGTGGAGTATGTGCCTCGATAACTCT 300
KIV-2B_chr6:160633562-160634082     ATGACCAAGCTTGGCAGGTTCTTCCAGTGACAGTGGTGGAGTATGTGCCTCGATAACTCT 300
KIV-2_chr6:160644656-160645175      ATGACCAAGCTTGGCAGGTTCTTCCTGTGACAGTGGTGGAGTATGTGCCTCGATAACTCT 300
KIV-2_chr6:160639109-160639628      ATGACCAAGCTTGGCAGGTTCTTCCTGTGACAGTGGTGGAGTATGTGCCTCGATAACTCT 300
KIV-2_chr6:160628016-160628535      ATGACCAAGCTTGGCAGGTTCTTCCTGTGACAGTGGTGGAGTATGTGCCTCGATAACTCT 300
KIV-2_chr6:160622472-160622991      ATGACCAAGCTTGGCAGGTTCTTCCTGTGACAGTGGTGGAGTATGTGCCTCGATAACTCT 300
KIV-2_chr6:160616926-160617445      ATGACCAAGCTTGGCAGGTTCTTCCTGTGACAGTGGTGGAGTATGTGCCTCGATAACTCT 300
                                    ************************* **********************************

KIV-3_chr6:160611371-160611891      GTCCATTACCGTGGTAGCACTCCTGCACCCCAGGCCTCTGCTCAGTCGGTGCTGAAATGA 360
KIV-2B_chr6:160633562-160634082     GTCCATTACCGTGGTAGCACTCCTGCACCCCAGGCCTCTGCTCAGTCGGTGCTGAAATGA 360
KIV-2_chr6:160644656-160645175      GTCCATTACCATGGTAGCACTCCTGCACCCCAGGCCTTTGCTCAGTCGGTGCTGAAATGA 360
KIV-2_chr6:160639109-160639628      GTCCATTACCATGGTAGCACTCCTGCACCCCAGGCCTTTGCTCAGTCGGTGCTGAAATGA 360
KIV-2_chr6:160628016-160628535      GTCCATTACCATGGTAGCACTCCTGCACCCCAGGCCTTTGCTCAGTCGGTGCTGAAATGA 360
KIV-2_chr6:160622472-160622991      GTCCATTACCATGGTAGCACTCCTGCACCCCAGGCCTTTGCTCAGTCGGTGCTGAAATGA 360
KIV-2_chr6:160616926-160617445      GTCCATTACCATGGTAGCACTCCTGCACCCCAGGCCTTTGCTCAGTCGGTGCTGAAATGA 360
                                    ********** ************************** **********************

KIV-3_chr6:160611371-160611891      AAACACAAGAAAT-AAACTGAGTATCTCTGAGAATAACGAAATATGTGAAGCCATTTATG 419
KIV-2B_chr6:160633562-160634082     AAACACAAGAAAT-AAACTGAGTATCTCTGAGAATAACGAAATATGTGAAGCCATTTATG 419
KIV-2_chr6:160644656-160645175      AAACACGGGAAATCAAGCTGAGTATCTCTGAGCATAGAGAAACATGTGAAGCCATTTGTG 420
KIV-2_chr6:160639109-160639628      AAACACGGGAAATCAAGCTGAGTATCTCTGAGCATAGAGAAACATGTGAAGCCATTTGTG 420
KIV-2_chr6:160628016-160628535      AAACACGGGAAATCAAGCTGAGTATCTCTGAGCATAGAGAAACATGTGAAGCCATTTGTG 420
KIV-2_chr6:160622472-160622991      AAACACGGGAAATCAAGCTGAGTATCTCTGAGCATAGAGAAACATGTGAAGCCATTTGTG 420
KIV-2_chr6:160616926-160617445      AAACACGGGAAATCAAGCTGAGTATCTCTGAGCATAGAGAAACATGTGAAGCCATTTGTG 420
                                    ******  ***** ** *************** ***  **** ************** **
```

```
KIV-3_chr6:160611371-160611891       ACACAACCAGAAAGGAGTCTATGAGAATTATGAACGTTATCTTTCCCTTACCTGTAGGCA  479
KIV-2B_chr6:160633562-160634082      ACACAACCAGAAAGGAGTCTATGAGAATTACGAACGTTATCTTTCCCTTACCTGTAGGCA  479
KIV-2_chr6:160644656-160645175       ACACAACCAGAAAGGAGTCTATGAGAATTACGACCGTTCTCTTTTCCTTATCCATAGGCA  480
KIV-2_chr6:160639109-160639628       ACACAACCAGAAAGGAGTCTATGAGAATTACGACCGTTCTCTTTTCCTTATCCATAGGCA  480
KIV-2_chr6:160628016-160628535       ACACAACCAGAAAGGAGTCTATGAGAATTACGACCGTTCTCTTTTCCTTATCCATAGGCA  480
KIV-2_chr6:160622472-160622991       ACACAACCAGAAAGGAGTCTATGAGAATTACGACCGTTCTCTTTTCCTTATCCATAGGCA  480
KIV-2_chr6:160616926-160617445       ACACAACCAGAAAGGAGTCTATGAGAATTACGACCGTTCTCTTTTCCTTATCCATAGGCA  480
                                     ****************************** ** **** ***** ***** *  ******

KIV-3_chr6:160611371-160611891       GATGGATGGGAGAAAACCAACCAAAAAACATACAGCAAACCT  521
KIV-2B_chr6:160633562-160634082      GATGGATGGGAGAAAACCAACCAAAAAACATACAGCAAACCT  521
KIV-2_chr6:160644656-160645175       GATGGATGTGAGAAAACCGACCAACAAACAAACACCAAAG--  520
KIV-2_chr6:160639109-160639628       GATGGATGTGAGAAAACCGACCAACAAACAAACACCAAAG--  520
KIV-2_chr6:160628016-160628535       GATGGATGTGAGAAAACCGACCAACAAACAAACACCAAAG--  520
KIV-2_chr6:160622472-160622991       GATGGATGTGAGAAAACCGACCAACAAACAAACACCAAAG--  520
KIV-2_chr6:160616926-160617445       GATGGATGTGAGAAAACCGACCAACAAACAAACACCAAAG--  520
                                     ******** ********* ***** ***** *** ****
```

Alignment 4: Homologous region to the Sanger-sequenced KIV-3 region in KIV-2, KIV-2B and KIV-4 in hg38

```
CLUSTAL O(1.2.4) multiple sequence alignment


KIV-3_chr6:160611371-160611891       AGGAAGGTGAGGCAGCTTAACATTTCTCTTCTCTCAGACCCTTAGCTCCAAGGAAATCAT  60
KIV-2B_chr6:160633562-160634082      AGGAAGGAGAGGGAGCTTAACATTTCCCTTCTCTCAGACCCTTAGCTCCAAGGCAATCAT  60
KIV-2_chr6:160644656-160645175       AGGAAGGAGAGGGAGCTTAACATTTCCCTTCTCTCAGACCCTTAGCTCCAAGGCAATCAT  60
KIV-2_chr6:160639109-160639628       AGGAAGGAGAGGGAGCTTAACATTTCCCTTCTCTCAGACCCTTAGCTCCAAGGCAATCAT  60
KIV-2_chr6:160628016-160628535       AGGAAGGAGAGGGAGCTTAACATTTCCCTTCTCTCAGACCCTTAGCTCCAAGGCAATCAT  60
KIV-2_chr6:160622472-160622991       AGGAAGGAGAGGGAGCTTAACATTTCCCTTCTCTCAGACCCTTAGCTCCAAGGCAATCAT  60
KIV-2_chr6:160616926-160617445       AGGAAGGAGAGGGAGCTTAACATTTCCCTTCTCTCAGACCCTTAGCTCCAAGGCAATCAT  60
KIV-4_chr6:160604876-160605396       ---------------------ATTTGTCTTCTCTTAGACTCTTTGCTCAAAGACAATGTT  39
                                                          ****  ******* **** *** **** ***  ***  *

KIV-3_chr6:160611371-160611891       CCTGAGACATTTTGCTACACCATCTGAATCTGACACAAGTTGAGTTCGGAGAACTCAGCT  120
KIV-2B_chr6:160633562-160634082      CCTGAGACATTTTGCTACGCCATCTGCATCTGTCACAAGTTGAGTTCGGAGAACTCAGCT  120
KIV-2_chr6:160644656-160645175       CCTGAGACATTTTGCTACGCCATCTGCATCTGTCACAAGTTGAGTTCGGAGAACTCAGCT  120
KIV-2_chr6:160639109-160639628       CCTGAGACATTTTGCTACGCCATCTGCATCTGTCACAAGTTGAGTTCGGAGAACTCAGCT  120
KIV-2_chr6:160628016-160628535       CCTGAGACATTTTGCTACGCCATCTGCATCTGTCACAAGTTGAGTTCGGAGAACTCAGCT  120
KIV-2_chr6:160622472-160622991       CCTGAGACATTTTGCTACGCCATCTGCATCTGTCACAAGTTGAGTTCGGAGAACTCAGCT  120
KIV-2_chr6:160616926-160617445       CCTGAGACATTTTGCTACGCCATCTGCATCTGTCACAAGTTGAGTTCGGAGAACTCAGCT  120
KIV-4_chr6:160604876-160605396       CCTGAGACATTTTGCTATGCACTGTTCATCTGAGACAACTTGAGTCCTGAACACTCAGCT  99
                                     *****************  *  * *  *****  **** ****** * **  ********

KIV-3_chr6:160611371-160611891       TGAAGCATGTCTCTTGTCACAGAAACTTCAGTTGGCCCTTTATTCTCTTATGGTAAAGAA  180
KIV-2B_chr6:160633562-160634082      TGAGACACATCTCTTGTAACAGAAACTTCACTTGGCCCTTTCTTCTCTTATGGTAAAGAA  180
KIV-2_chr6:160644656-160645175       TGAGACACATCTCTTGTAACAGAAACTTCACTTGGCCCTTTCTTCTCTTATGGTAAAGAA  180
KIV-2_chr6:160639109-160639628       TGAGACACATCTCTTGTAACAGAAACTTCACTTGGCCCTTTCTTCTCTTATGGTAAAGAA  180
KIV-2_chr6:160628016-160628535       TGAGACACATCTCTTGTAACAGAAACTTCACTTGGCCCTTTCTTCTCTTATGGTAAAGAA  180
KIV-2_chr6:160622472-160622991       TGAGACACATCTCTTGTAACAGAAACTTCACTTGGCCCTTTCTTCTCTTATGGTAAAGAA  180
KIV-2_chr6:160616926-160617445       TGAGACACATCTCTTGTAACAGAAACTTCACTTGGCCCTTTCTTCTCTTATGGTAAAGAA  180
KIV-4_chr6:160604876-160605396       TGAAGCATGACTCTACTAACAGAAATTTCCACTGACCCTTCCTTCACTTATGGTAAAGAA  159
                                     ***  **   ****  * ******* ***   ** *****  *** **************

KIV-3_chr6:160611371-160611891       CAAAGACATACGCATTTGGGTAGTATTCTGGGGTCCGACTATGCGAGTGTGGTGTCATAG  240
KIV-2B_chr6:160633562-160634082      CAAAGACATACGCATTTGGGTAGTATTCTGGGGTCCGACTATGCGAGTGTGGTGTCATAG  240
KIV-2_chr6:160644656-160645175       CAAAGACATACGCATTTGGGTAGTATTCTGGGGTCCGACTATGCGAGTGTGGTGTCATAG  240
KIV-2_chr6:160639109-160639628       CAAAGACATACGCATTTGGGTAGTATTCTGGGGTCCGACTATGCGAGTGTGGTGTCATAG  240
KIV-2_chr6:160628016-160628535       CAAAGACATACGCATTTGGGTAGTATTCTGGGGTCCGACTATGCGAGTGTGGTGTCATAG  240
KIV-2_chr6:160622472-160622991       CAAAGACATACGCATTTGGGTAGTATTCTGGGGTCCGACTATGCGAGTGTGGTGTCATAG  240
```

```
KIV-2_chr6:160616926-160617445      CAAAGACATACGCATTTGGGTAGTATTCTGGGGTCCGACTATGCGAGTGTGGTGTCATAG  240
KIV-4_chr6:160604876-160605396      AATAGACATACGCATTTGGGTAGTATGCTGGGGTCCGACTATGCGAGTGTGGTGTCATAG  219
                                     * *********************** *********************************

KIV-3_chr6:160611371-160611891      ATGACCAAGCTTGGCAGGTTCTTCCAGTGACAGTGGTGGAGTATGTGCCTCGATAACTCT  300
KIV-2B_chr6:160633562-160634082     ATGACCAAGCTTGGCAGGTTCTTCCAGTGACAGTGGTGGAGTATGTGCCTCGATAACTCT  300
KIV-2_chr6:160644656-160645175      ATGACCAAGCTTGGCAGGTTCTTCCTGTGACAGTGGTGGAGTATGTGCCTCGATAACTCT  300
KIV-2_chr6:160639109-160639628      ATGACCAAGCTTGGCAGGTTCTTCCTGTGACAGTGGTGGAGTATGTGCCTCGATAACTCT  300
KIV-2_chr6:160628016-160628535      ATGACCAAGCTTGGCAGGTTCTTCCTGTGACAGTGGTGGAGTATGTGCCTCGATAACTCT  300
KIV-2_chr6:160622472-160622991      ATGACCAAGCTTGGCAGGTTCTTCCTGTGACAGTGGTGGAGTATGTGCCTCGATAACTCT  300
KIV-2_chr6:160616926-160617445      ATGACCAAGCTTGGCAGGTTCTTCCTGTGACAGTGGTGGAGTATGTGCCTCGATAACTCT  300
KIV-4_chr6:160604876-160605396      ATGACCAAGCTTGGCAGGTTCTTCCTGTGACAGTAATGAAGTATGTGCCTTGATAACTCT  279
                                    ************************* ********  ** *********** *********

KIV-3_chr6:160611371-160611891      GTCCATTACCGTGGTAGCACTCCTGCACCCCAGGCCTCTGCTCAGTCGGTGCTGAAATGA  360
KIV-2B_chr6:160633562-160634082     GTCCATTACCGTGGTAGCACTCCTGCACCCCAGGCCTCTGCTCAGTCGGTGCTGAAATGA  360
KIV-2_chr6:160644656-160645175      GTCCATTACCATGGTAGCACTCCTGCACCCCAGGCCTTTGCTCAGTCGGTGCTGAAATGA  360
KIV-2_chr6:160639109-160639628      GTCCATTACCATGGTAGCACTCCTGCACCCCAGGCCTTTGCTCAGTCGGTGCTGAAATGA  360
KIV-2_chr6:160628016-160628535      GTCCATTACCATGGTAGCACTCCTGCACCCCAGGCCTTTGCTCAGTCGGTGCTGAAATGA  360
KIV-2_chr6:160622472-160622991      GTCCATTACCATGGTAGCACTCCTGCACCCCAGGCCTTTGCTCAGTCGGTGCTGAAATGA  360
KIV-2_chr6:160616926-160617445      GTCCATTACCATGGTAGCACTCCTGCACCCCAGGCCTTTGCTCAGTCGGTGCTGAAATGA  360
KIV-4_chr6:160604876-160605396      GTCCATTTCCGTGGTAGCACTCCTGCACCCCAGGCCTTTGCTCAGTTGGTGCTGAAATGA  339
                                    ******* ** ************************** ******** *************

KIV-3_chr6:160611371-160611891      AAACACAAGAAAT-AAACTGAGTATCTCTGAGAATAACGAAATATGTGAAGCCATTTATG  419
KIV-2B_chr6:160633562-160634082     AAACACAAGAAAT-AAACTGAGTATCTCTGAGAATAACGAAATATGTGAAGCCATTTATG  419
KIV-2_chr6:160644656-160645175      AAACACGGGAAATCAAGCTGAGTATCTCTGAGCATAGAGAAACATGTGAAGCCATTTGTG  420
KIV-2_chr6:160639109-160639628      AAACACGGGAAATCAAGCTGAGTATCTCTGAGCATAGAGAAACATGTGAAGCCATTTGTG  420
KIV-2_chr6:160628016-160628535      AAACACGGGAAATCAAGCTGAGTATCTCTGAGCATAGAGAAACATGTGAAGCCATTTGTG  420
KIV-2_chr6:160622472-160622991      AAACACGGGAAATCAAGCTGAGTATCTCTGAGCATAGAGAAACATGTGAAGCCATTTGTG  420
KIV-2_chr6:160616926-160617445      AAACACGGGAAATCAAGCTGAGTATCTCTGAGCATAGAGAAACATGTGAAGCCATTTGTG  420
KIV-4_chr6:160604876-160605396      AAAGAAAAGAAATCAAACTGAGTGTTTCCAAGAAGAGACAAACATGTGAAGCCACTTATG  399
                                    *** *   ***** ** ****** * **  ** * *   *** *********** ** **
```

```
KIV-3_chr6:160611371-160611891      ACACAACCAGAAAG-GAGTCTATGAGAATTATGAA----------------------CGT  456
KIV-2B_chr6:160633562-160634082     ACACAACCAGAAAG-GAGTCTATGAGAATTACGAA----------------------CGT  456
KIV-2_chr6:160644656-160645175      ACACAACCAGAAAG-GAGTCTATGAGAATTACGAC----------------------CGT  457
KIV-2_chr6:160639109-160639628      ACACAACCAGAAAG-GAGTCTATGAGAATTACGAC----------------------CGT  457
KIV-2_chr6:160628016-160628535      ACACAACCAGAAAG-GAGTCTATGAGAATTACGAC----------------------CGT  457
KIV-2_chr6:160622472-160622991      ACACAACCAGAAAG-GAGTCTATGAGAATTACGAC----------------------CGT  457
KIV-2_chr6:160616926-160617445      ACACAACCAGAAAG-GAGTCTATGAGAATTACGAC----------------------CGT  457
KIV-4_chr6:160604876-160605396      GCACAAACCAGAAAAAAGTCTCTGAGAATTATGACCTCAGGAGAATATGACAAGTAACAT  459
                                     ***** *   **   ***** ********* **                       * *

KIV-3_chr6:160611371-160611891      TATCTTTCCCTTACCTGTAGGCAGATGGATGGGAGAAAACCAACCAAAAAACATACAGCA  516
KIV-2B_chr6:160633562-160634082     TATCTTTCCCTTACCTGTAGGCAGATGGATGGGAGAAAACCAACCAAAAAACATACAGCA  516
KIV-2_chr6:160644656-160645175      TCTCTTTTCCTTATCCATAGGCAGATGGATGTGAGAAAACCGACCAACAAACAAACACCA  517
KIV-2_chr6:160639109-160639628      TCTCTTTTCCTTATCCATAGGCAGATGGATGTGAGAAAACCGACCAACAAACAAACACCA  517
KIV-2_chr6:160628016-160628535      TCTCTTTTCCTTATCCATAGGCAGATGGATGTGAGAAAACCGACCAACAAACAAACACCA  517
KIV-2_chr6:160622472-160622991      TCTCTTTTCCTTATCCATAGGCAGATGGATGTGAGAAAACCGACCAACAAACAAACACCA  517
KIV-2_chr6:160616926-160617445      TCTCTTTTCCTTATCCATAGGCAGATGGATGTGAGAAAACCGACCAACAAACAAACACCA  517
KIV-4_chr6:160604876-160605396      TCTTGTTTCTTTATTTGTAGGCAGATGGACTTGAGAAAAGCAACAACCAACCAACAAACA  519
                                    * *  ** * ***    ************   ******* * ** *  ** **   * **

KIV-3_chr6:160611371-160611891      AACCT   521
KIV-2B_chr6:160633562-160634082     AACCT   521
KIV-2_chr6:160644656-160645175      AAG--   520
KIV-2_chr6:160639109-160639628      AAG--   520
KIV-2_chr6:160628016-160628535      AAG--   520
KIV-2_chr6:160622472-160622991      AAG--   520
KIV-2_chr6:160616926-160617445      AAG--   520
KIV-4_chr6:160604876-160605396      AA---   521
                                    **
```
