# Supplementary material for: Resolving intra-repeat variation in medically relevant VNTRs from short-read sequencing data using the cardiovascular risk gene LPA as a model
Source: Genome Biol. 2024 Jun 26;25:167. doi: 10.1186/s13059-024-03316-5 (PMC11201333; doi:10.1186/s13059-024-03316-5)
Supplement: Supplementary file 4 — Additional file 4. Supplementary note for LD with KIV-2 SNPs. [file 13059_2024_3316_MOESM4_ESM.pdf]

Additional File 4

Supplementary note for calculation of linkage disequilibrium for KIV-2 SNPs

While the T/C allele in KIV-3 (exon 1 position 86) is available as genotype obtained with Sanger-sequencing, the KIV-2B status can be reported only as carrier status, as no phasing is possible when a mutant allele occurs more than one time. Deep sequencing provides “only” the carrier status, not a true genotype. Even more, the KIV-2B carrier status is defined based on the carrier status of the three canonical KIV-2B variants in KIV-2B exon 1, position 14, 41 and 86 (positions 594, 621 and 666 in our KIV-2 reference sequence). This complicates the calculation of LD values, which are usually based on genotypes.

However, to further support the signature-sequence, we have approximated the LD values in the 66 samples with available KIV-2B carrier status (from our previous deep-sequencing approach from Coassin & Schönherr, J Lipid Res 57, 2019). We have encoded the KIV-2 position 594 (KIV-2 exon 1 position 14; as proxy for the other two canonical KIV-2B variants) as either wild-type or heterozygous (A/A=16 and A/G=50). Since the signature sequence is based on the presence/absence of the T allele at position 86 in exon 1 of KIV-3, we had two options to encode the genotype of this variant:

1. Option 1: C/C=11, T/C=31 and T/T=24. In this case, all heterozygous individuals (29 T/C and 2 T/A) were encoded as T/C.
2. Option 2: C/C=11, T/C=55. In this case, all heterozygous and homozygous for the mutations were encoded as T/C. This reflects the situation of KIV-2 variants, as explained above.

For KIV-2 variants, all carriers are encoded as heterozygous and there are no individuals encoded as homozygous.

We used of the R package *genetics* to calculate the LD between KIV-2 exon 1 position 14 and KIV-3 exon 1 position 86 and obtained:

Option 1:

- D´=0.9995701
- R-squared=0.41

Option 2:

- D´=0.9997041
- R-squared=0.85.

Interpretation:

In option 1, the LD is calculated with the genotype of the signature position (KIV-3, exon 1 position 86). This situation resembles a LD between two SNPs with highly different MAF and therefore D´ does better reflect the LD between these two variants compared to r-squared (~0.41).

In option 2, also the KIV-3 variant is encoded either as wild-type or heterozygous, which resembles a carrier status (respectively presence/absence). Thus, the unbalanced frequency of option 1 is partially resolved and r-squared is ~0.85.

In both cases, the D´ is very high (~0.99), indicating the absence of recombination between the T allele in the signature position (KIV-3, exon 1 position 86) and the presence of KIV-2B repeats.
