# Supplementary material for: Resolving intra-repeat variation in medically relevant VNTRs from short-read sequencing data using the cardiovascular risk gene LPA as a model
Source: Genome Biol. 2024 Jun 26;25:167. doi: 10.1186/s13059-024-03316-5 (PMC11201333; doi:10.1186/s13059-024-03316-5)
Supplement: Supplementary file 5 — Additional file 5. Sequences alignments. [file 13059_2024_3316_MOESM5_ESM.pdf]

Sequence Alignments

Multiple sequence alignment of the first exon (±100 bp into the introns) of each KIV (KIV-1 to KIV-3) domain of the *LPA* gene in the human reference genome hg19, which includes 6 KIV-2 repeats. Capital letters are used for the bases within the exons.

```
KIV-1_Exon1         ----------tgaacattaagaagagatttctaaaaaagagcttgcacatgagcatagtt    50
KIV-2.1_Exon1       ------------------------agtttcctagaaaggtgctacctcgtgagctcactt    36
KIV-2.2_Exon1       ------------------------agtttcctagaaaggtgctacctcgtgagctcactt    36
KIV-2.3B_Exon1      ---------------------------ttcatagaaaggtgctacttcgtgagcgcactt    33
KIV-2.4_Exon1       ------------------------agtttcctagaaaggtgctacctcgtgagctcactt    36
KIV-2.5_Exon1       ------------------------agtttcctagaaaggtgctacctcgtgagctcactt    36
KIV-2.6_Exon1       ------------------------agtttcctagaaaggtgctacctcgtgagctcactt    36
KIV-3_Exon1         ---------------------------ttcatagaaaggtgctacttcgtgagcgcactt    33
KIV-4_Exon1         -----------------------------------------------------------t    1
KIV-5_Exon1         ----------------------agagattcctagaaatgagcttccatgttagtggagtt    38
KIV-6_Exon1         -----------gaacacccagaagatgtctatattaaagagcatgcacatgagtgcaatt    49
KIV-7_Exon1         ------------aacacccaaaagagatctttgccaaagaacaggcacatgagtgcaatt    48
KIV-8_Exon1         --------------------------gtgtaagcaaaagaccattcacatgagtgtaatt    34
KIV-9_Exon1         tgtggttctcatgaaaaccagcagagattcatatgatggag-ctgcacatgaatgtaatt    59
KIV-10_Exon1        ---gttttcatgaacaaccagtagagagccatacgaaagagcttgcacatgagtctttgt    57
                                                                               *

KIV-1_Exon1         tctggtgagaagaattctgatatgt--------------------taacttcctctaaac    90
KIV-2.1_Exon1       tccaatgaggaatc---tgatc-------------------tgttgtgttt-ctctaagg    73
KIV-2.2_Exon1       tccaatgaggaatc---tgatc-------------------tgttgtgttt-ctctaagg    73
KIV-2.3B_Exon1      tgcagtgagaaggagtctgttc-------------------tgttctgtttttctaagga    74
KIV-2.4_Exon1       tccaatgaggaatc---tgatc-------------------tgttgtgttt-ctctaagg    73
KIV-2.5_Exon1       tccaatgaggaatc---tgatc-------------------tgttgtgttt-ctctaagg    73
KIV-2.6_Exon1       tccaatgaggaatc---tgatc-------------------tgttgtgttt-ctctaagg    73
KIV-3_Exon1         tgcagtgagaaggagtctgttc-------------------tgttctgtttttctaagga    74
KIV-4_Exon1         tcca-ttgagaagccctctcat-------------------ttgtcctttttttctaagc    41
KIV-5_Exon1         tccgatgagaagca---atctg-------------------atatttcttttccactaag    76
KIV-6_Exon1         ttgactgataggcactctgatcttt--------------------cctttggtgcctgtg    89
KIV-7_Exon1         ttgactgataggcactctgatctgt--------------------cctttggtgcccagg    88
KIV-8_Exon1         ttgaagtataggcactctgatctgttttttgtttgtttctttgtttgtttgttttccagg    94
KIV-9_Exon1         tccaatgtccagcattctcctctg----------------------ttctttatctttag    97
KIV-10_Exon1        tccaattgtaagagcactgataggt--------------------ccttttcccaccagg    97
                    *                                                *

KIV-1_Exon1         ttttaaataaaatatttctaagaattaaataaagttctagaatgatatgaatctattcct    150
KIV-2.1_Exon1       tgtcaggtgaaatatttccaagaacttactacagttctagaatgggaggaatctgttgct    133
KIV-2.2_Exon1       tgtcaggtgaaatatttccaagaacttactacagttctagaatgggaggaatctgttgct    133
KIV-2.3B_Exon1      tttcaggtgaaatatttcctagaacttactacagttctagattggtaggaatctgtaggt    134
KIV-2.4_Exon1       tgtcaggtgaaatatttccaagaacttactacagttctagaatgggaggaatctgttgct    133
KIV-2.5_Exon1       tgtcaggtgaaatatttccaagaacttactacagttctagaatgggaggaatctgttgct    133
KIV-2.6_Exon1       tgtcaggtgaaatatttccaagaacttactacagttctagaatgggaggaatctgttgct    133
KIV-3_Exon1         tttcaggtgaaatatttcctagaacttactacagttctagattggtaggaatctgtaggt    134
KIV-4_Exon1         ttttatgtgaaatatttctaagaacttactacagttctaaagtgttaggaatttgtttct    101
KIV-5_Exon1         ttttacatgaaatatttctaagaacttactacagttctagaatggtaggcatctcttact    136
KIV-6_Exon1         ttttaaaggaaatctttctaagaactcgttaaagttctagaatgctatgaatctttgggt    149
KIV-7_Exon1         ttttaaagaaaatctttctaaaaactcattgaagttccagaatgctatgaatctttgagc    148
KIV-8_Exon1         gttgaattaaaatatttatgactacttattaaatttctagaatcctataagtctatttgt    154
KIV-9_Exon1         atttaaaaataatgtttctatgaacttattaaaattctagaatactatgaatctactggg    157
KIV-10_Exon1        ttttgaatataaaatttctaagaacttattaaaatattagaatgttattaatctattgtt    157
                      *       **  ***      * *   *  * *   * * *   *    * *

KIV-1_Exon1         ttggtttt-------------------------tt-gcacgtctgtctgcctgctaatca    184
KIV-2.1_Exon1       ttggtgtttgttt---------gttggtcggttttctcacatccatctgcctatggataa    184
KIV-2.2_Exon1       ttggtgtttgttt---------gttggtcggttttctcacatccatctgcctatggataa    184
KIV-2.3B_Exon1      ttgctgtatgttt---------tttggttggttttctcccatccatctgcctacaggtaa    185
KIV-2.4_Exon1       ttggtgtttgttt---------gttggtcggttttctcacatccatctgcctatggataa    184
KIV-2.5_Exon1       ttggtgtttgttt---------gttggtcggttttctcacatccatctgcctatggataa    184
KIV-2.6_Exon1       ttggtgtttgttt---------gttggtcggttttctcacatccatctgcctatggataa    184
KIV-3_Exon1         ttgctgtatgttt---------tttggttggttttctcccatccatctgcctacaggtaa    185
KIV-4_Exon1         ttggtgtttttgtttgttggttggttgttgcttttctcaagtccatctgcctacaaataa    161
KIV-5_Exon1         ttcgtgtttgtttgt-------------gtgttttctcatgtccatttgcctattaataa    183
KIV-6_Exon1         tttattat-------------------------tg-gtatgtccatctgcctgctagtac    183
KIV-7_Exon1         tttgttat-------------------------tg-gcatgtccatctgcctactaatgt    182
KIV-8_Exon1         atttttat-------------------------tctacatttcaatttgcatgctaatat    189
KIV-9_Exon1         tcttttca-------------------------c------atccttttgc-tactagtag    185
KIV-10_Exon1        tttgcttc-------------------------a--gcatgtccttctgcttgtgagtat    190
                                                             **  * *   *     *

KIV-1_Exon1         agagaagagaatggt----------------------cgtaattctcagagactttttcc    222
KIV-2.1_Exon1       ggaaaagagaacggt----------------------cgtaattctcata-gactc-ctt    220
KIV-2.2_Exon1       ggaaaagagaacggt----------------------cgtaattctcata-gactc-ctt    220
```

```
KIV-2.3B_Exon1    gggaaagataacgtt----------------------cgtaattctcata-gactc-ctt    221
KIV-2.4_Exon1     ggaaaagagaacggt----------------------cgtaattctcata-gactc-ctt    220
KIV-2.5_Exon1     ggaaaagagaacggt----------------------cgtaattctcata-gactc-ctt    220
KIV-2.6_Exon1     ggaaaagagaacggt----------------------cgtaattctcata-gactc-ctt    220
KIV-3_Exon1       gggaaagataacgtt----------------------cataattctcata-gactc-ctt    221
KIV-4_Exon1       agaaacaagaatgttacttgtcatattctcctgaggtcataattctcaga-gactttttt    220
KIV-5_Exon1       agaatagagaatggt----------------------tgtaaatctcagt-gactctttt    220
KIV-6_Exon1       agaacagagcatggt----------------------agtctttctcagagacaatgatc    221
KIV-7_Exon1       agaacagagcatggt----------------------cgtcattttcagagatgatgtcc    220
KIV-8_Exon1       agaagagtgtaaatt----------------------gttaatcctca---gattattcc    224
KIV-9_Exon1       aaaaaagaatagtaa----------------------taattttcaga---ggctactgt    220
KIV-10_Exon1      actaaagagaacagt----------------------cataattctga---aactactgt    225
                            *                                    *

KIV-1_Exon1       tgtttgtgt--cataaatgacttcacatttttttctgttctaagaactattcagcttgat    280
KIV-2.1_Exon1     tctggttgtgtcacaaatggcttcacatgtttctctatgctcagagatactcagcttgat    280
KIV-2.2_Exon1     tctggttgtgtcacaaatggcttcacatgtttctctatgctcagagatactcagcttgat    280
KIV-2.3B_Exon1    tctggttgtgtcataaatggcttcacatatttcgttattctcagagatactcagttt-at    280
KIV-2.4_Exon1     tctggttgtgtcacaaatggcttcacatgtttctctatgctcagagatactcagcttgat    280
KIV-2.5_Exon1     tctggttgtgtcacaaatggcttcacatgtttctctatgctcagagatactcagcttgat    280
KIV-2.6_Exon1     tctggttgtgtcacaaatggcttcacatgtttctctatgctcagagatactcagcttgat    280
KIV-3_Exon1       tctggttgtgtcataaatggcttcacatatttcgttattctcagagatactcagttt-at    280
KIV-4_Exon1       ctggtttgtgccataagtggcttcacatgtttgtctcttcttggaaacactcagtttgat    280
KIV-5_Exon1       ttggtttatgtcataaatggcttcctgtatttttctgttctaggaaataataagcttgat    280
KIV-6_Exon1       ctgtttc-agtcacagatttcttctgatgcttctgtgttctagaaattactcagcttgat    280
KIV-7_Exon1       tgtttctatcatggattttttttctcatgcttctgtgttctggaaattactcagtttgtt    280
KIV-8_Exon1       actttgtgtgtcataatttttttcac--atttccctttttctaggcaatactgagcttg--    280
KIV-9_Exon1       ccagtatgtgacataaattgtctcccatgtttctctgctcatgcaattactgagtatgat    280
KIV-10_Exon1      cctgtttgtgtcataaattgcttcacatgtttctgcatactagtagttactcagcttgat    285
                                *  *    **      **       *        * * **  *

KIV-1_Exon1       ttcttctgttttaattttagCAGCACCTGAGCAAAGCCATGTGGTCCAGGATTGCTACCA    340
KIV-2.1_Exon1     ttcccgtgttttcatttcagCACCGACTGAGCAAAGGCCTGGGGTGCAGGAGTGCTACCA    340
KIV-2.2_Exon1     ttcccgtgttttcatttcagCACCGACTGAGCAAAGGCCTGGGGTGCAGGAGTGCTACCA    340
KIV-2.3B_Exon1    ttcttgtgttttcatttcagCACCGACTGAGCAGAGGCCTGGGGTGCAGGAGTGCTACCA    340
KIV-2.4_Exon1     ttcccgtgttttcatttcagCACCGACTGAGCAAAGGCCTGGGGTGCAGGAGTGCTACCA    340
KIV-2.5_Exon1     ttcccgtgttttcatttcagCACCGACTGAGCAAAGGCCTGGGGTGCAGGAGTGCTACCA    340
KIV-2.6_Exon1     ttcccgtgttttcatttcagCACCGACTGAGCAAAGGCCTGGGGTGCAGGAGTGCTACCA    340
KIV-3_Exon1       ttcttgtgttttcatttcagCACCGACTGAGCAGAGGCCTGGGGTGCAGGAGTGCTACCA    340
KIV-4_Exon1       ttcttttcttttcatttcagCACCAACTGAGCAAAGGCCTGGGGTGCAGGAGTGCTACCA    340
KIV-5_Exon1       gtcttctgttttaatttcagCACTGACTGAGGAAACCCCCGGGGTACAGGACTGCTACTA    340
KIV-6_Exon1       ttctcctctttgaatttcagCACCAACGGAGCAAAGCCCCGGGGTCCAGGATTGCTACCA    340
KIV-7_Exon1       ttctcctctttgaatttcagCACCAACGGAGCAAAGCCCCACAGTCCAGGACTGCTACCA    340
KIV-8_Exon1       attttctcttttaatttcagCACCAACTGAAAACAGCACTGGGGTCCAGGACTGCTACCG    340
KIV-9_Exon1       ttattttattttaatttcagCACCACCTGAGAAAAGCCCTGTGGTCCAGGATTGCTACCA    340
KIV-10_Exon1      -----tttgtctattttcagCACCAACTGAGCAAACCCCTGTGGTCCGGCAGTGCTACCA    340
                        *  *    *** ****    * **  * *        ** * * * ******

KIV-1_Exon1       TGGTGATGGACAGAGTTATCGAGGCACGTACTCCACCACTGTCACAGGAAGGACCTGCCA    400
KIV-2.1_Exon1     TGGTAATGGACAGAGTTATCGAGGCACATACTCCACCACTGTCACAGGAAGAACCTGCCA    400
KIV-2.2_Exon1     TGGTAATGGACAGAGTTATCGAGGCACATACTCCACCACTGTCACAGGAAGAACCTGCCA    400
KIV-2.3B_Exon1    CGGTAATGGACAGAGTTATCGAGGCACATACTCCACCACTGTCACTGGAAGAACCTGCCA    400
KIV-2.4_Exon1     TGGTAATGGACAGAGTTATCGAGGCACATACTCCACCACTGTCACAGGAAGAACCTGCCA    400
KIV-2.5_Exon1     TGGTAATGGACAGAGTTATCGAGGCACATACTCCACCACTGTCACAGGAAGAACCTGCCA    400
KIV-2.6_Exon1     TGGTAATGGACAGAGTTATCGAGGCACATACTCCACCACTGTCACAGGAAGAACCTGCCA    400
KIV-3_Exon1       CGGTAATGGACAGAGTTATCGAGGCACATACTCCACCACTGTCACTGGAAGAACCTGCCA    400
KIV-4_Exon1       CGGAAATGGACAGAGTTATCAAGGCACATACTTCATTACTGTCACAGGAAGAACCTGCCA    400
KIV-5_Exon1       CCATTATGGACAGAGTTACCGAGGCACATACTCCACCACTGTCACAGGAAGAACTTGCCA    400
KIV-6_Exon1       TGGTGATGGACAGAGTTATCGAGGCTCATTCTCTACCACTGTCACAGGAAGGACATGTCA    400
KIV-7_Exon1       TGGTGATGGACAGAGTTATCGAGGCTCATTCTCCACCACTGTTACAGGAAGGACATGTCA    400
KIV-8_Exon1       AGGTGATGGACAGAGTTATCGAGGCACACTCTCCACCACTATCACAGGAAGAACATGTCA    400
KIV-9_Exon1       TGGTGATGGACGGAGTTATCGAGGCATATCCTCCACCACTGTCACAGGAAGGACCTGTCA    400
KIV-10_Exon1      TGGTAATGGCCAGAGTTATCGAGGCACATTCTCCACCACTGTCACAGGAAGGACATGTCA    400
                       **** * ****** * ****     **  *  *** * ** ***** ** ** **

KIV-1_Exon1       AGCTTGGTCATCTATGACACCACATCAACATAATAGGACCACAGAAAACTACCCAAATGC    460
KIV-2.1_Exon1     AGCTTGGTCATCTATGACACCACACTCGCATAGTCGGACCCCAGAATACTACCCAAATGC    460
KIV-2.2_Exon1     AGCTTGGTCATCTATGACACCACACTCGCATAGTCGGACCCCAGAATACTACCCAAATGC    460
KIV-2.3B_Exon1    AGCTTGGTCATCTATGACACCACACTCGCATAGTCGGACCCCAGAATACTACCCAAATGC    460
KIV-2.4_Exon1     AGCTTGGTCATCTATGACACCACACTCGCATAGTCGGACCCCAGAATACTACCCAAATGC    460
KIV-2.5_Exon1     AGCTTGGTCATCTATGACACCACACTCGCATAGTCGGACCCCAGAATACTACCCAAATGC    460
KIV-2.6_Exon1     AGCTTGGTCATCTATGACACCACACTCGCATAGTCGGACCCCAGAATACTACCCAAATGC    460
KIV-3_Exon1       AGCTTGGTCATCTATGACACCACACTCGCATAGTCGGACCCCAGAATACTACCCAAATGC    460
KIV-4_Exon1       AGCTTGGTCATCTATGACACCACACTCGCATAGTCGGACCCCAGCATACTACCCAAATGC    460
KIV-5_Exon1       AGCTTGGTCATCTATGACACCACACCAGCATAGTCGGACCCCAGAAAACTACCCAAATGC    460
KIV-6_Exon1       GTCTTGGTCCTCTATGACACCACACTGGCATCAGAGGACAACAGAATATTATCCAAATGG    460
KIV-7_Exon1       GTCTTGGTCCTCTATGACACCACACTGGCATCAGAGAACCACAGAATACTACCCAAATGG    460
KIV-8_Exon1       GTCTTGGTCGTCTATGACACCACATTGGCATCGGAGGATCCCATTATACTATCCAAATGC    460
```

```
KIV-9_Exon1         ATCTTGGTCATCTATGATACCACACTGGCATCAGAGGACCCCAGAAAACTACCCAAATGC   460
KIV-10_Exon1        ATCTTGGTCATCCATGACACCACACCGGCATCAGAGGACCCCAGAAAACTACCCAAATGA   460
                      ******* ** **** ******    ***    * *   **  * * ** *******

KIV-1_Exon1         gtatgtcattaatcttacagta-agcaaaacaaggtccaagtaaaatttgtcttagaaaa   519
KIV-2.1_Exon1       gtatgtctttgttctttaccataagagaagaaagggccaagtgaagtttctgttacaaga   520
KIV-2.2_Exon1       gtatgtctttgttctttaccataagagaagaaagggccaagtgaagtttctgttacaaga   520
KIV-2.3B_Exon1      gtatgtctttgttctttaccataagagaagaaagggccaagtgaagtttctgttacaaga   520
KIV-2.4_Exon1       gtatgtctttgttctttaccataagagaagaaagggccaagtgaagtttctgttacaaga   520
KIV-2.5_Exon1       gtatgtctttgttctttaccataagagaagaaagggccaagtgaagtttctgttacaaga   520
KIV-2.6_Exon1       gtatgtctttgttctttaccataagagaagaaagggccaagtgaagtttctgttacaaga   520
KIV-3_Exon1         gtatgtctttgttctttaccataagagaataaagggccaactgaagtttctgtgacaaga   520
KIV-4_Exon1         gtatgtctattttctttaccataagtgaaggaagggtcagtggaaatttctgttagtaga   520
KIV-5_Exon1         gtacgtctttgttctttaccataagcgaaggaagggccaatggaagtttctgttagaaga   520
KIV-6_Exon1         gtacaaccttgagttttcttcaaagacagacagcagccccccttacatttctcttggaagg   520
KIV-7_Exon1         gtatgtctttgagttttctcccaagagaaacagccacccacttaaatttctcctggaaga   520
KIV-8_Exon1         gtatgtctatcatgttagccataaaaggaacaatagtcaactaaaatttctcttagctgg   520
KIV-9_Exon1         gtatgtatttgattaaaaccataagaggagcaacagccaactcaaatattggttagaaga   520
KIV-10_Exon1        gtatgtctttgatgttacttgtaagaggagcaacagccaacttaagttcctcctagaaga   520
                    ***      *             *       *     *     *  *

KIV-1_Exon1         ggtgtgcgtcaagctaacttcttatgattaaatttttctcacacatagaatgcatggcaa   579
KIV-2.1_Exon1       gatgtgtctcaagctgagttctccgaactcaacttgtgacagatgcagatggcgtagcaa   580
KIV-2.2_Exon1       gatgtgtctcaagctgagttctccgaactcaacttgtgacagatgcagatggcgtagcaa   580
KIV-2.3B_Exon1      gatgtgtctcaagctgagttctccgaactcaacttgtgacagatgcagatggcgtagcaa   580
KIV-2.4_Exon1       gatgtgtctcaagctgagttctccgaactcaacttgtgacagatgcagatggcgtagcaa   580
KIV-2.5_Exon1       gatgtgtctcaagctgagttctccgaactcaacttgtgacagatgcagatggcgtagcaa   580
KIV-2.6_Exon1       gatgtgtctcaagctgagttctccgaactcaacttgtgacagatgcagatggcgtagcaa   580
KIV-3_Exon1         gacatgcttcaagctgagttctccgaactcaacttgtgtcagattcagatggtgtagcaa   580
KIV-4_Exon1         gtcatgcttcaagctgagtgttcaggactcaagttgtctcagatgaacagtgcatagcaa   580
KIV-5_Exon1         gtcatgcttcaaggtgactgctcaggactcaacttggctcagatgcagaggaacatttcc   580
KIV-6_Exon1         gccatgcttccaactaacttcttatgacaa-atttatctcagatctggaatgttgggtag   579
KIV-7_Exon1         gccatgcttccagctaacttcttatgacccaatttctctcagacccagaatgttggacag   580
KIV-8_Exon1         cccatgctacaagctcacttcctaggtccaaatttctcatagactcagagtttgtagcaa   580
KIV-9_Exon1         cccatgctttaagctcacttcctagggacaaatttctc-tt-agactcacattttggcaa   578
KIV-10_Exon1        gccttgcttcaagctaacttgttaggacaaatttccct-tagacccagaaggtgtgtcaa   579
                        **     *  * * *              *        *     *

KIV-1_Exon1         aatgtctgagaaaca-ttactttgagcaaagagtatgatagaagagaaatgttaagctgg   638
KIV-2.1_Exon1       aatgtctcaggatgattgccttggagctaagggtctgagagaagggaaatgttaagctcc   640
KIV-2.2_Exon1       aatgtctcaggatgattgccttggagctaagggtctgagagaagggaaatgttaagctcc   640
KIV-2.3B_Exon1      aatgtctcaggatgattgccttggagctaagggtctgagagaagggaaatgttaagctcc   640
KIV-2.4_Exon1       aatgtctcaggatgattgccttggagctaagggtctgagagaagggaaatgttaagctcc   640
KIV-2.5_Exon1       aatgtctcaggatgattgccttggagctaagggtctgagagaagggaaatgttaagctcc   640
KIV-2.6_Exon1       aatgtctcaggatgattgccttggagctaagggtctgagagaagggaaatgttaagctcc   640
KIV-3_Exon1         aatgtctcaggatgatttccttggagctaagggtctgagagaagagaaatgttaagctgc   640
KIV-4_Exon1         aatgtctcaggaacattgtctttgagcaaagagtctaagagaagacaaatgttaatctgg   640
KIV-5_Exon1         tgtgagcaaaagttcttagagaagactttgtttttttgagacagagtcttgctttgttgc   640
KIV-6_Exon1         aatgtctcaggcttc-tttcttcaggcacagtgtctgaaaggagagaaatgtcaggccag   638
KIV-7_Exon1         aatgtctcaggcttc-ttgctttgggcacagggtctgagaggagagaaatgtcaggccag   639
KIV-8_Exon1         aatgtctcaggaaacttacttttgagcaaaaggtctgaatgaagagaagttttaggattg   640
KIV-9_Exon1         aatgtctcaggacctttgcttttgagcaaagagtctaagagaagagaaattttaggcctg   638
KIV-10_Exon1        aatgtccagacaactttgcttttgatcaaagagtctgagagaataggtattttaggcttg   639
                      **            *                * *      *      *

KIV-1_Exon1         ctctctttcctgagagtttgataaaatc---aggagaatatctggcggtggtgaggccac   695
KIV-2.1_Exon1       ctctccttcctcctagttc---tattgagcagaagggaaatctggaggtgaggagatcac   697
KIV-2.2_Exon1       ctctccttcctcctagttc---tattgagcagaagggaaatctggaggtgaggagatcac   697
KIV-2.3B_Exon1      ctctccttcctcctagttc---tattgagcagaagggaaatctggaggtgaggagatcac   697
KIV-2.4_Exon1       ctctccttcctcctagttc---tattgagcagaagggaaatctggaggtgaggagatcac   697
KIV-2.5_Exon1       ctctccttcctcctagttc---tattgagcagaagggaaatctggaggtgagaagatcac   697
KIV-2.6_Exon1       ctctccttcctcctagttc---tattgagcagaagggaaatctggaggtgaggagatcac   697
KIV-3_Exon1         ctcaccttcctcctagttt---tgtggagcagaagggaaatgaggaggcgaggagatcac   697
KIV-4_Exon1         ctctccttcctcctagttt---aatggagcagaaagg-tatctggaggcaaggatatcac   696
KIV-5_Exon1         ccaggctggagtgcagtggcatgatctcggctcactgcaagctccgcctcccgggttcac   700
KIV-6_Exon1         ctctcttttctcatagttgacagaag-c---aggaggatatttgaaggtggtgagttctc   694
KIV-7_Exon1         ctctcttttctcatagttgatagaag-t---aggaggatacttggaggtggtgaggtctc   695
KIV-8_Exon1         ctatctttcataacaatttgatggaagc---agcaggatatatggaggtggtgaagtctc   697
KIV-9_Exon1         ctatttttcctaatagttttatggaagg---agtagaatatacggaagtggcgaagtcat   695
KIV-10_Exon1        ctatcttttctaatagtctgatggaagc---agaaggctacatggagctgatgaggtctt   696
                    *     *       * *                      *                 *

KIV-1_Exon1         aataatggaaaatcagaatgttta----------------gacagagtcagcttcaacaa   739
KIV-2.1_Exon1       attatgaagaaagtcagaatgacaaaggaccagacacttagattaccc----ttccacaa   753
KIV-2.2_Exon1       attatgaagaaagtcagaatgacaaaggaccagacacttagattaccc----ttccacaa   753
KIV-2.3B_Exon1      attatgaagaaagtcagaatgacaaaggaccagacacttagattaccc----ttccacaa   753
KIV-2.4_Exon1       attatgaagaaagtcagaatgacaaaggaccagacacttagattaccc----ttccacaa   753
KIV-2.5_Exon1       attatgaagaaagtcagaatgacaaaggaccagacacttagattaccc----ttccacaa   753
```

```
KIV-2.6_Exon1       attatgaagaaagtcagaatgacaaaggaccagacacttagattaccc----ttccacaa     753
KIV-3_Exon1         cttatgaagaaagtcagaatgacgaaccaccaaacacttagattaccc----ttgcccaa     753
KIV-4_Exon1         attaagaaacaagtcaagatgacaaatgatgaaactcttagagtaccc----ttccacaa     752
KIV-5_Exon1         accattc------tcctgcttcagcctctctagcagctgggactacag----gcacccac     750
KIV-6_Exon1         atgaatagaaagctcaggaca---catggccacgtgcttagaaatagcaccattccacaa     751
KIV-7_Exon1         atgaatagaaagctcagaagaacatatgaccatgtgcttagaaatagcaccattccacaa     755
KIV-8_Exon1         attaatgtaaagctaaggagatcaaatgaccaaatgctgagacaaagtatcattccacaa     757
KIV-9_Exon1         attaatgtaaagctcagaagataaatgaccaaagcttaaacacagcacca--ttccacaa     753
KIV-10_Exon1        tttaatataaagctcaagagatcaaatgatcaaatacttagag--tgcca--ttctacaa     752
                       *                                     *               **

KIV-1_Exon1         cactcactaaaggtcaatgtg     760
KIV-2.1_Exon1       caccaac--------------     760
KIV-2.2_Exon1       caccaac--------------     760
KIV-2.3B_Exon1      caccaac--------------     760
KIV-2.4_Exon1       caccaac--------------     760
KIV-2.5_Exon1       caccaac--------------     760
KIV-2.6_Exon1       caccaac--------------     760
KIV-3_Exon1         cacccac--------------     760
KIV-4_Exon1         cacccact-------------     760
KIV-5_Exon1         caccacaccc-----------     760
KIV-6_Exon1         tgcccacta------------     760
KIV-7_Exon1         tgccc----------------     760
KIV-8_Exon1         tgc------------------     760
KIV-9_Exon1         tgcccac--------------     760
KIV-10_Exon1        ggctcata-------------     760
                      *
```
