# Supplementary material for: Resolving intra-repeat variation in medically relevant VNTRs from short-read sequencing data using the cardiovascular risk gene LPA as a model
Source: Genome Biol. 2024 Jun 26;25:167. doi: 10.1186/s13059-024-03316-5 (PMC11201333; doi:10.1186/s13059-024-03316-5)
Supplement: Supplementary file 6 — Additional file 6. Supplementary figures. [file 13059_2024_3316_MOESM6_ESM.pdf]

Supplementary Figures

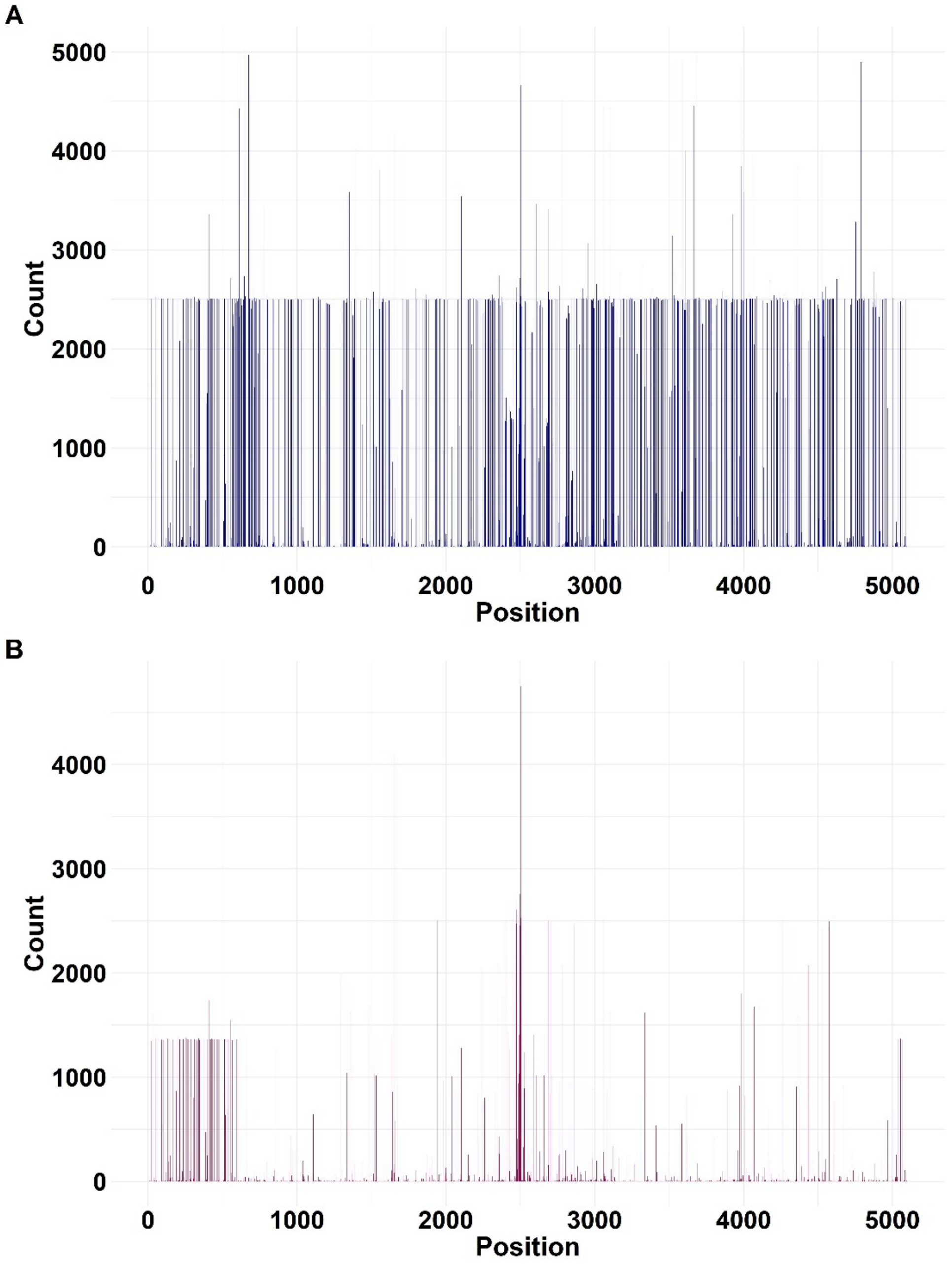

Fig *S*1 Variants detected in the KIV-2 VNTR in 2,504 individuals from 1000 Genome Project in whole-genome sequencing data. Positions are numbered as in Coassin & Schönherr et al, J Lipid Res 57 2019. **Panel A)** KIV-2 variant calling was performed extracting reads from one single region of interest (ROI) based on automatically defined start and end coordinates of the KIV-2 VNTR as in Mukamel et al. Science 373 2021. **Panel B)** KIV-2 variant calling was performed extracting reads from two alternative ROIs assigned in the signature-based approach for KIV-2 variant calling developed in the work at hand. The signature-based approach strongly reduces the noise compared to Panel A by minimizing the false positive calls in non KIV-2B samples.

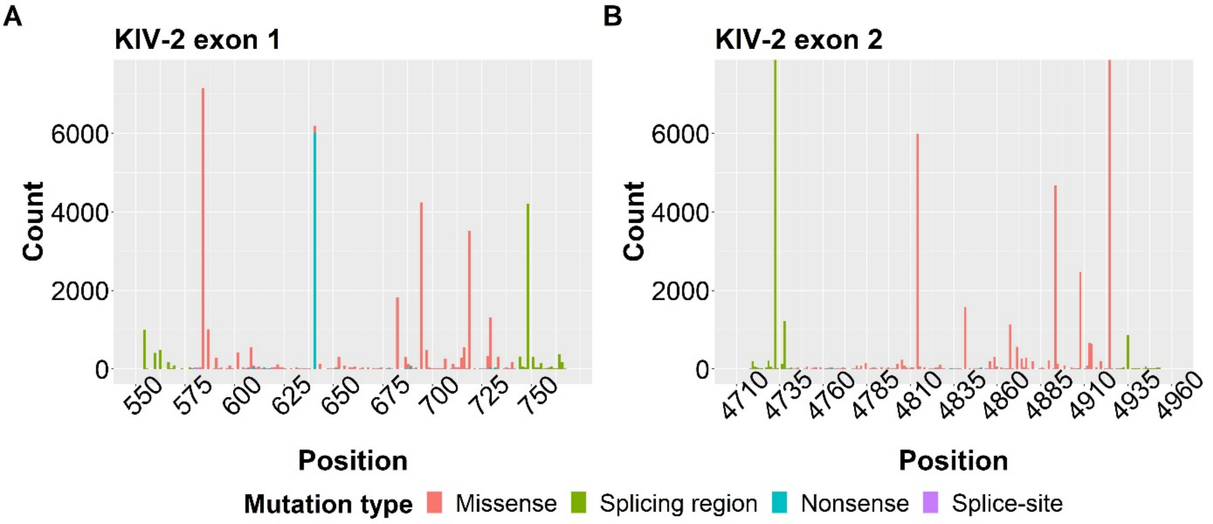

Fig S2 Absolute frequency of the carriers for each KIV-2 mutations detected at least once in KIV-2 exon 1 (**Panel A**) and 2 (**Panel B**). The mutations are filtered for missense, nonsense, splicesite mutations or mutations within the splicing region (as within the 25 bp upstream and downstream KIV-2 exon). The number of individuals carrying at least one of the above mutations in KIV-2 exon 1 and exon 2 are 36,699 and 94,758, respectively. Y-axis limit=7500.

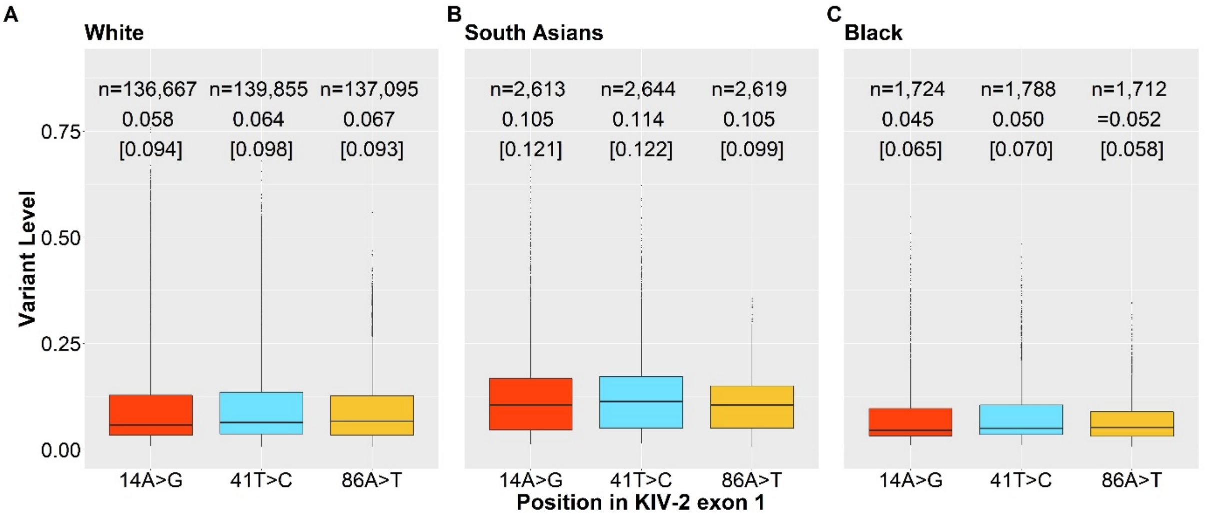

Fig S3 Distribution (given as median [interquantile range]) of the variant level for the KIV-2B canonical variants 14A>G, 41T>C and 86A>T in exon 1 detected in UK Biobank participants of White (Panel A) South Asians (Panel B) and Black ancestries (Panel C). n=number of carriers.
