# Supplementary material for: Resolving intra-repeat variation in medically relevant VNTRs from short-read sequencing data using the cardiovascular risk gene LPA as a model
Source: Genome Biol. 2024 Jun 26;25:167. doi: 10.1186/s13059-024-03316-5 (PMC11201333; doi:10.1186/s13059-024-03316-5)
Supplement: Supplementary file 7 — Additional file 7. Nextflow workflow report. [file 13059_2024_3316_MOESM7_ESM.html]

[distracted\_bohr] Nextflow Workflow Report


Nextflow Report


- Summary
- Resources
- Tasks

[distracted\_bohr]

# Nextflow workflow report

## `[distracted_bohr]`

Workflow execution completed successfully!

Run times
:   21-Feb-2024 11:20:39 - 21-Feb-2024 11:53:34
    (duration: **32m 55s**)

12522 succeeded

0 cached

0 ignored

0 failed

Nextflow command
:   ```
    nextflow run genepi/vntr-calling-nf -r v0.4.9 -profile singularity -c configs/1000g-wgs-lpa-signature.config -with-report 1000g-wgs-signature.html
    ```

CPU-Hours
:   `7.5`

Launch directory
:   `/home/q141ss/projects/2024-1000g-analysis-lpa`

Work directory
:   `/home/q141ss/projects/2024-1000g-analysis-lpa/work`

Project directory
:   `/home/q141ss/.nextflow/assets/genepi/vntr-calling-nf`

Script name
:   `main.nf`

Script ID
:   `40892a7ac01d5f41b4900da597afe937`

Workflow session
:   `ad239123-6235-4383-8c8d-87fa4b28418b`

Workflow repository
:   `https://github.com/genepi/vntr-calling-nf`, revision `v0.4.9` (commit hash `501969e3e0dca1776c631f7cd24fbf6984f813ce`)

Workflow profile
:   singularity

Workflow container
:   `quay.io/genepi/vntr-calling-nf:v0.4.9`

Container engine
:   `singularity`

Nextflow version
:   version 22.10.4, build 5836 (09-12-2022 09:58 UTC)

## Resource Usage

These plots give an overview of the distribution of resource usage for each process.

#### CPU

- Raw Usage
- % Allocated

#### Memory

- Physical (RAM)
- Virtual (RAM + Disk swap)
- % RAM Allocated

#### Job Duration

- Raw Usage
- % Allocated

#### I/O

- Read
- Write

## Tasks

This table shows information about each task in the workflow. Use the search box on the right
to filter rows for specific values. Clicking headers will sort the table by that value and
scrolling side to side will reveal more columns.

Values shown as:

Human readable
Raw values

(tasks table omitted because the dataset is too big)

Generated by Nextflow, version 22.10.4
