# Supplementary material for: Resolving intra-repeat variation in medically relevant VNTRs from short-read sequencing data using the cardiovascular risk gene LPA as a model
Source: Genome Biol. 2024 Jun 26;25:167. doi: 10.1186/s13059-024-03316-5 (PMC11201333; doi:10.1186/s13059-024-03316-5)
Supplement: Supplementary file 8 — Additional file 8. Supplementary note for Sanger Sequencing population. [file 13059_2024_3316_MOESM8_ESM.pdf]

Additional File 8

The Salzburg Atherosclerosis Prevention Program in subjects at High Individual Risk (SAPHIR) is an observational study involving healthy unrelated subjects: 645 females from 39 to 67 years of age and 1093 males from 39 to 66 years of age. Study participants were recruited by health screening programs in large companies in area of the city of Salzburg in Austria from 1999 to 2002. All individuals were of West-Eurasian origin. Informed consent was obtained from each participant. Details about the exclusion criteria and baseline assessment are described in detail elsewhere (Heid et al., Diabetes 55, 2006 and Coassin et al., Eur Heart J 38, 2017). Blood samples were collected after an overnight fasting period. Full Lp(a) phenotyping by ELISA (Lp(a) plasma concentration) and Western Blot (apolipoprotein(a) isoform sizes in plasma, reflecting the KIV-2 VNTR repeat number) was available for n=1,522 participants.
