# Supplementary material for: Resolving intra-repeat variation in medically relevant VNTRs from short-read sequencing data using the cardiovascular risk gene LPA as a model
Source: Genome Biol. 2024 Jun 26;25:167. doi: 10.1186/s13059-024-03316-5 (PMC11201333; doi:10.1186/s13059-024-03316-5)
Supplement: Supplementary file 9 — Additional file 9. Peer review history. [file 13059_2024_3316_MOESM9_ESM.docx]

**Review history**

**First round of review**

**Reviewer 1**

This manuscript describes a new pipeline for genotyping sequence variants within variable number tandem repeats (VNTRs) from short-read sequencing data. As the authors note, standard short-read analysis pipelines exclude such regions from analysis, such that additional method development in this area is needed (given the large amount of short-read sequencing data available from large biobank data sets, in contrast to very limited amounts of long-read sequencing data).

Overall, the authors' pipeline appears to be thoughtfully designed and implemented -- and carefully benchmarked using gold-standard LPA sequence data previously assembled by the authors -- such that it seems to be a promising addition to the present toolkit for repeat analysis. I have only a few minor questions and suggestions.

1. Based on the description of the pipeline, I expect its computational cost is probably quite small (even for hundreds of thousands of genomes/exomes), but some discussion of resource requirements would still be useful to potential users. Is the most computationally-intensive step the realignment? Is memory use low throughout the entire pipeline, or does the mutserve variant-calling step at the end need more memory (since it considers all samples at once)?

2. The identification of the KIV-2B signature sequence and its utility in variant-calling are interesting additional elements of the paper. That said, one question I am left wondering about is whether distinguishing whether variants belong in KIV-3 vs. KIV-2 is actually relevant for downstream modeling of Lp(a). That is, does it actually make a difference to know that some variants come from KIV-3 exon 1 vs. simply treating ROI-3 (= KIV-1 exon2, KIV-2, KIV-3 exon 1) as one big repeat and not worrying about exactly how to assign coding variants to specific kringles? (Commenting on this question would be useful even without any further analysis.)

3. The 1kG call set that the authors generated for 6 VNTRs sounds like a useful reference. I took a quick look at the GitHub link provided in the manuscript but couldn't immediately find the location of the call set. I assume it is there somewhere, but making the location more obvious would be hepful.

4. The specific task that the method is trying to accomplish could be clarified in some places, e.g., in the Abstract it is unclear whether "computational approach to resolve VNTRs" refers to complete assembly of alleles vs. estimation of repeat copy-numbers vs. identification of SNP/indel variants within the repeat.

5. The legend of Fig. 1A has a typo: "represented with 6 KIV-2 repeats, one being a KIV-2" -> should be "one being a KIV-2B."

6. In Fig. 5, ROI-3 and ROI-4 appear to be identical -- is this an error?

**Reviewer 2**

The paper by Maio et al. presents an approach to resolve medically relevant VNTRs from short-read sequencing data, and they directly apply it to the LPA gene and others. In the paper, Maio et al. show evidence that they carefully evaluated this approach to refine methods to identify variants from critical gene regions that cannot be adequately analyzed using traditional approaches.

Overall, I think this work will be a valuable addition to the field, but the paper itself needs substantial revisions to clarify the work. There are a lot of gaps in logic, and the methods are nowhere near sufficient for someone to reproduce the work. There are portions of the results I still don't know exactly how they obtained them.

Additionally, I was genuinely excited to read their results from applying it to the UK Biobank, but was left a bit underwhelmed. Maybe they have results that they're planning for another paper, which would make sense, but if not, it's a missed opportunity. I hope another paper is coming.

To the authors: this is great work. Please accept my feedback as a genuine attempt to provide constructive feedback. It is not meant to be negative towards the work or the authors. I don't perceive any fundamental flaws. The paper just needs work.

Specific feedback:

Minor
1. To me, this work is a clear, even if subtle, improvement upon previous methods described to rescue variants from dark regions resulting from repetitive or duplicated sequence (what we termed "camouflaged"). That said, I feel like it's important for the authors to clarify throughout the paper exactly how their approach improves upon previous methods and where it's building on previous methods. Specifically, my understanding is that they have refined our previous work (Ebbert et al.) by refining where to extract reads from, and automating it, but that the underlying approach is still the same. I have debated whether to include this point because it can come across self-serving, but I generally feel it's important to be clear about these things. This point has no bearing on the value of this work, however.
2. The sample selection for set a and b is a bit weird at first glance. After reading through the paper, I think I understand why, but authors should make it clear why it appears so imbalanced with 2B samples.
3. The results comparing ROIs isn't clear on how or why those specific ROIs were selected and there wasn't an obvious method for several of them by looking at the figure. After reading deeper into the paper, it became more evident to me that it's related to the similarities between exon 1 in 2B and 3. This needs to be much more clear throughout the paper, including Figure 5. Emphasizing those exons in the figure and explaining why they're special to the work (in main text and figure legend) would do wonders.
4. Unless I missed something, ROIs 3 & 4 are the same in Figure 5. Please either fix the figure or clarify in the text how they're different.
5. Would be helpful to readers to briefly that higher F1 is better when presenting the statistics. Kudos for explaining what an F1 is to begin with, though! Love it. Clarity and helping your reader along is wonderful.
6. Authors report some basic statistics regarding the correlation between the T/C alleles and KIV-2B status. Those are helpful, but authors need to report the actual LD values (r-squared and d-prime). This could be reported along with and based on the UK Biobank data since their initial cohort is relatively small.
7. Authors need to specifically mention in the following sentence that they used Sanger sequencing to distinguish between these exons. It's sort of mentioned in the methods, but it should be explicitly stated here: "We sequenced the KIV-3 region in 66 samples with known KIV-2 variants patterns (2) (50 KIV-2B samples and 16 non-KIV-2B) (see Additional File 3)."
8. Related, authors provide Sanger details in Supp table 16, including primer sequence, which is great. With this being a repetitive region, however, authors need to convince the reader that was there sufficient unique adjacent sequence to be confident they were sequencing the exon of interest. Is there unique sequence? How similar is that sequence to the next closest hit in hg38?
Major
1. I was surprised when authors stated that they're using the read coverage metric to determine 2B status, but what really surprised me was that they did not specify coverage of what, nor did they specify how coverage is a meaningful metric. They don't even seem to check if the reads have a T or C allele from the following sentence: "Using the available WES data from the non-KIV-2B samples (n=12), we calculated an empiric threshold to separate between the two different sample subtypes using the mean read coverage plus 2 SD (see Additional File 1-Supp. Table 6). This value has been used to dynamically assign the optimal ROI."
2. Authors state that one sample was misclassified because of a "rare" T/T homozygous genotype in the following sentence: "The reason for the single misclassification was a rare T/T homozygous genotype at the signature position in a non-KIV-2B individual. As a result, this sample was processed with ROI-8 despite being non KIV-2B." Please clarify why being homozygous for T explains the misclassification. This entire section was difficult to follow. Also, why is it rare? If all 2B individuals carry "at least" one T allele, that sounds like being homozygous wouldn't be especially rare. Please report the allele frequency in both data sets.
3. After reading the entire paper, I'm clueless how this is being done and how it is being automated: "For each VNTR, LPA included, we first tested ROIs spanning through the start and end coordinates generated by automatized tools (8) (see Methods)." Referencing another paper is not sufficient for this, and unless I misunderstood, this automation is supposed to be a key benefit from this paper. I think authors are referring to the methods section titled "Paralogous sequence variant identification", but it isn't clear. I also couldn't find anywhere in methods that specifies how ROI selection is automated.
4. The following sentences says to see the methods. I could not find this in the methods. "To exclude them from downstream analyses, we thus compiled a list of existing PSVs between the repeats for each investigated VNTR (see Methods) and estimated the expected conserved bases for each VNTR (see Table 3)."
5. As mentioned in passing, the methods need way more detail and should be easily linked to sections of the results. e.g., include text about how ROIs are selected and how it's automated.

As discussed, I think this is great work overall. Please improve the paper itself.

Best wishes.

Sincerely,

Mark T. W. Ebbert

**Reviewer 3**

“Resolving medically relevant VNTRs from short-read sequencing data using the cardiovascular risk gene LPA as a model” by Maio et al. describes a nextflow pipeline for targeted SNV calling in VNTRs. Calling variants in VNTRs is challenging due to high sequence homology between repeats. By extracting reads aligned to a targeted repeat region and realigning them to a reference motif the pipeline identifies low frequency variants present in one or more repeat copies. The pipeline is robust and straightforward to use. I was able to run the pipeline on my linux workstation with both the test dataset provided in the github repository as well as on a short-reads whole genome sequencing dataset I had on my system without error. By applying the pipeline, developed and validated for the KIV-2 repeat in the medically relevant LPA gene, to five other medically relevant genes with VNTRs the authors were able to generate a population dataset of variants in these VNTRs along with variants in LPA KIV-2 for for nearly 200,00 samples in the UK biobank. These population datasets will serve as a valuable resource for the community to analyze and characterize variants in these medically relevant genes.

Main Issues

- Introduction
  - Providing the KIV-s domain size in base pairs as well as information about LPA gene size and how this varies for the population would help readers conceptualize how challenging calling variants in VNTRs is.
  - “This dataset also served us as a gold standard to evaluate variant calling from whole-genome sequencing (WGS) or whole-exome sequencing (WES) data.” Please revise to clarify that this dataset is for evaluating variant calls in the KIV-2 region of the LPA VNTR.
  - Include a brief description of how the truth variant calls for Datasets A and B were determined to provide readers with confidence in the accuracy of the truth set without having to go to the citation unless they are interested in detailed description of the truth set generation.
  - The manuscript would benefit from a description of other VNTR variant callers (e.g. <https://genome.cshlp.org/content/33/4/511.full> and <https://www.ncbi.nlm.nih.gov/pmc/articles/PMC9379575/>) and how the method presented here differs from existing methods.
  - Please clarify that this method is SNP calling in targeted VNTRs using WGS or WES data. Specifically noting that the pipeline only calls variants in one VNTR at a time and does not call indels.
- Methods
  - Nextflow pipeline targeted snv calling in VNTRs
    - Please include a url for the pipeline github repo
    - Include bed and fasta files used for additional gene analysis in github repo with documentation / guidance on creating files for additional VNTRs.
    - Please provide additional details about the output tsv and vcf files, specifically how they are customizable and can be annotated.
    - Include metric definitions in documentation, noting when NAs are reported.
  - Figure 3. Is the subsequent variant annotation part of this workflow, if not please clarify.
  - “We applied the pipeline to 2,504 samples from 1kGP for 5 other protein-coding VNTRs showing VNTR units that are longer than the read length and include less than 50% PSV positions.” Unclear statement maybe use “with” instead of “showing”
  - Fig 4 - Datasets A and B not balanced for KIV-2B and non-KIV2B samples. Please note how were the samples assigned to dataset A or dataset B? Does the lack of balance bias the downstream analysis?
  - Figure 6 - signature-based strategy: Clarify in figure that reads from the full LPA region were used when searching for the signature sequence.
- Results
  - Unclear total number of variants for individual samples TP, FP, FN as the total number of variants seems small using F1, precision, sensitivity, and specificity performance metrics alone might be misleading at times. Good to include the raw variant numbers. Quantifying statements like “but with less false positive calls and therefore higher mean F1-score (dataset A experiment-1 and experiment-2: 81.9%, and 84.6%).” with changes to the number of false positives. This would provide readers with a clearer understanding of the improvements. The impact of different methods on the F1-scores are rather large at times and if the total number of variants is small only providing the the changes can be misleading.
  - Computational Workflow for variant calling in VNTRs
    - Please revise “We developed a complete computational workflow (see Figure 3) to detect variants in VNTRs at scale.” to clarify that this method is for calling variants in targeted/ specific VNTRs.
  - “The reason for the single misclassification was a rare T/T homozygous genotype at the signature position in a non-KIV-2B individual.” How rare is this mutation, is there population data to indicate how often this will cause a KIV-2 classification error?
  - “The final call set for each analyzed VNTR using the 1kG samples is provided at https://github.com/genepi/exome-vntr-nf and represents to the best of our knowledge the first fully resolved and computational reproducible version of these VNTRs.” I was unable to find these callsets in the repo.
  - Genomic samples and datasets - please include a description of the 66 sample dataset.
  - UK biobanks samples - comment on the ancestry grouping, any subgroup potential issues or biases from the groupings?
  - To support claims regarding the methods ability to call variants in VNTRs from WGS and WES data it would be useful to compare WGS and WES based variant calls for the same samples.
  - Include definitions for precision, sensitivity, and F1-score. If possible please use established standard definitions for these performance metrics, <https://www.nature.com/articles/s41587-019-0054-x>.
  - Supplementary Table 2: Precision values all 0, Sensitivity and F1-Score NA, this seems inconsistent with results in the manuscript.
  - Fig 1 - To help readers understand scale please include overall size (bp) as reference coordinates (probably just GRCh38 for simplicity) and potentially size population size range (average, min, max repeat copy numbers.)
  - Figure 3B “WGS or WES” instead of just WES to be consistent with manuscript text indicating this method can be used with WES and WGS data.
  - Figure 5 - annotate with region sizes
  - Figure 8 black lines difficult to see in the figure, initially it looked like a bar plot and not a boxplot.
    - Assuming colors used in the two plots represent corresponding groups, it would be useful to combine panels A and B with the SNP and WES boxplots next to eachother for each carrier status category making it easier for readers to directly compare values between variant calling methods.
  - Table 1: median (max - min) instead of mean +/- sd. Bold or italize ROI-* for emphasis
    - Maybe add columns with summary statistics for false positive counts for the non KIV-2B samples
  - Table 2 legend - explain NA values
  - Table 4: providing coordinates for the different regions with reference version information. Maybe summary stats for number of mutations per individual.
- Discussion
  - Modify language to state that this method is for targeted VNTR variant calling that was optimized and evaluated using the KIV-2 VNTR in the LPA gene.
  - For population variant analysis - carrier mutations present in one or a few individuals, these might be errors/ noise, might want to address in discussion
- Limitations
  - Please note that the variants are reported relative to the reference motif coordinates and not reference genome coordinates as is standard for vcf files. Your pipeline does this for good reason but important to highlight. I also suggest adding a statement that how to best represent variants in VNTRs and benchmarking these variants where performing whole genome variant calling is an open issue and will require changes to VCF file format specifications and development of new benchmarking tools.
- Supplemental Material
  - Supplemental file 4: Consider also providing as MSA text file, describe base highlighting, color, and asterisk in legend.
  - Supplemental Table 13: please define mean variant level
  - Supplemental Table 17: should the column header for D or E be hg38 coordinates?

Minor Edits

- Figure 1 panel B legend “The reference genome sequence shows 100% ~~of~~ identity between the exon 2 sequence of” first of not necessary.
- Figure 1 Panel C legend “KIV-2B and KIV-3 differ from the more frequent KIV-2A by the three PSV in ~~in~~ exon 1 at positions 14,” extra in
- Figure 6 panel b legend “redirected to the optimal ROI strategy for (step 3) KIV-2 read~~s~~ extraction.” reads should be singular.
- “Here, we tested three published regions that have been used o isolate KIV-2 reads from WES data already aligned to the reference genome” o should be to
- Supplemental Figure 1 legend “Panel A) KIV-2 variant calling was performed extracting reads from one ~~one~~ single region of interest (ROI)” extra one
- Discussion - “This suggests that using stringent coordinates for read~~s~~ extraction” reads should be singular.

**Authors’ response to reviewers**

# Reviewer 1

*This manuscript describes a new pipeline for genotyping sequence variants within variable number tandem repeats (VNTRs) from short-read sequencing data. As the authors note, standard short-read analysis pipelines exclude such regions from analysis, such that additional method development in this area is needed (given the large amount of short-read sequencing data available from large biobank data sets, in contrast to very limited amounts of long-read sequencing data).*

*Overall, the authors' pipeline appears to be thoughtfully designed and implemented -- and carefully benchmarked using gold-standard LPA sequence data previously assembled by the authors -- such that it seems to be a promising addition to the present toolkit for repeat analysis. I have only a few minor questions and suggestions.*

We thank the reviewer for the time, the positive feedback and the helpful comments. We are confident that our *LPA* KIV-2 gold-standard experiments in combination with the provision of a scalable and reproducible computational workflow constitute an improvement to the field and help resolving variants within VNTR regions in a straightforward way. Your feedback helped us to improve the manuscript and to add additional or missing information. As suggested by another reviewer, we also added additional UK Biobank analysis, which helped us identify variants within the KIV-2 region associated with Lp(a) concentrations and cardiovascular disease, therefore	showing	the	benefit	of	our	approach. All major changes to the manuscript have been highlighted in yellow in the track change manuscript and are copied into this document with the corresponding page number. We also provide a clean version of the manuscript.

*1. Based on the description of the pipeline, I expect its computational cost is probably quite small (even for hundreds of thousands of genomes/exomes), but some discussion of resource requirements would still be useful to potential users. Is the most computationally-intensive step*

*the realignment? Is memory use low throughout the entire pipeline, or does the mutserve variant-calling step at the end need more memory (since it considers all samples at once)?*

We thank the reviewer for highlighting this important point. To analyze CPU and memory consumption, we have generated a Nextflow execution report, offering a range of valuable metrics regarding the workflow execution itself. As expected, memory consumption was low for all steps but showed an increase in memory (5GB for 1000 Genomes Phase 3 data, n=2,504). After carefully evaluating the report, we decided to improve the parallelization and memory consumption even further: We are now executing the variant calling step on each sample separately, which helps to utilize large clusters and to keep the memory low. Additionally, we implemented a new low-memory merging step of sample-specific variant calling results. Both changes have been integrated in v0.4.9 of our VNTR pipeline (https://github.com/genepi/vntr- calling-nf/releases/tag/v0.4.9). Applying these improvements, we were able to run the 1000 Genomes data in 33 min on our Slurm cluster with a memory of 1GB for each process (total CPU hours: 7.9). We added the Nextflow execution report including the CPU and memory consumption as Supplementary Data (Additional File 7). We added the following sentence to the manuscript in the Results section on page 10:

“As a technical note, we were able to run the pipeline on the 1000 Genomes Project data in 33 min (total CPU hours: 7.9) assigning a memory requirement of 1GB to each process (see Additional File 7).”

*2. The identification of the KIV-2B signature sequence and its utility in variant-calling are interesting additional elements of the paper. That said, one question I am left wondering about is whether distinguishing whether variants belong in KIV-3 vs. KIV-2 is actually relevant for downstream modeling of Lp(a). That is, does it actually make a difference to know that some variants come from KIV-3 exon 1 vs. simply treating ROI-3 (= KIV-1 exon2, KIV-2, KIV-3 exon 1) as one big repeat and not worrying about exactly how to assign coding variants to specific kringles? (Commenting on this question would be useful even without any further analysis.)*

This addresses an important point of the manuscript. Indeed, there are probably multiple perspectives possible on this. From a purely genomics perspective the differences might seem

minor and an approach like proposed by the reviewer is surely valid. On the other hand, there might be slight but relevant differences, if the topic is approached from a more mechanistic perspective and, especially, for target audiences who are oriented more towards Lp(a) research.

The structure-function relationships of the apolipoprotein(a) protein, the protein coded by *LPA*, are largely unknown. However, there have been single descriptions about factors binding specifically to the KIV-2 repeat loops like beta-2 glycoprotein I (i.e. APOH; Köchl et al, 1997, PMID: 9269765; the regulatory role of that protein has been supported recently also by GWAS in Hoekstra et al, 2021, PMID: 33115273 and Said et al, 2021, PMID: 33730874). The reason why apoH binds specifically to the KIV-2 domains are unknown, but might reside in the few different amino acids that differentiate the KIV-2 from other similar kringles or even in the overall particle structure. As all Lp(a) research follows this domain naming convention, the inclusion of the first exon of KIV-3 in mutation analyses would create spurious KIV-2 mutations that may confuse more biochemically oriented pipeline users or readers. Moreover, while the VNTR could be seen as extending into the first exon of KIV-3, it has to be considered that both KIV-3 exons are indeed covered in genetic studies like GWAS (due to still sufficient intronic differences that allow read mapping and often genotyping probe design). Including the KIV-3 into the repeat region might thus create additional confusion, as KIV-3 SNPs would additionally show up in the KIV-2 data. This might bring noise into studies searching for causal functional variants like done in Coassin et al, Eur Heart J 38, 2017, Schachtl-Riess et al, J Am Coll Cardiol 78, 2021 or Mukamel et al, Science 373, 2021. Indeed, these Lp(a)-specific considerations were also one of our major motivators when we first embarked in the project described within this manuscript. The strict separation between KIV-3 and KIV-2 also has practical implications. A major advantage of our project was the availability of reference data for true KIV-2 SNPs created by a highly specific PCR protocol that indeed amplifies only the KIV-2 domains and is able to exclude the similar KIV-1 and KIV-3 regions. These amplicons had been specifically designed based on the considerations above. Including the fixed flanking exons would also include KIV- 3 and KIV-1 SNPs into the KIV-2 sequencings and counteract the value of this highly specific reference dataset (albeit we acknowledge that this reference dataset has been indeed based on	the	considerations	above). The updated manuscript does not include a specific paragraph addressing this point for the sake of conciseness but we improved the motivation and overall readability of the manuscript at several locations.

*3. The 1kG call set that the authors generated for 6 VNTRs sounds like a useful reference. I took a quick look at the GitHub link provided in the manuscript but couldn't immediately find the location of the call set. I assume it is there somewhere, but making the location more obvious would be hepful.*

We thank the reviewer for raising this point and double checking the repository. We have now added a new part to the repository (https://github.com/genepi/vntr-calling- nf/tree/main/paper_analysis) including (a) scripts to initially download the 1000 Genomes Phase 3 data, (b) the Nextflow configuration files to run the pipeline and (c) the final VNTR variant results as requested by the reviewer. This part is also linked in the overview README file.

In the manuscript, we updated the following sentence in the Results section on page 9:

“The final call set for each analyzed VNTR using the 1kG samples is provided at the pipeline repository (see https://github.com/genepi/vntr-calling-nf) and represents the fully resolved call set of variants within the six analyzed VNTRs.”

*4. The specific task that the method is trying to accomplish could be clarified in some places, e.g., in the Abstract it is unclear whether "computational approach to resolve VNTRs" refers to complete assembly of alleles vs. estimation of repeat copy-numbers vs. identification of SNP/indel variants within the repeat.*

This is a well-taken point. We have now followed your suggestion, changing the wording in several sentences of the manuscript to better clarify the task of our pipeline. Prominent changes are especially at the following positions:

1. Title:

“Resolving variants in medically relevant VNTRs from short-read sequencing data using the cardiovascular risk gene *LPA* as a model.”

2. Abstract:

“Using the highly complex *LPA* gene as a model, we developed a computational

approach to resolve intra-repeat variation in VNTRs from largely available short-read sequencing data.”

3. Results, page 4:

*“*We present a computational approach to resolve intra-repeat variation in VNTRs from short-read sequencing data, and […]”.

4. Results, page 9:

“The final call set for each analyzed VNTR using the 1kG samples is provided at the pipeline repository (see https://github.com/genepi/vntr-calling-nf) and represents the fully resolved call set of variants within the six analyzed VNTRs.”

*5. The legend of Fig. 1A has a typo: "represented with 6 KIV-2 repeats, one being a KIV-2" -> should be "one being a KIV-2B."*

We thank the reviewer for pointing out this typo, which is now corrected in the manuscript.

*6. In Fig. 5, ROI-3 and ROI-4 appear to be identical -- is this an error?*

We thank the reviewer for pointing this out. We have now changed the figure, which was an inaccuracy on our side: the main difference between ROI-3 and ROI-4 is that ROI-3 starts from KIV-1 exon 2, while ROI-4 includes the intronic bases between KIV-1 exon 1 and exon 2. Since most intronic bases are outside the target region of our pipeline (exon +/-100bp), we originally thought that not providing details in this figure about the intronic bases might facilitate the interpretation. However, we are thankful for your comment, which made us realize that excluding the intronic bases ROI-3 and ROI-4 ended up looking exactly the same. Thus, we changed Figure 5 including as follows:

- ROI-2 start: after KIV-1 exon 1, keeping intronic bases
- ROI-2 end: for the same reason outlined above, the figure represented ROI-2 end exactly as ROI-8 and ROI-9 end. However, these two ROIs include more intronic bases; with this new figure, the difference is more visible

-	ROI-4 start: after KIV-1 exon 1, keeping intronic bases.

Reviewer 2

*The paper by Maio et al. presents an approach to resolve medically relevant VNTRs from short- read sequencing data, and they directly apply it to the LPA gene and others. In the paper, Maio et al. show evidence that they carefully evaluated this approach to refine methods to identify variants from critical gene regions that cannot be adequately analyzed using traditional approaches.*

*Overall, I think this work will be a valuable addition to the field, but the paper itself needs substantial revisions to clarify the work. There are a lot of gaps in logic, and the methods are nowhere near sufficient for someone to reproduce the work. There are portions of the results I still don't know exactly how they obtained them.*

*Additionally, I was genuinely excited to read their results from applying it to the UK Biobank, but was left a bit underwhelmed. Maybe they have results that they're planning for another paper, which would make sense, but if not, it's a missed opportunity. I hope another paper is coming.*

*To the authors: this is great work. Please accept my feedback as a genuine attempt to provide constructive feedback. It is not meant to be negative towards the work or the authors. I don't perceive any fundamental flaws. The paper just needs work.*

Dear Dr. Ebbert,

We appreciate your extensive and very constructive feedback. Reading our own manuscript with some distance, we fully acknowledge your points regarding the missing clarity and gaps in logic. We also want to make clear that reading your previous work encouraged us a lot to connect the dots and combine it with our own ideas from our Lp(a) research in the last decade (see also our later replies). We hope that our responses and the changes in the manuscript (including also citing previous work in this field more clearly) have improved the manuscript.

We especially share your excitement regarding UK Biobank and are indeed preparing a manuscript in this direction. However, we also recognized that this dampens some of the excitement about the manuscript at hand. Therefore, we have decided to include first UKB results also into this manuscript. Major changes in the manuscript are highlighted in yellow in the track change manuscript and are copied into this document with the corresponding page

number. We also provide a clean version of the manuscript.

The new paragraphs and figures regarding UK Biobank analysis are added at the following positions:

1. Results, page 11:

“We identified 9 SNPs significantly associated with decreased Lp(a) concentration (p value<5*10 8) in UKB participants of White ancestry with carrier frequency >0.015. These SNPs included the previously identified R21X (13) (position 640), 4733G>A (14) and 4925G>A (12), and 6 further SNPs across the KIV-2 VNTR (positions 515A>G, 516A>G, 584C>T, 4672A>G, 4698C>A, 5013G>T in this reference sequence (2, 25)). Median Lp(a) concentrations decreased with increasing number of effect alleles from

37.1 nmol/L [IQR=118.4 nmol/L; n=52,517] in the reference group (only reference alleles) to 4.7 nmol/L [8.6 nmol/L] in carriers of 5 Lp(a)-lowering alleles (n=400) and even 1.6 nmol/L [IQR=1.9 nmol/L] in 17 carriers of 6 Lp(a)-lowering alleles (Figure 9A). These SNPs also showed a rather additive protective effect against CAD. Compared to non-carriers (n=56,548, CAD cases=4,354), the Hazard Ratio (HR) for incident coronary artery disease (CAD) progressively decreases with increasing number of Lp(a)-lowering alleles. 4 Lp(a) lowering SNPs (n=3,767, CAD cases=1,413), decreased the CAD risk by ≈19% compared to non-carriers (HR=0.81, 95% confidence interval [CI]=0.72-0.93], p value=0.0016) (Figure 9B).”

2. Discussion, page 15:

“This also revealed new associations between SNPs hidden in the KIV-2 and reduced Lp(a) concentrations.”

3. Methods, page 19:

“For all detected KIV-2 SNPs, we tested the impact on Lp(a) concentration with linear regression in UKB individuals of White ancestry with available Lp(a) concentrations. We used Cox regression analysis (R package survival) with age as time scale and adjusted for sex to investigate the risk for incident CAD associated with the increasing number of Lp(a) lowering SNPs (carrier frequency >0.015) per individual. CAD events were defined using ICD10-codes I21-I25, as previously done (14, 42).”

4. Figure 9, Caption:

“Impact of 9 Lp(a)-lowering SNPs identified within the KIV-2 VNTR on Lp(a) concentrations and CAD risk in 186,607 UK Biobank participants of White ancestry with available WES data. A) Distribution of the Lp(a) concentration in individuals with 0 to 6

of the Lp(a) lowering SNPs. The Lp(a) concentration was available for 174,359 individuals. B) Hazard Ratio (HR) for the risk for coronary artery disease (CAD) associated with individuals carrying 0 to 6 of the Lp(a) lowering SNPs. Total CAD cases=13,363.”

5. Abstract:

“This approach successfully revealed new strong Lp(a) lowering effects for KIV-2 variants, with protective effect against coronary artery disease, and also validated previous findings based on tagging SNPs.”

Minor

*1. To me, this work is a clear, even if subtle, improvement upon previous methods described to rescue variants from dark regions resulting from repetitive or duplicated sequence (what we termed "camouflaged"). That said, I feel like it's important for the authors to clarify throughout the paper exactly how their approach improves upon previous methods and where it's building on previous methods. Specifically, my understanding is that they have refined our previous work (Ebbert et al.) by refining where to extract reads from, and automating it, but that the underlying approach is still the same. I have debated whether to include this point because it can come across self-serving, but I generally feel it's important to be clear about these things. This point has no bearing on the value of this work, however.*

We thank you for raising this point. As mentioned in our general reply, we really acknowledge your significant contributions to the field of VNTRs and we apologize for not being clear enough on this aspect. We critically revised our manuscript and tried to clarify better the contributions of others (including Ebbert et al.) to our work. Among other revised points in the introduction and results section, we have also added a substantial paragraph in the discussion at page 15 (highlighted in yellow), where this point is further addressed. In this point-by-point response, we would also like to briefly explain the etiology of the present project and approaches to clarify better the overlaps and differences to Ebbert et al.

Originally, our approach had been developed in parallel to the work presented in Ebbert et al. 2019. Our research background is genetic research on Lipoprotein(a), the *LPA* gene and the KIV-2 repeat, where our group has published several studies in the last years dealing with the

complex KIV-2 VNTR (such as Coassin et al, Eur Heart J 38, 2017; Coassin & Schönherr, J Lipid Res 57, 2019; Di Maio et al, Genome Med 12, 2020; Grüneis et al, Atherosclerosis 349, 2023; Grüneis et al, J Lipid Res 63, 2023, Coassin & Kronenberg, Atherosclerosis 349, 2023 and Amstler et al., bioRxiv, 03.01.582741, 2024).

Within our narrow field of Lipoprotein(a)/*LPA* genetics, the approach of mapping all reads of the KIV-2 VNTR to one single KIV-2 repeat and detect variants as low-level mutations has been actually described already in 1997 by Rosby et al. in Clin Genet 52. As such, the approach, which Rosby et al had termed as “batch sequencing”, had become quite widely appreciated in the field of *LPA* genetics. However, its success has been long limited by the use of Sanger sequencing and also later work separating the two *LPA* alleles by PFGE to improve sensitivity (Noureen et al, Plos One 10, 2015), had only limited success. In our works in 2017 and 2019 we then transferred “KIV-2 batch sequencing” to NGS mutation detection (using specific variant callers originally developed for mitochondrial heteroplasmy detection at our institute). About at the same time, we started investigating the use of WES and WGS data instead of targeted PCR amplification, encountering the issues described in our manuscript. These were primarily related to the distinction between the homologous KIV-3 and KIV-1 domains, as they are defined within the Lp(a) field (see also our reply to comment 2 of Reviewer 1). We thus set out to improve this for our own research purposes, which resulted in the work at hands.

During these works we were then very excited by seeing the study of Ebbert et al, 2019, which independently conceived an “*LPA* batch sequencing”-like approach and indeed succeeded in generalizing it also to multiple, respectively all VNTRs in the human genome. We were intrigued by seeing that the KIV-2 VNTR definition was sequence-based and not domain based, including so the flanking non-repetitive KIV-3 and KIV-1 exons into the VNTR definition. Coming from the Lp(a)-field this concept was new to us and we then included these considerations into our work to explore its effects on variant *LPA* KIV-2 calling, as we had already started investigating the effects of the flanking homologies also by ourselves. We were also intrigued and inspired by seeing that the “KIV-2 batch sequencing” concept had been generalized to other VNTRs

We fully acknowledge that the work of Ebbert et al, 2019 was published first. Therefore, we fully agree that the contributions of Ebbert et al. to our consideration deserve more space.

While connecting the dots between Ebbert et al. and our own work, we were also excited to see

the work by Mukamel et al. (Science 373, 2021), where VNTRs in relation to phenotypes have been analyzed in depth. The publication also included a list of *automatically* detected VNTRs which we used to see the applicability of our pipeline, originally developed for the *LPA* locus, to other VNTRs.

We think that our work builds on outstanding publications which we highly appreciate but also offers additional and unique benefits and features to the community, which stem from our in- depth experience in the Lp(a) field (e.g. Identification of subtypes especially the KIV-2B subtype using a SNP outside the VNTR, application to large datasets such as UK Biobank, automated Nextflow DSL2 pipeline for reproducibility and application to other VNTRs).

*2. The sample selection for set a and b is a bit weird at first glance. After reading through the paper, I think I understand why, but authors should make it clear why it appears so imbalanced with 2B samples.*

We appreciate this comment. As the reviewer says, sample selection in dataset A and B appears imbalanced regarding the presence/absence of KIV-2B subtypes. Both datasets A and B include only samples with known KIV-2 variants patterns from our previous work (Coassin & Schönherr, J Lipid Research 57, 2019), which was conducted in samples of European ancestry and reported KIV-2B subtypes in about 80% of the samples. To facilitate the investigation of strategies to separate between samples with and without KIV-2B subtypes, we arbitrarily enriched dataset B with *non KIV-2B samples*.

We have now added a sentence to the manuscript in the Results section, page 5:

“In particular, the enrichment for non KIV-2B samples in dataset B allowed the investigation of strategies to account for the presence/absence of KIV-2B subtypes in the variant calling workflow for the KIV-2 VNTR.”

*3. The results comparing ROIs isn't clear on how or why those specific ROIs were selected and there wasn't an obvious method for several of them by looking at the figure. After reading deeper into the paper, it became more evident to me that it's related to the similarities between exon 1 in 2B and 3. This needs to be much more clear throughout the paper, including Figure 5.*

*Emphasizing those exons in the figure and explaining why they're special to the work (in main text and figure legend) would do wonders.*

We apologize for not having been clearer on this critical aspect of the paper. We have critically reviewed our manuscript in this regard and now address this topic at multiple locations:

1) The Introduction was expanded (page 2) and now reads as follows:

“The exons of the KIV-2 present up to 98% base identity to the respective exons of the flanking KIV units, especially KIV-1 and KIV 3 (2). The second exon of KIV-1 is identical to the second KIV-2 exon and ≈70% homology is observed even within the first 200 intronic bases flanking the exons. This complicates the definition of the VNTR boundaries, read alignment and mutation detection. Moreover, the KIV-2 domain exists in at least three nearly identical subtypes (denoted by KIV-2A, B and C), which differ in the haplotype of three synonymous variants, as well as of >100 intronic variants(11) (see Figure 1C). The first exon of KIV-2B is identical to the first exon of KIV-3, while the respective second exons show 96% base identity.”

2) Figure 5 has been edited to use a color code showing the homologous/identical exons as proposed by the reviewer. This color code was previously used already in Figure 1 and we fully agree that it would be very helpful also in Figure 5 showing the ROIs. To reinforce this aspect even more we added a reference to the introduction in the legend of Figure 1 (“*see the Introduction for more explanation of these homologies*”) and modified the caption of Figure 5:

Caption: “**Figure 5:** Region of Interest (ROI) for KIV-2 read extraction from aligned WES data. The *LPA* gene is represented from KIV-1 to KIV-3 in transcription direction and not at scale for clarity. The KIV-2 VNTR (~~green~~) is represented with 6 repeats as in the reference genomes hg19 and hg38, with the third repeat in transcription direction being a KIV-2B unit. The other KIV-2 units are KIV-2A (in each unit, the first exon is represented in yellow, the second exon in blue). The KIV-2B unit in the VNTR is highly homologous to the KIV-3, which flanks the KIV-2 VNTR, showing the same first exon (purple) and 96% homology in the second exon. Additionally, all second KIV-2 exons are identical to the second KIV-1 exon (blue, see also the supplementary figure 12 of ref (2) for a detailed per-base identity matrix). In the first 200 intronic bp flanking each exon, 70-100% per-base identity is observed between KIV-1, KIV-2, KIV-2B and KIV-3. Thus, minor differences in the ROI used for read extraction, can extract highly

homologous reads that create spurious variants if aligned to only one repeat as done in this variant calling pipeline.

ROI-1 extracts reads from the complete *LPA* region (from KIV-1 to the protease domain, represented by an arrow). ROI-2 to ROI-9 progressively restricts the extraction region to the KIV-2 VNTR only. Since the KIV-2B appears fixed in the human genome reference, ROI-2 (previously published in(3)) and ROI-5 do not extract reads mapped to the KIV-2.2 exon 2 and KIV-2.3 subtype B, while ROI-6 and ROI-9 do not extract reads mapped to the KIV-2.3 subtype B. In combination with presence or absence of KIV-2B units in the sequenced sample, this creates different spurious variant calls in a sample- specific manner. The size of the regions enclosed by each ROI is provided as bp between start and end coordinates on hg38. Precise coordinates are provided in Additional File 1-Supp. Table 3.”

3) In Results section, page 5, where we first discuss the ROI experiments, we added the following explanations:

“This suggested that it would be necessary to restrict read extraction to the KIV-2 VNTR. However, the KIV-2 VNTR resembles a degenerated VNTR array, with a rather conserved core VNTR region (the KIV-2 domains), flanked by highly similar domains (KIV-1 and KIV-3). Additional complexity is added by the facts, that the exons of the KIV- 2B units (which are located within the KIV-2 VNTR array) are identical to the KIV-3 (which flanks the VNTR array) and that KIV-2B units are not present in all human individuals. This has led to varying definitions of the VNTR region across literature (2, 3, 8, 25). We therefore systematically assessed the KIV-2 variant calling performance of 9 different regions-of-interest to extract KIV-2 reads (ROI-1 to ROI-9) using both experiments of dataset A (see Figure 5). The ROIs where selected either from our own previous research (2), from literature (3, 8), or represented various curated combinations thereof (see Additional File 1-Supp. Table 3). The combinations where designed manually taking into considerations the various homologies described in the introduction and resulting possible read mismappings (see below).”

4) In Results section, page 6, we added the following explanations:

“Thus, the KIV-2B repeat subtype erroneously appears fixed and in single copy, while at population level the intra-individual frequency of KIV-2B units changes widely and even many “non KIV-2B individuals” exist, which carry zero KIV-2B repeat units (2, 9, 25). We noticed that ROI-8 performed best only in KIV-2B individuals with a F1 between

94.2-95.0% for both experiments. In non KIV-2B individuals, the mean F1 dropped to 45.0% for experiment-1 and to 52.3% for experiment-2 and resulted in a high number of false positive variants (see Table 2). Compared to ROI-8, ROI-9 does not extract reads mapped to the first exon of the KIV-2B repeat. Since no true KIV-2B reads exist in non KIV-2B individuals, the reads that are aligned in these individuals to the KIV-2B repeat of the reference genome must originate from highly homologous domains, most likely from KIV-3, which is nearly identical to the KIV-2B. Thus, in non KIV-2B individuals, ROI- 9 skips the KIV-2B domain in the reference during read extraction and so reduces false positive calls. ROI-9 improved the F1 in non KIV-2B individuals to 95.5% and to 100% in the two sequencing experiments in dataset A (see Discussion). We thus investigated strategies to minimize the false positive rate by accounting for the presence/absence of KIV-2B subtypes in a dynamic way within the Nextflow analysis pipeline.”

*4. Unless I missed something, ROIs 3 & 4 are the same in Figure 5. Please either fix the figure or clarify in the text how they're different.*

We thank the reviewer for pointing this out. We have now changed the figure, which was an inaccuracy on our side: the main difference between ROI-3 and ROI-4 is that ROI-3 starts from KIV-1 exon 2, while ROI-4 includes the intronic bases between KIV-1 exon 1 and exon 2. Since most intronic bases are outside the target region of our pipeline (exon +/-100bp), we originally thought that not providing details in this figure about the intronic bases might facilitate the interpretation. However, we are thankful for your comment, which made us realize that excluding the intronic bases ROI-3 and ROI-4 ended up looking exactly the same. Thus, we changed figure 5 including as follows:

- ROI-2 start: after KIV-1 exon 1, keeping intronic bases
- ROI-2 end: for the same reason outlined above, the figure represented ROI-2 end exactly as ROI-8 and ROI-9 end. However, these two ROIs include more intronic bases; with this new figure, the difference is more visible
- ROI-4 start: after KIV-1 exon 1, keeping intronic bases.

*5. Would be helpful to readers to briefly that higher F1 is better when presenting the statistics.*

*Kudos for explaining what an F1 is to begin with, though! Love it. Clarity and helping your reader along is wonderful.*

We thank the reviewer for the feedback and suggestions. To further clarify the meaning of the F1 score, we included a short sentence in the Results section on page 6 and, as also suggested by reviewer 3, we now also provide the definition for precision and sensitivity.

“Variations in the extraction region impacted the F1-score (F1), which describes the harmonic mean of precision (true positives/(true positives+false positives)) and sensitivity (true positives/(true positives+false negatives)) (26). Thus, F1 reflects both false negative and the false positive calls and performs well in unbalanced datasets. Higher F1 score indicates better performance.”

*6. Authors report some basic statistics regarding the correlation between the T/C alleles and KIV-2B status. Those are helpful, but authors need to report the actual LD values (r-squared and d-prime). This could be reported along with and based on the UK Biobank data since their initial cohort is relatively small.*

We thank the reviewer for this comment. Initially, we did not report LD values between the T/C alleles and KIV-2B status in the manuscript because of technical challenges of calculating precise LD values between KIV-2 variants and variants in the non-repetitive portion of *LPA*. While the T/C allele in KIV-3 (exon 1 position 86) is available as genotype obtained with Sanger- sequencing, the KIV-2B status can be reported only as carrier status, as no phasing of multiple occurrences of a mutant allele is possible. Deep sequencing thus provides “only” the carrier status, not a true genotype. Even more, the KIV-2B carrier status is defined based on the carrier status of the three canonical KIV-2B variants in KIV-2B exon 1, position 14, 41 and 86 (positions 594, 621 and 666 in our KIV-2 reference sequence). This complicates the calculation of LD values, which are usually based on genotypes.

However, we agree that including LD values can further support the signature-sequence and we have approximated them in the 66 samples with available KIV-2B carrier status (from our previous deep-sequencing approach from Coassin & Schönherr, J Lipid Res 57, 2019) We have encoded the KIV-2 position 594 (KIV-2 exon 1 position 14; as proxy for the other two canonical KIV-2B variants) as either wild-type or heterozygous (A/A=16 and A/G=50). Since the signature

sequence is based on the presence/absence of the T allele at position 86 in exon 1 of KIV-3, we had two options to encode the genotype of this variant:

1) Option 1: C/C=11, T/C=31 and T/T=24. In this case, all heterozygous individuals (29 T/C and 2 T/A) were encoded as T/C.
2) Option 2: C/C=11, T/C=55. In this case, all heterozygous and homozygous for the mutations were encoded as T/C. This reflects a “carrier-status encoding”, as for the KIV- 2 variants, as explained above.

For the KIV-2 variant, all carriers are encoded as heterozygous and there are no individuals encoded as homozygous.

We used of the R package *genetics* to calculate the LD between KIV-2 exon 1 position 14 and KIV-3 exon 1 position 86 and obtained:

1) Option 1:

-	d-prime=0.9995701

-	r-squared=0.41.

2) Option 2:

-	d-prime=0.9997041

-	r-squared=0.85 Interpretation:

In option 1, the LD is calculated with the genotype of the signature position (KIV-3, exon 1 position 86). This situation resembles a LD between two SNPs with highly different MAF and therefore D´ does better reflect the LD between these two variants compared to R-squared (which would be only ~0.41).

In option 2, also the KIV-3 variant is encoded as either wild-type or heterozygous, which resembles a carrier status-like encoding (respectively presence/absence). Thus, the unbalanced frequency of option 1 is partially resolved and R-squared is ~0.85.

In both cases, the D´ is very high (~0.99), indicating the absence of recombination between the T allele in the signature position (KIV-3, exon 1 position 86) and the presence of KIV-2B repeats.

Based on these considerations, we have now provided a Supplementary Note in Additional File 4 to the paper, with detailed explanation of the LD calculation, and have included the information concerning d-prime index at page 7:

“Accordingly, we observed a strong LD between the bases at KIV-2 position 594 (used as proxy for KIV-2B absence/presence) and position 86 in KIV-3 exon 1 (Lewontin´s D´=0.9995; indicating absence of recombination between the loci). Detailed explanation about LD investigation for KIV-2 SNPs is provided as Supplementary Note in Additional File 4).”

We also would like to explain why the calculation of LD values was only possible in these 66 samples. As mentioned above, these samples have available a true KIV-2B carrier status from our previous deep-sequencing approach (Coassin & Schönherr et al., J Lipid Res 57, 2019) and KIV-3 exon 1 position 86 genotype (from Sanger sequencing). In UK Biobank and 1000G data, the KIV-2B carrier status can only be obtained using the our signature approach, as the short-read WES data of UKB inevitably presents also the KIV-2B/KIV-3 ambiguity that we wanted to resolve with the signature sequence approach. Thus, calculating the LD in UKB would be biased by our own assumptions and approach.

*7. Authors need to specifically mention in the following sentence that they used Sanger sequencing to distinguish between these exons. It's sort of mentioned in the methods, but it should be explicitly stated here: "We sequenced the KIV-3 region in 66 samples with known KIV-2 variants patterns (2) (50 KIV-2B samples and 16 non-KIV-2B) (see Additional File 3)."*

Thank you for raising this point! The sentence was edited to improve the clarity and was also expanded with a reference to the new Supplementary note (see also next reviewer point).

The sentence (page 7) now reads as follows:

“To this end, we sequenced the KIV-3 region in 66 samples with known KIV-2 variants patterns

(2) using a Sanger sequencing protocol designed to differentiate KIV-3 from KIV-2 and KIV-2B, as well as other KIV domains (50 KIV-2B samples and 16 non-KIV-2B) (see Additional File 2; for considerations regarding the specificity of the KIV-3 PCR and Sanger sequencing see Additional File 3).”

*8. Related, authors provide Sanger details in Supp table 16, including primer sequence, which*

*is great. With this being a repetitive region, however, authors need to convince the reader that was there sufficient unique adjacent sequence to be confident they were sequencing the exon of interest. Is there unique sequence? How similar is that sequence to the next closest hit in hg38?*

This is an important point. The specificity can be evaluated both on the primer design side and by inspecting the Sanger sequencing traces for “contaminating PSVs” that may arise from other co-amplified KIV domains.

1. Primer binding site considerations: The primers were taken from a former publication of our institute (Noureen et al. PloS One 10, 2015; doi:10.1371/journal.pone.0121582.g001). In hg38 the forward primer has a perfect match both upstream of KIV-3 and upstream of KIV-2B (i.e. the third KIV-2 repeat in transcription direction), but the reverse primer has a perfect match only downstream of KIV-3 exon 1.

The alignment at the end of this document (Supplementary Data for Reviewer 2) shows the potential binding sites of the reverse primer in hg38. The closely related binding sites were retrieved using UCSC Genome browser BLAT hg38 with 50 bp context sequence on either side for better alignment. Additionally, the reverse primer was searched also in NCBI BLASTn (with word size decreased to 7), as BLASTn is more sensitive than BLAT. This found additional partial hits across chr6, but only one partial hit in a relevant location, which is the *LPA* pseudogene *LPAL2*. The BLAT hits and the *LPAL2* hit found by BLASTn were subjected to a multiple sequence alignment (MSA) with CLUSTALO.The alignment is as follows and indicates that the terminal 3-4 bases of the primer indeed differentiate the related targets and the primer is thus specific to KIV-3.

Twenty of the 23 bases of the reverse primer are found also within the KIV-2 region, but the three missing bases at the *non*-KIV-3 binding sites are precisely the last bases at the 3’ end of the primer. This primer thus acts like an ARMS-PCR primer with three unmatched primer at the 3’ end. The used Qiagen Hotstar Polymerase has no 3’-5’ exonuclease activity (as required for ARMS-PCR setups) and is thus not able to extend the primers with unmatched bases at the 3’ end.

Finally, to ensure that nothing is missed by BLAT and BLAST (given the short sequences of the two primers), we used also SNAPGENE v7 (GSL Biotech LLC, Boston, MA, USA) to extract manually every homologous position across all KIV domains for both primers by searching for

the primer sequences and allowing for gaps or mismatches at every 4 bases or more (maximum degeneration level allowed by SNAPGENE). This allows a very high degeneration and is a very sensitive additional search, finding even only slightly similar homologous sequences. These alignments are reported at the end of this document. The primers have multiple mismatches to all other potential targets, with multiple KIV even missing a binding site at all.

2. Considerations regarding the Sanger traces: The specificity of the PCR amplicon can be inspected also in the Sanger traces, as suggested by the reviewer.

Our Sanger traces cover at least 520 bases per sample sequenced completely in both directions and with high quality (average +/- SD of percentage bases >Q30 = 94.15%+/-0.03;

>Q20 = 98.02%+/-0.01; quality values as reported by Sanger analysis software Sequencher 5.4). This covers the complete first exon of the kringle, 191 bases upstream of the exon and 170 bases downstream of the exon (chr6:160611371-160611891 in hg38; all designations for strand orientation like upstream or downstream are given respective to the transcript orientation, which is on the minus strand). We are thus confident that we would have been able to see PSVs originating from other KIV domains, as long as they would be above the signal admixture level detectable in Sanger sequencing. Besides the specificity of the primers described above, also some aspects of the Sanger data makes us confident that we really sequenced KIV-3.

Firstly, the peaks for heterozygous SNPs in the electropherogram show roughly 50:50 peak heights, which would be unlikely, if we would amplify a mixture of multiple KIV units (especially multiple KIV-2 units, which are most similar to the KIV-3 based on the primer alignments). Exemplary screenshots for 4 samples are provided in Additional File 3 as Supplementary Note

- Figures 1 and 2 (both sequencing primer orientations)

Secondly, BLAT (hg38) finds the consensus Sanger sequence only in KIV-3. A match to the KIV-2B unit in hg38 had 97.4% identity and additional partial matches (bases 13 to 520) where found in the other KIV-2 units. All other KIV units (KIV-1, KIV-4 ot KIV-10) had much lower base identity levels in BLAT. We thus extracted about 520 bases for each partial hit in KIV-2B and KIV-2 and did a MSA.

The Sanger-sequenced region contains 40 PSVs between KIV-3 and KIV-2 (red bases in alignment 3 in Additional File 3). 15 PSV are seen also between KIV-3 and KIV-2B (yellow highlights in alignment 3 in Additional File 3), which is the most similar among other KIV unit.

Manual inspection of all our electropherograms did not find any hint for signals originating from other KIV units. Seven KIV-3/2B differences are clustered in the intron immediately downstream of the exon (chr6:160,611,490-160,611,540 in hg38). These bases are underlined in the alignment 3 (Additional File 3) and some exemplary Sanger traces are shown in the exemplary screenshots (Additional File 3). No signal admixture is seen.

Addition of KIV-4 to this evaluation as the next most similar KIV domain (albeit the reverse primer would bind in KIV-4 only with the last 4 mismatched bases at the 3’ end and would thus actually not be extended; see primer alignments discussed above) would result in about 120 PSVs, including several indels (alignment 4 in additional file 3). These were not observed. Especially a 22 bp large intronic insertion that differentiates the KIV-4 sequence from the homologous KIV-2 and KIV-3 was clearly not seen.

Overall, we found no signs of signal admixture in the Sanger traces and the PCR primers have multiple differences to other homologous KIV units. These considerations make us confident that the amplicon amplifies indeed only the KIV-3 unit and thus the “signature SNP” is indeed located there. We acknowledge that a very minor amount of admixture from other KIV domains (roughly <10%) would not be seen in Sanger sequencing, but such an admixture would also not affect our conclusions, since the “signature-SNP” shows a clearly germline SNP-like pattern in the Sanger sequencing data (roughly 50:50 signal ratio for the two bases; see exemplary screenshot in Additional File 3).

We totally agree that convincing the readers that we have indeed sequenced the exon of interest is crucial. We therefore created a new Supplemental note (Additional File 3) that summarizes the considerations above and shows the respective multiple sequence alignments and Sanger sequencing screenshots.

Major

*1. I was surprised when authors stated that they're using the read coverage metric to determine 2B status, but what really surprised me was that they did not specify coverage of what, nor did they specify how coverage is a meaningful metric. They don't even seem to check if the reads have a T or C allele from the following sentence: "Using the available WES data from the non- KIV-2B samples (n=12), we calculated an empiric threshold to separate between the two different sample subtypes using the mean read coverage plus 2 SD (see Additional File 1-Supp. Table 6). This value has been used to dynamically assign the optimal ROI."*

We thank the reviewer for raising this point and we can clearly see that our description in the previous manuscript was too unspecific in this point. We updated the manuscript accordingly. Briefly, the workflow is as follows: in a first step, we extracted all FASTQ reads of the *LPA* region from our available gold-standard samples in BAM format. In a second step, we analyzed each FASTQ read and verified if the defined pattern that encompasses the T-allele (CCACTGTCAC**T**GGAA) is included. Finally, we counted the number of such pattern hits. All mentioned steps have been automatically using a pattern search method to do so (https://github.com/genepi/vntr-calling-nf/blob/main/bin/PatternSearch.java).

Using this method, two clearly separated clusters have been detected (mean of pattern count in KIV-2B samples: 370, mean of pattern count in non KIV-2B samples: 14). The discrimination value (the empiric threshold) has then been calculated by using the mean value (plus 2 standard deviation) of the number of pattern occurrences in the available non KIV-2B samples (n=12) from our gold standard dataset. This value has then been applied to UK Biobank. Our UKB validation shows that the discrimination value performs well also in this large dataset and gives reasonable values for KIV-2B vs non KIV-2B in Europeans within the UK Biobank dataset. In future work, we will improve this method to discriminate KIV-2B from non KIV-2B samples also in non-European samples. We are aware that also other approaches (e.g. clustering) to separate KIV-2B samples from non KIV-2B samples could be beneficial.

The updated sentence in the manuscript (page 8) is the following:

“We used the available WES data from the non KIV-2B samples (n=12) to calculate an empirical threshold of signature-sequence occurrences to distinguish between the two sample subtypes. First, we counted the number of signature sequence occurrences within each FASTQ input file. Subsequently, the mean value plus 2 standard deviations of all occurrences across all non KIV- 2B samples was calculated and used as threshold (Additional File 1-Supp. Table 6) to dynamically assign each sample to either ROI-8 (signature coverage above the threshold) and ROI-9 (signature coverage below the threshold).”

*2. Authors state that one sample was misclassified because of a "rare" T/T homozygous genotype in the following sentence: "The reason for the single misclassification was a rare T/T*

*homozygous genotype at the signature position in a non-KIV-2B individual. As a result, this sample was processed with ROI-8 despite being non KIV-2B." Please clarify why being homozygous for T explains the misclassification. This entire section was difficult to follow. Also, why is it rare? If all 2B individuals carry "at least" one T allele, that sounds like being homozygous wouldn't be especially rare. Please report the allele frequency in both data sets.*

We apologize for the confusion generated by this paragraph. We have now updated the entire section to improve clarity (changes highlighted in yellow in the track change manuscript) Regarding the sentence cited by the reviewer, we understand that we had used the term “rare” in a misleading way. The T/T genotype is indeed not rare. What we wanted to describe as “rare” is the combination of a T/T genotype at the signature position in KIV-3 combined with the absence of KIV-2B repeats. In our Sanger sequenced dataset, this combination was present only in the one single sample that was misclassified when using the signature sequence approach.

We have now extended the sentence in the manuscript in Results Section (page 8):

“The single misclassification was one non KIV-2B individual that presented a ~~rare~~ T/T genotype at the signature position. This was the only sample in our Sanger-sequenced dataset (n=66) that presented a T/T genotype at KIV-3 exon 1 position 86 despite absence of KIV-2B repeats.”

*3. After reading the entire paper, I'm clueless how this is being done and how it is being automated: "For each VNTR, LPA included, we first tested ROIs spanning through the start and end coordinates generated by automatized tools (8) (see Methods)." Referencing another paper is not sufficient for this, and unless I misunderstood, this automation is supposed to be a key benefit from this paper. I think authors are referring to the methods section titled "Paralogous sequence variant identification", but it isn't clear. I also couldn't find anywhere in methods that specifies how ROI selection is automated.*

We thank the reviewer for this comment. In our manuscript, we had used the word “automatic” in different contexts, often too unspecific. The sentence mentioned above meant that we are building on already published approaches, respectively tools, which are able to detect VNTRs in an automatic way like (a) the Tandem Repeat Finder or specific algorithm to identify large,

multi-kilobase repeats such as the KIV-2 VNTR in *LPA* (both used by Mukamel et al (Science, 373, 2021) or (b) the DarkGenomeFinder by Ebbert et. al. This means that we are using the coordinates from Mukamel et. al or Ebbert et. al to analyze already detected VNTRs (In our case, we mainly used the Mukamel et. al coordinates but also used the KIV-2 *LPA* coordinates from Ebbert et al.). Our pipeline is then able (a) to resolve variants within VNTRs, (b) to use the correct reference for *LPA* KIV-2 calling (“signature-based approach”, previously denoted by “automatically defined ROI” and now changed throughout the manuscript to “dynamically defined ROI”) in a standardized way. Also, our software-based approach to detect PSVs has been now included in the repository.

We have now updated the manuscript accordingly to better describe what is automated and hope that these changes help to solve this confusion.

1) We now clarify that tools have been used that automated the process of VNTR coordinate detection. See Page 9:

“The ROIs experiments for *LPA* KIV-2 VNTR showed that, when the information about the repeats subtypes is not considered, not available or not relevant, bona-fide VNTR start and end coordinates (ROI-8) led to the highest mean F1 score. In 2021, Mukamel et al. (8) identified 118 protein-coding VNTRs in medically relevant genes of the human genome and provided bona-fide VNTR start and end coordinates identified with various VNTR finder tools (8). Thus, these coordinates can be used as ROI for read extraction to call variants in other VNTRs. Therefore, we used our pipeline (Figure 3, i.e. without the signature-based step) to analyze and annotate the protein-coding VNTRs in five other medically relevant genes (*NEB, SPDYE3, FLG, UBC, DMBT1*) in 2,504 unrelated samples of the 1kGP(15). ~~For each VNTR, LPA included, we first tested ROIs spanning~~ ~~through the start and end coordinates generated by automatized tools (see Methods).~~ A single VNTR repeat unit as defined in(8) was used as a reference. Since the *LPA* gene was also included in the list from Mukamel and colleagues, we first analysed the *LPA* KIV-2 VNTR in 1000G data using as ROI the start and end coordinates generated by Mukamel et al. (8).”

2) Also on Page 9 (See also Major point 4 in this document):

“To exclude them from downstream analyses, we thus compiled a list of existing PSVs between the repeats for each investigated VNTR (see https://github.com/genepi/vntr- calling-nf) and estimated the expected conserved bases for each VNTR (see Table 3).”

3) Page 10:

“Using the ~~automatically defined~~ ROI defined in (8) as a single region of extraction, independent of the KIV-2B presence or absence, we successfully detected biologically functional variants like the Lp(a)-lowering splice-site mutation 4925G>A (12) and 4733G>A (14) (carrier frequency of 9.7% and 12%, respectively, consistently with previous findings (2)).”

4) We changed the wording from “automatically defined ROIs” to “dynamically defined ROIs” at page 4:

“To this end, we devised a strategy to improve the accuracy of LPA KIV-2 variant calling by leveraging specific PSVs to inform the optimal variant calling strategy in a dynamic sample-specific ~~automatic~~ manner.”

5) We changed the wording at several locations within the discussion, see page 14:

“This suggests that using stringent coordinates for read extraction, limited to the actual VNTR start and end positions, is needed to improve variant calling accuracy, which puts additional challenges to ~~automated~~ VNTR-finding algorithms.”

“ROI-8 extracted reads from the bona-fide start and end VNTR coordinates and was the best performing strategy overall, with sensitivity and specificity comparable to the ~~automatically defined~~ previously published ROI-3 […]”

“[…] which thus appears as fixed and single-copy to previously used VNTR-finding algorithms (3, 8, 32).”

*4. The following sentences says to see the methods. I could not find this in the methods. "To exclude them from downstream analyses, we thus compiled a list of existing PSVs between the repeats for each investigated VNTR (see Methods) and estimated the expected conserved bases for each VNTR (see Table 3)."*

We thank you for raising this point. We have now updated the manuscript on page 9. To support users, we also added the necessary workflow and our developed methods to our GitHub repository adding a new section to reproduce our results including PSV detection using our software-based method for inter-repeat changes (https://github.com/genepi/vntr-calling- nf/tree/main/paper_analysis).

We updated the following sentence to Results at page 9:

“To exclude them from downstream analyses, we thus compiled a list of existing PSVs between the repeats for each investigated VNTR (see https://github.com/genepi/vntr-calling-nf) and estimated the expected conserved bases for each VNTR (see Table 3).”

*5. As mentioned in passing, the methods need way more detail and should be easily linked to sections of the results. e.g., include text about how ROIs are selected and how it's automated.*

Thank you for this comment. We added details to the manuscript at several locations (see our replies earlier). We tried to clarify what is meant by “automated approach” (Nextflow DSL2 workflow, PSV detection, dynamic approach to select KIV-2B reference vs non KIV-2B reference) and used the word “dynamically” to clearly separate the ROI selection from the VNTR coordinate identification. We indeed present an automated way to resolve *variants* in VNTRs but building on previous work from your group and the Mukamel group to select coordinates from already detected VNTRs.

*As discussed, I think this is great work overall. Please improve the paper itself.*

Dear Dr. Ebbert, we really want to thank you for your time and your in-depth analysis of our manuscript. The manuscript benefited tremendously from your feedback and we highly appreciate your comments!

We are also really excited to have UK Biobank results included, which shows that (a) the approach works on large datasets, (b) is able to replicate previous findings and (c) also helps to detect new important associations of variants within the *LPA* KIV-2 repeat. Reading your work on dark or camouflaged genomes was critical for our paper and helped us to connect the dots. We tried to carefully address each of your points and we are looking forward to your reply!

# Reviewer 3

*“Resolving medically relevant VNTRs from short-read sequencing data using the cardiovascular risk gene LPA as a model” by Maio et al. describes a nextflow pipeline for targeted SNV calling in VNTRs. Calling variants in VNTRs is challenging due to high sequence homology between repeats. By extracting reads aligned to a targeted repeat region and realigning them to a reference motif the pipeline identifies low frequency variants present in one or more repeat copies. The pipeline is robust and straightforward to use. I was able to run the pipeline on my linux workstation with both the test dataset provided in the github repository as well as on a short-reads whole genome sequencing dataset I had on my system without error. By applying the pipeline, developed and validated for the KIV-2 repeat in the medically relevant LPA gene, to five other medically relevant genes with VNTRs the authors were able to generate a population dataset of variants in these VNTRs along with variants in LPA KIV-2 for for nearly 200,00 samples in the UK biobank. These population datasets will serve as a valuable resource*

*for the community to analyze and characterize variants in these medically relevant genes*

We thank the reviewer for this very positive initial statement, the very constructive overall feedback and the time invested running the pipeline on an independent dataset. We also want to take the opportunity to mention that we further improved the Nextflow workflow during the review phase as follows: The variant calling step is now executed on each sample independently, providing a better utilization of large cluster architectures and also lowering the memory requirements for individual calling processes (see also Additional File 7). As suggested by another reviewer, we also added a more detailed analysis in the UK Biobank data which identified 9 SNPs located within the *LPA* KIV-2 VNTR region that are significantly associated with stepwise decrease in Lp(a) concentrations and an according stepwise reduction in cardiovascular events (see Results on Page 11, track change manuscript). We think that these results emphasize the potential and medical relevance of variant detection within VNTRs using a standardized and well-tested pipeline.

All major changes to the manuscript have been highlighted in yellow in the track change manuscript and are copied into this document with the corresponding page number. We also

provide a clean version of the manuscript.

Main Issues - Introduction

*Providing the KIV-s domain size in base pairs as well as information about LPA gene size and*

*how this varies for the population would help readers conceptualize how challenging calling variants in VNTRs is.*

We agree that more information about the KIV-2 VNTR in the *LPA* gene will help the readers. We expanded the introduction (page 1) to read as follows:

*“LPA* contains 10 highly homologous kringle IV (KIV) domains (see Figure 1A), whereas the KIV-2 domain represents a VNTR (see Figure 1B) with up to 40 repeats per allele (11). It can encompass up to 70% of the LPA coding sequence (12). Each KIV-2 unit is ≈5.6 kb large, creating an ≈200 kb large VNTR array, and a variant present in only one repeat may show as little as 1/80=1.25% fractional representation. Each KIV domain is encoded by two short exons (mostly 160 bp and 182 bp), spaced by a mostly ≈4 kb intron. A ≈1.2 kb intron links the single KIV domains. The exons of the KIV-2 present up to 98% base identity to the respective exons of the flanking KIV units, especially KIV-1 and KIV 3 (2). The second exon of KIV-1 is identical to the second KIV-2 exon and ≈70% homology is observed even within the first 200 intronic bases flanking the exons. This complicates the definition of the VNTR boundaries, read alignment and mutation detection. Moreover, the KIV-2 domain exists in at least three nearly identical subtypes (denoted by KIV-2A, B and C), which differ in the haplotype of three synonymous variants, as well as of >100 intronic variants (11) (see Figure 1C). The first exon of KIV-2B is identical to the first exon of KIV-3, while the respective second exons show 96%

base identity.”

*“This dataset also served us as a gold standard to evaluate variant calling from whole-genome sequencing (WGS) or whole-exome sequencing (WES) data.” Please revise to clarify that this*

*dataset is for evaluating variant calls in the KIV-2 region of the LPA VNTR.*

We apologize for the imprecise wording. We have rewritten parts of the introduction to address the comments of all three reviewers. The sentence mentioned above has been slightly modified

and moved to a different location in the introduction to improve clarity (page 4). It now reads:

“We used our highly specific amplicon-based dataset (2) as a gold-standard to evaluate and improve variant calling in the LPA KIV-2 VNTR from WGS or WES data.”

*Include a brief description of how the truth variant calls for Datasets A and B were determined to provide readers with confidence in the accuracy of the truth set without having to go to the*

*citation unless they are interested in detailed description of the truth set generation.*

This is a well-taken point. We have now expanded the description of the generation of the truth set.

1) In the background section, we have rephrased the text at page 3 to improve clarity

“To circumvent these difficulties, we have previously sequenced the KIV-2 repeats using targeted KIV-2 amplicons that amplify only the KIV-2 units in a highly specific manner

(2) (see Figure 2A). After deep sequencing, all reads from these amplicons are aligned to one KIV-2 repeat and KIV-2 mutations are called as low-level mutations, excluding so confounding reads from homologous regions.”

2) In the Results section, page 5, we have now included a sentence that points to the Methods section for a brief description of the generation of the truth set:

“We generated WES data for two datasets (dataset A, dataset B), with known KIV-2 variant patterns from our previously established KIV-2 variation dataset (2) (referred to as gold-standard. See Methods for a brief description of the gold-standard generation).”

3) In the Methods section, page 17, we have rephrased and expanded the description of the ultra-deep sequencing approach:

“In brief, the experiment consisted of 3 main steps and was conducted in 123 Central- European individuals. First, a single primer pair was used to selectively amplify all KIV- 2 units as a mixture, leveraging their high sequence homology (“batch amplification approach”) (25). Second, the resulting amplicon mixture subjected to NGS and the KIV- 2 reads were mapped to one KIV-2 repeat as a reference. Since KIV-2 variants can be present in one or few repeats, only a subset of reads will present the respective mutation. Thus, in the third step, a variant calling tool suitable for low-level mutations was used to detect mutations down to 1% mutation level (2). Among the samples in

dataset A and B, 16 samples did not present KIV-2B units (non KIV-2B samples).”

*The manuscript would benefit from a description of other VNTR variant callers (e.g. https://genome.cshlp.org/content/33/4/511.full	and https://*[*www.ncbi.nlm.nih.gov/pmc/articles/PMC9379575/)*](http://www.ncbi.nlm.nih.gov/pmc/articles/PMC9379575/)) *and how the method presented here*

*differs from existing methods.*

We thank the reviewer for raising this point. We added the following paragraph including a comparison to related work to the Discussion, page 15:

“Several methods exist to estimate the repeat length as well as mutations within VNTRs from short-read sequencing data. The computational approach presented in (34) is based on hidden Markov models and is specifically focused on repeat unit count estimation. This approach has been further extended in (35) to identify insertions and deletions as well as small frameshifts within VNTRs. Work presented in (36) utilized a repeat-pangenome graph (RPGG) based on haplotype-resolved assemblies, which allowed the detection of variation in both VNTR length and repeat composition. The work in (37) presents a method to discover genetic variations in duplicated genome regions by applying a masking approach to the reference genome. Unlike these approaches, our approach allows the direct detection of intra-repeat mutations, also known as batch sequencing in the field of *LPA* genetics (38). This strategy has been originally utilized to amplify and sequence repeats as amplicon mixtures and detecting variants alike somatic mutations by aligning all data to one single repeat. Ebbert et. al conceived independently a similar approach and described for the first time how such an approach can recover variants within unmappable regions (denoted as camouflaged regions) in a genome- wide manner using WGS and WES data (3). Additionally, an algorithm has been outlined to rescue a rare ten-nucleotide frameshift deletion in the CR1 gene (3). Building on this work and VNTR coordinates determined in (8), we are able to present a computational pipeline targeting the recovery of SNVs from coding VNTRs. We especially focused on the complex *LPA* KIV-2 region. Our approach implements a dynamic adaptation of the read extraction steps leveraging a SNP in the flanking region of the VNTR, which predicts the presence of KIV-2B units. We show that this significantly improves variant calling even more, unlocking access to this medically relevant region and shows the potential for other VNTRs. Representing VNTRs in the widely used variant calling format (VCF) poses an ongoing challenge. Future work requires either the development of a new file format or the enhancement of the VCF file format capable of accurately representing VNTRs, in addition to the creation of novel benchmarking tools for

VNTRs.”

*Please clarify that this method is SNP calling in targeted VNTRs using WGS or WES data. Specifically noting that the pipeline only calls variants in one VNTR at a time and does not call*

*indels.*

We thank the reviewer for this comment. We have changed the wording in several sentences of the manuscript to better clarify the task of our pipeline, and clearly specified in the discussion that the current version of the pipeline does not call indels. Prominent changes are especially at the following positions:

1. Title:

“Resolving variants in medically relevant VNTRs from short-read sequencing data using the cardiovascular risk gene *LPA* as a model.”

2. Abstract:

“Using the highly complex *LPA* gene as a model, we developed a computational approach to resolve intra-repeat variation in VNTRs from largely available short-read sequencing data.”

3. Results, page 4:

*“*We present a computational approach to resolve intra-repeat variation in VNTRs from short-read sequencing data, and […]”.

4. Results, page 9:

“The final call set for each analyzed VNTR using the 1kG samples is provided at the pipeline repository (see https://github.com/genepi/vntr-calling-nf) and represents the fully resolved call set of variants within the six analyzed VNTRs.”

5. Limitation, page 16:

“Our approach also has limitations. […] Second, while we are currently focusing on the identification of SNVs, the underlying architecture allows us to expand the approach also to the detection of insertions and deletions in the next pipeline versions. Given the modularity of the nextflow pipeline, this can be implemented also by any other users with a suitable variant callers at hand.”

Main Issues - Methods

*Nextflow pipeline targeted snv calling in VNTRs*

- *Please include a url for the pipeline github repo*
- *Include bed and fasta files used for additional gene analysis in github repo with*

*documentation / guidance on creating files for additional VNTRs.*

- *Please provide additional details about the output tsv and vcf files, specifically how they are customizable and can be annotated.*
- *Include metric definitions in documentation, noting when NAs are reported*

We thank the reviewer for these points. As suggested above, we have now included the URL for the GitHub repository in the Methods section, page 19:

“We implemented our variant calling approach for short-read VNTRs data in a parallelized workflow (Figure 3) built in Nextflow using DSL2. The pipeline is publicly available at https://github.com/genepi/vntr-calling-nf.”

We have now added additional information to the repository. The “paper_analysis” section (https://github.com/genepi/vntr-calling-nf/tree/main/paper_analysis) includes all required information on how to download the data and run the pipeline, it includes results and also describes the annotation process. This part is also linked in the overview README file.

In the manuscript, we updated the following sentence in Results on page 9:

“The final call set for each analyzed VNTR using the 1kG samples is provided at the pipeline repository (see https://github.com/genepi/vntr-calling-nf) and represents the fully resolved call set of variants within the six analyzed VNTRs.”

The supplementary methods in Additional File 2 now also include a description of the annotation process:

“We use customizable tab-separated files (internally named MapLocus file) to annotate genetic features within the VNTRs. The files use coordinates referring to the VNTR reference sequence used for variant calling to map freely definable annotations to the sequence. If a feature is protein-coding, also the translation frame can be defined, allowing automatic determination of amino acid changes with our annotation script (see https://github.com/genepi/vntr-calling- nf/tree/main/paper_analysis).

The annotations for all VNTRs analyzed in the present manuscript expect *LPA,* were defined by mapping the VNTR repeat unit consensus sequences defined by Mukamel et al., Science,

2021 (Supplementary Table 17) to hg38 using BLAST. The annotation of the *LPA* KIV-2 was

available from previous projects (Coassin, Schönherr et al., J Lipid Res, 2019 and Coassin et al., Eur Heart J, 2017) For single exon VNTRs, the genome coordinates of the best hit (respectively the first hit in translation direction, if multiple or all repeats had the same BLAST quality values, like *FLG*), were looked up the UCSC Genome browser hg38 and the translation frame was determined visually (UCSC Gencode v43 data track).

For multi-exon repeats, the UCSC hit was used to determine, which exon numbers were encompassed by the selected VNTR unit and the translation phase of all exons in the VNTR unit was taken from the “Exons” overview in the respective Ensembl v109 transcript data sheet. This was visually confirmed in the UCSC Genome browser. If the human genome reference contained multiple VNTR repeats, we cross-checked that also the exons in the subsequent repeats would have the same translation phase. Additionally, the UCSC Genome browser annotation was used to add to also intron regions the maplocus file.

This manually curated procedure ensures a precise representation of the VNTR unit structure and can be applied in principle to any other VNTR as well, provided that the reference sequence and gene structure annotations are available. However, if the human genome reference sequence missed annotations of the genetic elements within the VNTR, this would affect only the annotation of mutation locations and amino acid effects, not variant calling itself.”

Regarding NA, we have described in the legend in the Additional File 1 as not possible to calculate. However, we realize now how NA can be confusing. We apologize for the imprecise wording and we have changed to “-” at every occurrence (Table 2 in the manuscript and in Supplementary Tables 4 and 18 in Additional File 1. In the caption, we define “-” as “no calculation possible”.

*Figure 3. Is the subsequent variant annotation part of this workflow, if not please clarify.*

We thank the reviewer for raising this point, which certainly improves the usability of our workflow. The variant annotation is currently not part of the workflow. However, we have now added a section to the Github repository and made our Java repository and instruction for variant annotation publicly available (see *https://github.com/genepi/vntr-calling- nf/tree/main/paper_analysis*). We now explain this in the manuscript at the following positions and added a description of the annotation process to the Additional File 2 (described in our previous reply):

1) Figure 3 caption:

*“…*(step 5), resulting in a VCF output file that can subsequently be annotated (see Github repository in the Availability of data and materials section).”

2) Figure 6 caption:

*“…*(step 5), resulting in a VCF output file that can subsequently be annotated (see Github repository in the Availability of data and materials section).”

*We applied the pipeline to 2,504 samples from 1kGP for 5 other protein-coding VNTRs showing VNTR units that are longer than the read length and include less than 50% PSV positions.”*

*Unclear statement maybe use “with” instead of “showing”*

We appreciate the suggestion and we have changed the sentence as proposed at page 20.

*Fig 4 - Datasets A and B not balanced for KIV-2B and non-KIV2B samples. Please note how*

*were the samples assigned to dataset A or dataset B? Does the lack of balance bias the downstream analysis?*

We apologize for not being clearer on this point. Datasets A and B include only European samples with known KIV-2 variants patterns from our previous work (Coassin, Schönherr et al, J Lipid Res 57, 2019). Dataset A matched the reported frequency for KIV-2B subtypes in Europeans, which is about 80%. Dataset B is enriched with *non KIV-2B samples* to facilitate the investigation of strategies to separate between samples with and without KIV-2B subtypes. Following this comment, as well as also a comment of reviewer 2, we added the following sentence to the manuscript in the Results section page 5:

“In particular, the enrichment for non KIV-2B samples in dataset B allowed the investigation of strategies to account for the presence/absence of KIV-2B subtypes in the variant calling workflow for the KIV-2 VNTR.”

We would also like to address the reviewer's question about possible bias. Our Sanger sequencing experiment (n=66) showed that:

- all KIV-2B carriers (50) carried at least one T allele at the signature position 86 in KIV- 3 exon 1.
- Among the 16 non KIV-2B individuals, 11 were homozygous for C/C at the signature

position, four were heterozygous (T/C) and one individual was homozygous T/T despite

the absence of KIV-2 B repeats. This sample was the only sample misclassified when screening for the signature sequence (see Results, page 8).

As described above, the enrichment for non KIV-2B samples in Dataset B had technical reasons and allowed us to better investigate the effect of our read separation strategy. It thus helped us to improve our variant calling strategy.

Our results suggest that misclassifications would likely occur only in a small fraction of the non KIV-2B samples, which in turn represent only a minority of the total European population (about 20%). As explained in the figure in Additional File 2, this would translate to only ≈6% of the individuals in European populations being misclassified. Enriching our evaluation dataset (dataset B) for these most challenging samples might have indeed decreased the overall the variant calling performance reported in our manuscript compared to the variant calling performance in a fully randomly selected population-based dataset, like dataset A. We are thus confident that any bias potentially introduced by this selection strategy would thus have affected our results only in a more conservative direction and would have affected only the performance presented in the paper, not the validity of the variant calls itself. In the manuscript, this point was already briefly addressed at page 9:

*“*For dataset B, when using the dynamic signature-based approach the F1 increased from 63.2% to 90.8% (+43.6%; compared to ROI-8 only) and showed less variability among samples (see Figure 7). After excluding the misclassified sample, the F1 reached 94.7% (see Additional file 1-Supp. Table 9).”

*Figure 6 - signature-based strategy: Clarify in figure that reads from the full LPA region were*

*used when searching for the signature sequence.*

This is a well-taken point, which can help readers understand the workflow. We now rephrased the caption of figure 6 as follows:

“**Panel B)** Workflow of the signature-based approach for KIV-2 variant calling in WES data: starting from the input aligned BAM file (step 1), the entire *LPA* region is screened in FASTQ format for the KIV-2B signature sequence and (step 2) each sample is redirected to the optimal ROI strategy for (step 3) KIV-2 read extraction. […]”

Main Issues - Results

*Unclear total number of variants for individual samples TP, FP, FN as the total number of variants seems small using F1, precision, sensitivity, and specificity performance metrics alone might be misleading at times. Good to include the raw variant numbers. Quantifying statements like “but with less false positive calls and therefore higher mean F1-score (dataset A experiment-1 and experiment-2: 81.9%, and 84.6%).” with changes to the number of false positives. This would provide readers with a clearer understanding of the improvements. The impact of different methods on the F1-scores are rather large at times and if the total number*

*of variants is small only providing the the changes can be misleading.*

We thank the reviewer for raising this point. We have now added the TP numbers in the supplementary tables 4, 8 and 9 in the additional file 1. The expected number of variants per sample is provided in Additional file 1-Supp. Table 1. We updated the sentence as follows:

“ROI-8 extracted reads from the bona-fide start and end VNTR coordinates and was the best performing strategy overall, with sensitivity and specificity comparable to the automatically defined previously published ROI-3 (see Additional file 1-Supp. Table 4) but with less false positive calls (ROI-3: from 8 to 20 in experiment 1 and 2; ROI-8 from 0 to 12 in experiment 1 and from 0 to 11 in experiment 2; see Additional file 1-Supp. Table 4) and therefore higher

mean F1-score (dataset A experiment-1 and experiment-2: 81.9%, and 84.6%).”

*Computational Workflow for variant calling in VNTRs*

*Please revise “We developed a complete computational workflow (see Figure 3) to detect variants in VNTRs at scale.” to clarify that this method is for calling variants in targeted/ specific VNTRs.*

We appreciate the comment and apologize if our wording was misleading. We have rephrased the manuscript in several points, following the reviewers suggestions. The sentence cited above was removed during these adaptions, but we now clarify the reviewer´s point at page 5:

“For simplified and scalable application to user-defined VNTRs, the pipeline has been implemented using the Nextflow workflow manager (24).”

*“The reason for the single misclassification was a rare T/T homozygous genotype*

*at the signature position in a non-KIV-2B individual.” How rare is this mutation, is there population data to indicate how often this will cause a KIV-2 classification error?*

We have now updated the paragraph to improve clarity. We understand that we had used the term “rare” in a misleading way. The T/T genotype itself is indeed not rare. What we wanted to describe as “rare” is the combination of a T/T genotype at the signature position in KIV-3 combined with the absence of KIV-2B repeats. In our Sanger sequenced dataset, this combination was present only in the one single sample.

Our results suggest that misclassifications would likely occur only in a small fraction of the non KIV-2B samples, which in turn represent only a minority of the total European population (about 20%). This would translate to only ≈6% of the individuals in European populations being misclassified (see also reply to the point 4 in Main Issues-Methods (above) and Additional File 2).

We have now also edited the sentence mentioned in this present comment in the Results Section on page 8 to read as follows:

“The single misclassification was one non KIV-2B individual that presented a ~~rare~~ T/T genotype at the signature position. This was the only sample in our Sanger-sequenced dataset (n=66) that presented a T/T genotype at KIV-3 exon 1 position 86 despite absence of KIV-2B repeats.”

In our Sanger-sequenced dataset, the TT genotype (known also as rs113727842; mentioned in the manuscript (page 14) was present in 24 individuals (36%), which matches genotype frequency reported in Europeans of the 1000G data in Ensembl (35%) (However, in our experience, the publically available allele frequency of SNPs located in the regions immediately adjacent to the KIV-2 VNTR are often unreliable and have to be considered with some care). Unfortunately, we are not aware of any independent dataset about the frequency of the combination of the AA genotype and absence of KIV-2B units is available. We have pointed out the need for this future research and the ancestry-limitedness of these findings in the Limitation section of the manuscript. Nevertheless, we believe that our demonstration of the use of a SNP to improve read mapping in VNTRs with subtypes can be still a useful and important message

for others in the field. We hope that others can build on and refine this method. Limitation section, page 16:

“Importantly, this limitation applies only to the use of the KIV-2B signature sequence, which could be designed only for individuals of European ancestry. The other parts of the optimal ROI selection, which already considerably boosted calling accuracy, are ancestry independent and allowed analyzing five other medically relevant VNTRs, currently limited to a similar repeat structure as *LPA* (i.e., VNTR unit larger than the read length).”

Additionally, we also describe in the manuscript what would happen if the wrong ROI is used. Indeed, this would affect only KIV-2B specific positions (page 7):

“Compared to ROI-8, ROI-9 does not extract reads mapped to the first exon of the KIV-2B repeat. Since no true KIV-2B reads exist in non KIV-2B individuals, the reads that are aligned in these individuals to the KIV-2B repeat of the reference genome must originate from highly homologous domains, most likely from KIV-3, which is nearly identical to the KIV-2B. Thus, in non KIV-2B individuals, ROI-9 skips the KIV-2B domain in the reference during read extraction and so reduces false positive calls. ROI-9 improved the F1 in non KIV-2B individuals to 95.5% and to 100% in the two sequencing experiments in dataset A (see Discussion). We thus investigated strategies to minimize the false positive rate by accounting for the presence/absence of KIV-2B subtypes in a dynamic way within the Nextflow analysis pipeline.”

*“The final call set for each analyzed VNTR using the 1kG samples is provided at https://github.com/genepi/exome-vntr-nf and represents to the best of our knowledge the first fully resolved and computational reproducible version of these VNTRs.” I was unable to find*

*these callsets in the repo*

We apologize for the lack of clarity. As mentioned also above (main issues-methods, point 1), we have now added a new part to the repository (https://github.com/genepi/vntr-calling- nf/tree/main/paper_analysis) that includes also the final VNTR variant results. This part is also linked in the overview README file. The sentence at page 9 has now been updated:

“The final call set for each analyzed VNTR using the 1kG samples is provided at the pipeline

repository (see https://github.com/genepi/vntr-calling-nf) and represents the fully resolved call set of variants within the six analyzed VNTRs.”

*Genomic samples and datasets - please include a description of the 66 sample*

*dataset.*

We originally wanted to keep the description of the epidemiological sample collection rather short to avoid adding too much complexity, but we fully agree that some more details about the population from which the samples were selected is important (described then in more detail in our previous publication paper Coassin, Schönherr et al, J Lipid Res, 2019). We amended the Methods section (page 18) as follows:

“For the KIV-2B signature sequence experiments (see below), the sample set above has been complemented with further 42 samples from the Austrian healthy worker study (40) SAPHIR that had undergone amplicon-based targeted KIV-2 deep sequencing during the same previous project (2, 12). Details about the SAPHIR study are given in Additional file 8.”

The text in the Additional file 8 reads:

“The Salzburg Atherosclerosis Prevention Program in subjects at High Individual Risk (SAPHIR) is an observational study involving healthy unrelated subjects: 645 females from 39 to 67 years of age and 1093 males from 39 to 66 years of age. Study participants were recruited by health screening programs in large companies in area of the city of Salzburg in Austria from 1999 to 2002. All individuals were of West-Eurasian origin. Informed consent was obtained from each participant. Details about the exclusion criteria and baseline assessment are described in detail elsewhere (Heid et al., Diabetes 55, 2006 and Coassin et al., Eur Heart J 38, 2017). Blood samples were collected after an overnight fasting period. Full Lp(a) phenotyping by ELISA (Lp(a) plasma concentration) and Western Blot (apolipoprotein(a) isoform sizes in plasma, reflecting the KIV-2 VNTR repeat number) was available for n=1,522 participants.”

The Methods section “DNA sequencing” already additionally described:

“To identify the KIV-2B signature sequence, we selected 66 samples from the KIV-2 variability

dataset (2), including the WES sequenced samples from dataset A and B. The KIV-3 repeat was Sanger-sequenced to identify sequence differences between the first exon of KIV-3 and

KIV-2B (see Additional file 1-Supp. Table 16).”

*UK biobanks samples - comment on the ancestry grouping, any subgroup*

*potential issues or biases from the groupings?*

We thank the reviewer for this comment. A limitation of the KIV-2 variant calling is that the signature-based approach was developed in individuals of European ancestry (see page 16 of the manuscript, section Limitation). In datasets A and B, the mean F1 score ranged from 90.8% to 95.65%. These results suggested that the signature-based approach can detect variants with high accuracy in individuals of White ancestry. Thus, when running the signature-based approach in UKB participants, we decided to report results grouped by ancestry in Table 4, with almost 300 missense mutations identified in White individuals (n=186,607). Although we could not test the performance of our pipeline in non-European individuals due to the lack of suitable gold-standard samples, only the use of the KIV-2B signature sequence can introduce bias between ancestries, while all other steps are ancestry independent. Further investigations are required in non-European populations.

As mentioned in a previous point, we have expanded the limitation paragraph to better address these points (page 16):

“Importantly, this limitation applies only to the use of the KIV-2B signature sequence, which could be designed only for individuals of European ancestry. The other parts of the optimal ROI selection, which already considerably boosted calling accuracy, are ancestry independent and allowed analyzing five other medically relevant VNTRs, currently limited to a similar repeat structure as *LPA* (i.e., VNTR unit larger than the read length).”

Moreover, we have now clarified this in the caption of Table 4.

**“Table 4:** Unique *LPA* KIV-2 mutations within the target regions (KIV-2 exons 1 and 2 ±100 bp) in each population. KIV-2 mutations are divided by mutation type for UK Biobank individuals (n=199,119). Since the signature-based approach was developed and benchmarked in individuals of European ancestry, summary data are here filtered for White (n=186,607), South Asian (n=3,465) and Black (n=3,200) ancestry. The PSVs defining the KIV-2B were not

considered as mutations. The location “splicing region” was defined as within the 25 bp

upstream and downstream KIV-2 exons.”

*To support claims regarding the methods ability to call variants in VNTRs from WGS and WES*

*data it would be useful to compare WGS and WES based variant calls for the same samples.*

We thank the reviewer for this comment. As described in the manuscript, we evaluated and developed our pipeline using WES data generated for dataset A (n=8) with two independent sequencing experiments and applied it subsequently to 200K samples from UK Biobank. In the updated revision, we now include also additional epidemiological analyses in UK Biobank analyses using the detected KIV-2 SNPs. We believe that these results (Figure 9), further support the ability of our pipeline to call variants from WES data.

We think that there are no fundamental technical reasons that would prevent or limit the transferability of our workflow to WGS, but we understand that a formal demonstration that the pipeline also works for WGS is important. The most important differences between WES and WGS reside in the considerably larger data size of WGS and in the target capturing process present in WES but not in WGS. The former does not present an issue for our pipeline due to the sample-wise parallelization using Nextflow. The latter would most likely affect WES-called variant levels, with WGS being expected to give even better performance (as we have seen also in our previous publication Coassin, Schönherr et al, J Lipid Res 57, 2019). Barbitoff et al, BMC Genomics 23, 2022 showed recently that accuracy of SNP discovery was comparable for WES and WGS, while indel variants are harder to call using exome data. This may further support that no major drawbacks are expected when applying our current SNP-centered pipeline to WGS data.

We did not have WGS data for our gold standard data. However, meanwhile we have compared the results from applying our pipeline to short-read WGS data to highly sensitive and highly precise Nanopore Sequencing data with “unique molecular identifiers”-based error correction (UMI-ONT-Seq). We have very recently made these results available as a preprint at https://[www.biorxiv.org/content/10.1101/2024.03.01.582741v1,](http://www.biorxiv.org/content/10.1101/2024.03.01.582741v1) currently under revision at Genome Medicine. By implementing UMI-mediated error-correction at single molecule level,

UMI-ONT-Seq -allowed us to retrieve the SNP calls and haplotypes with utmost accuracy and

even precisely quantify the KIV-2 VNTR copy number. We believe that it may in fact serve as a new gold-standard.

In the preprint mentioned above we compare *LPA* KIV-2 variants from the 1000 Genomes WGS dataset (n=48) with UMI-ONT-Seq data coming from third-generation sequencing. Figure 6B (see below) of the preprint shows the correlation between the levels of KIV-2 variants detected with our variant calling pipeline applied to WGS data UMI-ONT-Seq data (r-squared=0.984). These results strongly support the ability of our pipeline to call variants from WGS data.

We added the following sentence to the paper, page 13:

“In another recent study we have compared the *LPA* KIV-2 variant levels quantified with our pipeline to highly precise Nanopore Sequencing data with error-correction by unique molecular identifiers (UMI-ONT-Seq), finding very high correlation between the variant levels (R^2^=0.983) (31).”

*Include definitions for precision, sensitivity, and F1-score. If possible please use established standard definitions for these performance metrics, https://*[*www.nature.com/articles/s41587-*](http://www.nature.com/articles/s41587-)

*019-0054-x.*

We thank the reviewer for this suggestion. We have now integrated these information in the results section, page 6 :

“Variations in the extraction region impacted the F1-score (F1), which describes the harmonic

mean of precision (true positives/(true positives+false positives)) and sensitivity (true positives/(true positives+false negatives)) (26). Thus, F1 reflects both false negative and the false positive calls and performs well in unbalanced datasets. Higher F1 score indicates better

performance.”

*Supplementary Table 2: Precision values all 0, Sensitivity and F1-Score NA, this seems*

*inconsistent with results in the manuscript.*

We thank the reviewer for pointing this out. Supplementary Table 2 results derived from a naïve approach, where all WES reads are aligned to one KIV-2 repeat as reference. We have now updated the table using the correct values. We apologize for this copy/paste error. This also gave us the possibility to try the approach presented by Steyaert and colleagues (now Ref 37 in the manuscript), which was published in October 2023, when our paper was already submitted to Genome Biology. The authors apply a masking approach to the reference genome. When using this approach and aligning all reads to the sixth KIV-2 repeat in the reference genome, we found again >200 false positive mutations per sample. Precision ranges from ~2%

to ~10%.

*Fig 1 - To help readers understand scale please include overall size (bp) as reference coordinates (probably just GRCh38 for simplicity) and potentially size population size range*

*(average, min, max repeat copy numbers.)*

We thank the reviewer for the suggestion and have now updated figure 1 as suggested, including start and end coordinates for both panels A and B. In the caption, we have now included the apo(a) isoforms size range (measured with Western Blot) from 10,927 individuals from three Central-European populations from Mack et al., J Lipid Res, 2017. The caption now includes:

“[…] In 10,927 Central-European individuals from (44), the average, minimum and maximum of number of KIV-2 units measured at protein level in plasma using Western Blot were 20, 2 and 36 KIV-2, respectively. […]”

*Figure 3B “WGS or WES” instead of just WES to be consistent with manuscript text indicating*

*this method can be used with WES and WGS data.*

We thank the reviewer for pointing this out. We have adapted the figure and the caption as

suggested.

*Figure 5 - annotate with region sizes*

We have now included in figure 5 the size of ROI span regions reported as bp. The precise coordinates are provided in Additional File-Supp. Table 3. We have adapted the caption accordingly.

**“Figure 5:** [...] The size of the regions enclosed by each ROI is provided as bp between start and end coordinates on hg38. Precise coordinates are provided in Additional File 1-Supp. Table 3.”

*Figure 8 black lines difficult to see in the figure, initially it looked like a bar plot and not a boxplot.*

- *Assuming colors used in the two plots represent corresponding groups, it would be useful to combine panels A and B with the SNP and WES boxplots next to each other for each carrier status category making it easier for readers to directly compare values*

*between variant calling methods*

We thank the reviewer for raising this point. We have now made sure that the lines are visible and we apologize for the previous low resolution. We have also rearranged the plots to facilitate

comparison between categories, as suggested by the reviewer.

*Table 1: median (max - min) instead of mean +/- sd. Bold or italize ROI-* for emphasis*

-	*Maybe add columns with summary statistics for false positive counts for the non KIV- 2B samples*

We have now adapted the table according to reviewer suggestions, including median (min- max). Regarding the number of false positive variants in the non KIV-2B samples, we have included the minimum and maximum mean number per ROI in the table caption, and we refer to Additional File 1-Supp. Table 4 for the performance metrics at individual level.

“**Table 1:** F1-score (%) in dataset A experiment-1 and experiment-2 for each tested ROI strategy expressed as mean ± standard deviation (SD) and as median (minimum-maximum).

ROI-8 shows the highest F1 mean in both experiments. The high standard deviation is explained but the high rate of false positive variants in the non KIV-2B samples, which range from mean=0 with ROI-9 (both experiments 1 and 2) to mean=152 and mean=164 with ROI-1 (experiment 1 and 2, respectively). See Additional File 1-Supp. Table 4 for performance metrics

at individual level.”

*Table 2 legend - explain NA values*

We apologize if this terminology has created confusion. We have now changed that into “-“. This better reflects the situation where the F1-score and the other performance metrics were not possible to calculate. We have now changed also Table 2 in the paper accordingly and have added the explanation in the table caption:

**“Table 2:** Comparison between the F1-score in each sample of dataset A in experiment-1 and experiment-2 using ROI-8 and ROI-9. ROI-8 performs best in KIV-2B samples and ROI-9 performs best in non KIV-2B samples. The sign “-“ is used for “no calculation possible”.

*Table 4: providing coordinates for the different regions with reference version information. Maybe summary stats for number of mutations per individual.*

We have now added in Table 3 the VNTR the GRCh38 (hg38) coordinates used for read extraction in data from 1000 Genome. GRCh37 (hg19) coordinates are provided in Additional File 1-Supplementary table 17. We have updated the table caption accordingly:

**“Table 3** Analysis of the VNTRs in 6 genes in 2,504 WGS samples from 1kGP. The regions of interest (ROI) for VNTR read extraction were defined as in (8). […]”

Main Issues - Discussion

*Modify language to state that this method is for targeted VNTR variant calling that was*

*optimized and evaluated using the KIV-2 VNTR in the LPA gene.*

We edited the first sentence of the second paragraph in the discussion to explain this more explicitly:

“We developed a computational pipeline for targeted VNTR variant calling, optimizing and

evaluating it using the particularly complex KIV-2 VNTR in the *LPA* gene and applied it to five further medically relevant protein-coding VNTRs located in the genes *FLG*, *NEB*, *UBC*, *DMBT1*

and *SPDYE3*, supporting portability to other VNTRs with unit larger than the read length.”

*For population variant analysis - carrier mutations present in one or a few individuals, these*

*might be errors/ noise, might want to address in discussion*

We agree and have added the following text to the Limitations section:

“We acknowledge that mutations present only in one or a few individuals in the population data might also be technical errors. This may most likely apply to *LPA*, which has the highest number of repeats and thus requires calling variants down to 1.25% fractional representations.”

Main Issues - Limitations

*Please note that the variants are reported relative to the reference motif coordinates and not reference genome coordinates as is standard for vcf files. Your pipeline does this for good reason but important to highlight. I also suggest adding a statement that how to best represent variants in VNTRs and benchmarking these variants where performing whole genome variant calling is an open issue and will require changes to VCF file format specifications and*

*development of new benchmarking tools.*

We thank the reviewer for this input. We have addressed this limitation in a new paragraph added in the Limitation Section at page 16:

“Our approach also has limitations. First, while our approach works for coding VNTR that are longer than the read length, we report only the relative position of mutations within the repeat.”

Main Issues - Supplemental Material

*Supplemental file 4: Consider also providing as MSA text file, describe base highlighting, color,*

*and asterisk in legend.*

We thank the reviewer for this suggestion. We have now clarified the meaning of base

highlighting, color and asterisk in the alignment file (Additional file 5). We also provide the

alignment as MSA text file in the paper analysis section available at our github repository.

*Supplemental Table 13: please define mean variant level*

We define the variant level as the fraction of reads showing the canonical variants in KIV-2 exon 1 that define the KIV-2B units. It thus represents the percentage of KIV-2B units among the total KIV-2 units in the genome. The column "mean variant level" represents the mean level in the anaylzed population. We have now explained it also in the caption of Supplementary Table

13 in Additional File 1.

*Supplemental Table 17: should the column header for D or E be hg38 coordinates?*

We thank the reviewer for pointing this out and apologize for the imprecision. We have now

corrected the title of column D from hg19 to hg38.

Minor Edits

*Figure 1 panel B legend “The reference genome sequence shows 100% of identity between*

*the exon 2 sequence of” first of not necessary.*

We thank the reviewer for the input. We have now changed the sentence as suggested.

*Figure 1 Panel C legend “KIV-2B and KIV-3 differ from the more frequent KIV-2A by the three*

*PSV in in exon 1 at positions 14,” extra in*

We thank the reviewer for the input. We have now changed the sentence as suggested.

*Figure 6 panel b legend “redirected to the optimal ROI strategy for (step 3) KIV-2 reads*

*extraction.” reads should be singular.*

We thank the reviewer for the input. We have now changed the sentence as suggested.

*“Here, we tested three published regions that have been used o isolate KIV-2 reads from*

*WES data already aligned to the reference genome” o should be to*

We thank the reviewer for the input. We have now changed the sentence as suggested.

*Supplemental Figure 1 legend “Panel A) KIV-2 variant calling was performed extracting reads*

*from one one single region of interest (ROI)” extra one*

We thank the reviewer for the input. We have now changed the sentence as suggested.

*Discussion - “This suggests that using stringent coordinates for reads extraction” reads should*

*be singular.*

We thank the reviewer for the input. We have now changed the sentence as suggested and we

have modified similar occurrences throughout the manuscript.

47

# Supplementary Data for Reviewer 2

CLUSTAL O(1.2.4) multiple sequence alignment for reverse primer binding sites identified by BLAT

Reverse primer shown in blue font and capital letters. Mismatched bases shown in red font

Primer_rv	0

KIV-3_6:160610603-160610825	acaaaccagttct 13

KIV-2_6:160616208-160616327	gttct 5

KIV-2_6:160621754-160621873	gttct 5

KIV-2_6:160627298-160627417	gttct 5

KIV-2_6:160632844-160632963	gttct 5

KIV-2_6:160638391-160638510	gttct 5

KIV-2_6:160643938-160644057	gttct 5

LPAL2_6:160499874-160499992	aatgacttgaggagattctatacgtattcctacagctcctttcctccacaaatccattct 60

Primer_rv	AAACTCCAATCCCTC 15

KIV-3_6:160610603-160610825	tatttgtagcggacc	tagaaaataccagagtcatcccAAACTCCAATCCCTC 65

KIV-2_6:160616208-160616327	tatttctagcagacctagaaaaatttcagctaccacagtcatcccAAACTCCAATCCCTC 65

KIV-2_6:160621754-160621873	tatttctagcagacctagaaaaatttcagctaccacagtcatcccAAACTCCAATCCCTC 65

KIV-2_6:160627298-160627417	tatttctagcagacctagaaaaatttcagctaccacagtcatcccAAACTCCAATCCCTC 65

KIV-2_6:160632844-160632963	tatttctagcagacctagaaaaatttcagctaccacagtcatcccAAACTCCAATCCCTC 65

KIV-2_6:160638391-160638510	tatttctagcagacctagaaaaatttcagctaccacagtcatcccAAACTCCAATCCCTC 65

KIV-2_6:160643938-160644057	tatttctagcagacctagaaaaatttcagctaccacagtcatcccAAACTCCAATCCCTC 65

LPAL2_6:160499874-160499992	cat	tcttagctggcctagatcagcctacctgagtaatcccAAACTCCAATCCCTC 115

***************

| Primer_rv | TCCTCTGC | 23 |
| --- | --- | --- |
| KIV-3_6:160610603-160610825 | TCCTCTGCtcagctagatggctacaggctgcttgagctccatctccttgctattca | 121 |
| KIV-2_6:160616208-160616327 | TCCTCcattcagcaagatggctacagtctgcttgggctccatctccttgctat--- | 118 |
| KIV-2_6:160621754-160621873 | TCCTCcattcagcaagatggctacagtctgcttgggctccatctccttgctat--- | 118 |
| KIV-2_6:160627298-160627417 | TCCTCcattcagcaagatggctacagtctgcttgggctccatctccttgctat--- | 118 |
| KIV-2_6:160632844-160632963 | TCCTCcattcagcaagatggctacagtctgcttgggctccatctccttgctat--- | 118 |
| KIV-2_6:160638391-160638510 | TCCTCcattcagcaagatggctacagtctgcttgggctccatctccttgctat--- | 118 |
| KIV-2_6:160643938-160644057 | TCCTCcattcagcaagatggctacagtctgcttgggctccatctccttgctat--- | 118 |
| LPAL2_6:160499874-160499992 | TCCTtcattaaggaacatggctacagtccatttgggctccatctctccactct--- | 168 |
|  | **** |  |

All fw Primer binding sites

## CLUSTAL O(1.2.4) multiple sequence alignment

| Primer_fw | GAGCGCACTTTGCAGTGAGAAG | 22 |
| --- | --- | --- |
| KIV-3 | gagcgcactttgcagtgagaag | 22 |
| KIV-2B-3 | gagcgcactttgcagtgagaag | 22 |
| KIV-2-6 | gagctcactttccaatgaggaa | 22 |
| KIV-2-5 | gagctcactttccaatgaggaa | 22 |
| KIV-2-4 | gagctcactttccaatgaggaa | 22 |
| KIV-2-2 | gagctcactttccaatgaggaa | 22 |
| KIV-2-1 | gagctcactttccaatgaggaa | 22 |
| KIV-1 | ---gacactttgcagtgagg-- | 17 |
| KIV-4 | gagcacagtttccattgagaag | 22 |
| Protease | ---gacactttgcagtgagg-- | 17 |

** *** ** ****

## An additional hit (aagcagcacttgcatagaag--) is found also in KIV-5, but in wrong orientation and at a completely different position within the domain than what would be required for PCR amplification (i.e. about 120 bp downstream (in transcription direction) of a degenerated potential reverse primer binding site instead of upstream of it a).

49

All rv Primer binding sites:

CLUSTAL O(1.2.4) multiple sequence alignment

| Primer_rv | AAACTCCAATCCCTCTCCTCTGC | 23 |
| --- | --- | --- |
| KIV-3 | aaactccaatccctctcctctgc | 23 |
| KIV-2B-3 | aaactccaatccctctcctccat | 23 |
| KIV-1 | aaactccaatgtccctcctctgt | 23 |
| KIV-2-1 | aaactccaatccctctcctccat | 23 |
| KIV-2-2 | aaactccaatccctctcctccat | 23 |
| KIV-2-4 | aaactccaatccctctcctccat | 23 |
| KIV-2-5 | aaactccaatccctctcctccat | 23 |
| KIV-2-6 | aaactccaatccctctcctccat | 23 |
| KIV-4 | aaattccaatctctctcctgc-- | 21 |
| KIV-5 | aaagtccaatcactctcctctgt | 23 |
| KIV-6 | aaactccagtctctctcctcagt | 23 |
| KIV-7 | agactccagtctctctcctcagt | 23 |
| KIV-8 | aaactccagtctctctccccagt | 23 |
| KIV-9 | --aatccaatccctcccctagc- | 20 |
| KIV-10 | aaactccagtctctttcctcagt | 23 |
|  | * **** * * ** |  |

**Second round of review**

**Reviewer 1**

The authors have thoroughly revised their manuscript and adequately addressed my comments.

**Reviewer 2**

Maio et al. provided a thorough and comprehensive response to my concerns, and appears to have provided a similarly thorough response to the other two reviewers. I am especially excited by the addition of more UK Biobank results, which adds significantly to the paper’s value. I also appreciated the deeper history the authors provided on their algorithm. I was unfortunately not aware of the paper by Rosby et al. (1997).

The paper is significantly improved and I have no further concerns. I want to thank the authors for accepting my feedback so graciously and wish them well in their work.

I did notice one typo on page 3: “…excluding so confounding reads…”

**Reviewer 3**

The authors have successfully addressed all of my concerns. Below are minor suggested edits. I do not to review the authors responses to these suggested edits.

- Background section: “excluding so confounding reads from homologous regions.” The word so is unnecessary and potentially a typo.
- Discussion section: “This strategy has been originally utilized to amplify and sequence repeats as amplicon mixtures and detecting variant alike somatic mutations by aligning all data to one single repeat.” Consider replacing “alike” with as.
- I like the description “resolve intra-repeat variation” consider using in title.
- “The annotations for all VNTRs analyzed in the present manuscript expect LPA” Should expect be except?
- “Additionally, the UCSC Genome browser annotation was used to add to also intron regions the maplocus file.” Should “add to also” be “also add”?
